# Compact Rotaxane Superbases

**DOI:** 10.1021/jacs.3c01202

**Published:** 2023-04-11

**Authors:** Martin J. Power, David T. J. Morris, Iñigo J. Vitorica-Yrezabal, David A. Leigh

**Affiliations:** Department of Chemistry, University of Manchester, Oxford Road, Manchester M13 9PL, U.K.

## Abstract

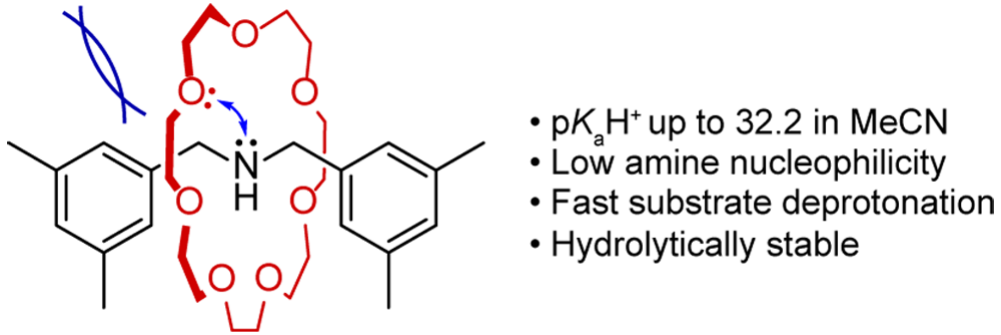

Challenges for the development of efficacious new superbases include their ease of synthesis, chemical stability, and high basicity, while minimizing nucleophilicity is important for reducing unwanted side reactions. Here, we introduce a new family of organic superbases, compact amine-crown ether rotaxanes, which show desirable characteristics in all these respects. Metal-free active template synthesis provides access to a range of rotaxanes with as little as three atoms between the stoppering groups, locking the location of a small crown ether (21C7 and 24C8 derivatives) over the amine group of the axle. The forced proximity of the interlocked protophilic components results in p*K*_a_H^+^ values as high as 32.2 in acetonitrile, which is up to 13 p*K*_a_H^+^ units greater than the p*K*_a_H^+^ values of the non-interlocked components, and brings the free base rotaxanes into the basicity realm of phosphazene superbases. The rotaxane superbases are generally chemically stable and, in a model reaction for superbases, eliminate HBr from a primary alkyl bromide with complete selectivity for deprotonation over alkylation. Their modest size, ease of synthesis, high basicity, low nucleophilicity, and, in the best cases, rapid substrate deprotonation kinetics and excellent hydrolytic stability make compact amine-crown ether rotaxane superbases intriguing candidates for potential applications in synthesis and supramolecular and materials chemistry.

## Introduction

Deprotonation of low-acidity substrates is an important process in synthesis that is commonly carried out with organic superbases.^1^ Organic superbases combine high basicity with features such as lower nucleophilicity and better solubility in apolar media than typical inorganic or organometallic complexes of similar basicity.^2^ While superbase definitions vary,^3^ a common description is that an organic superbase is a neutral organic compound with a basicity greater than that of proton-sponge (p*K*_a_H^+^ = 18.6 in MeCN).^1^ The most frequently used families of organic superbases include amidines (p*K*_a_H^+^ = 21–25),^4^ guanidines (p*K*_a_H^+^ = 23–26),^5^ and phosphazenes (p*K*_a_H^+^ = 27–43).^3,6^ While the conjugate acids of these organic superbases are often stabilized by resonance,^7^ other driving forces can be important in their design, such as aromatization in Lambert’s cyclopropenimine superbases^8^ or electron-density donation to vacant orbitals in the Verkade superbases^9^ and the singlet carbene superbases reported by Bertrand.^10^ In recent years, a number of new organic superbases have been introduced that have proved efficacious in various aspects of synthetic methodology^1,11^ and other fields of chemistry.^12−14^

**Figure 1:**
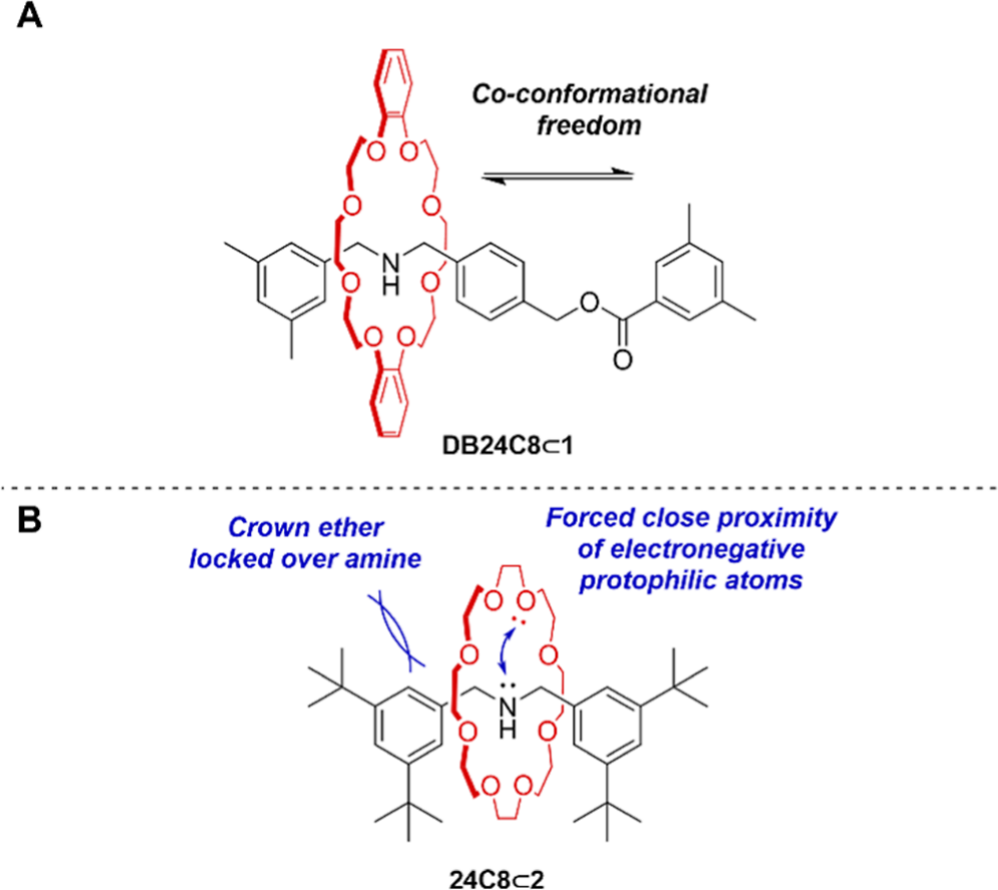
Amine-crown ether rotaxanes. (A) With space on the axle allowing co-conformational mobility of the components. (B) Compact rotaxanes, with the ring locked in place on the short axle, forcing the protophilic groups of the ring and thread into close proximity.

Hydrogen bonding between ammonium groups and crown ethers is a common way to template (pseudo)rotaxane assembly (Figure 1A).^15−19^ A number of publications^20−24^ have noted enhanced amine basicity in the corresponding rotaxanes (e.g., **DB24C8⊂1**, Figure 1A), the earliest reports emerging from the groups of Takata^20^ and Stoddart.^21^ Amine-crown ether rotaxanes derive their basicity from the strong hydrogen bonds formed between the crown ether and the resulting ammonium group (and adjacent CH groups) on the axle in the protonated form of rotaxane. In the amine form, the crown ether usually has sufficient positional flexibility along the axle that it can adopt co-conformations that avoid strong electrostatic repulsion between the nitrogen and oxygen lone pairs.^25^ However, the recently developed metal-free active template synthesis^26−30^ enables the facile synthesis, under kinetic control, of compact rotaxanes^31^ in which the location of the ring is locked in place on a very short axle, forcing the protophilic elements of the ring and thread into close proximity. Here, we report on a new family of organic superbases: compact amine-crown ether rotaxanes. In these structures, co-conformational freedom of the components is restricted, and a destabilizing effect arises from electrostatic repulsion between the proximate nitrogen and oxygen lone pairs, providing a greatly enhanced thermodynamic driving force for protonation (Figure 1B). Additionally, the restriction in co-conformational freedom leads to a high degree of preorganization when protonation occurs, further increasing the basicity of the free base rotaxane.

## Results and Discussion

**Scheme 1:**
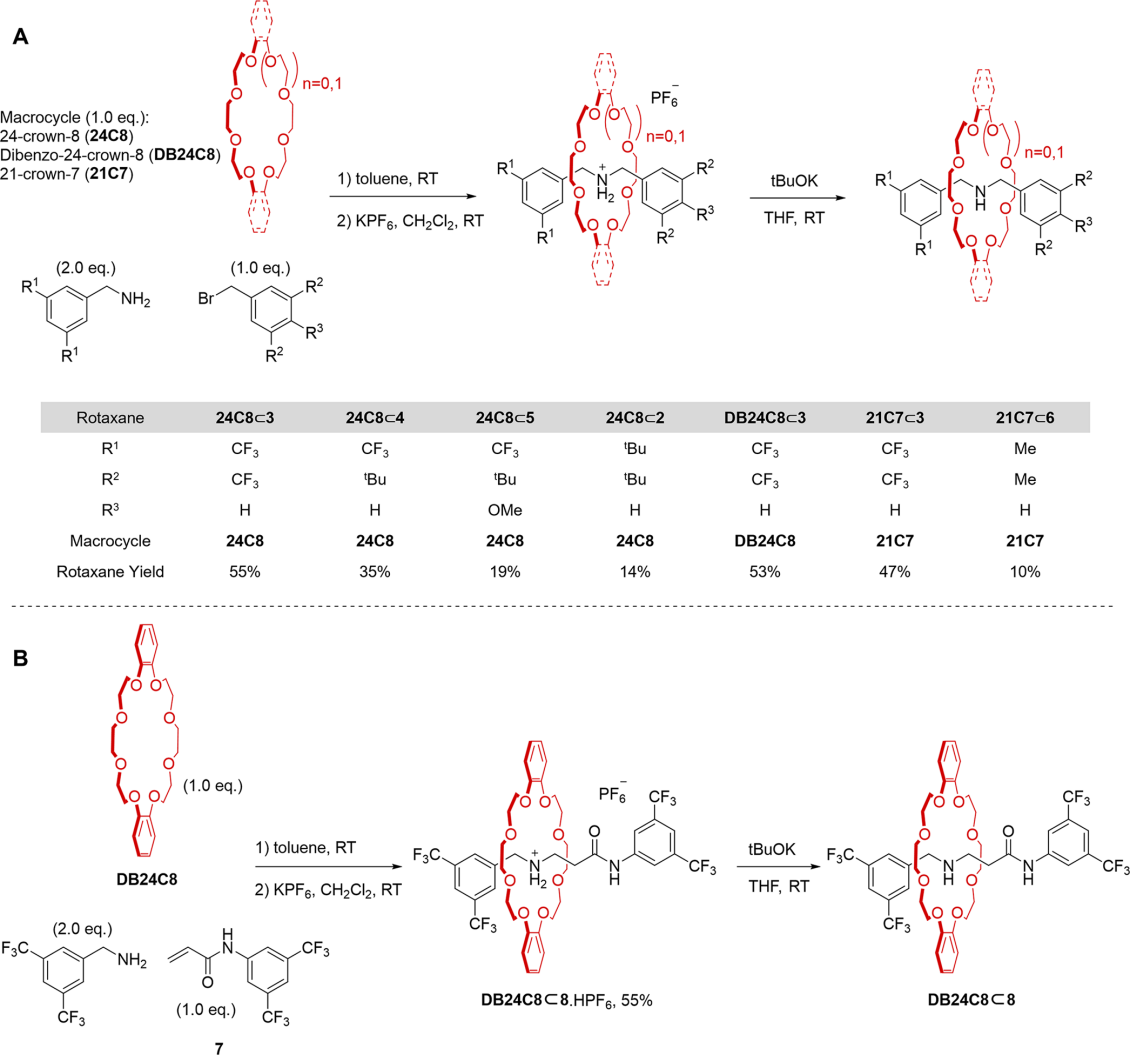
Synthesis of Rotaxane Superbases (A) Compact amine-crown ether rotaxanes. (B) Amine-crown ether rotaxane **DB24C8⊂8** with greater co-conformational freedom of the macrocycle on the axle.

We began our investigations with the rotaxane **24C8⊂3**, which was prepared in a single step as its HBr salt using metal-free active-template synthesis (Scheme 1).^26^ Liberation of the free base with potassium *tert*-butoxide in tetrahydrofuran gave the deprotonated rotaxane **24C8⊂3** in quantitative yield. The counterion exchange was completed with aqueous potassium hexafluorophosphate in methanol to afford the HPF_6_ salt, which was later used to determine the p*K*_a_H^+^ value in order to negate potential tight binding to bromide (or another counteranion).

**Figure 2:**
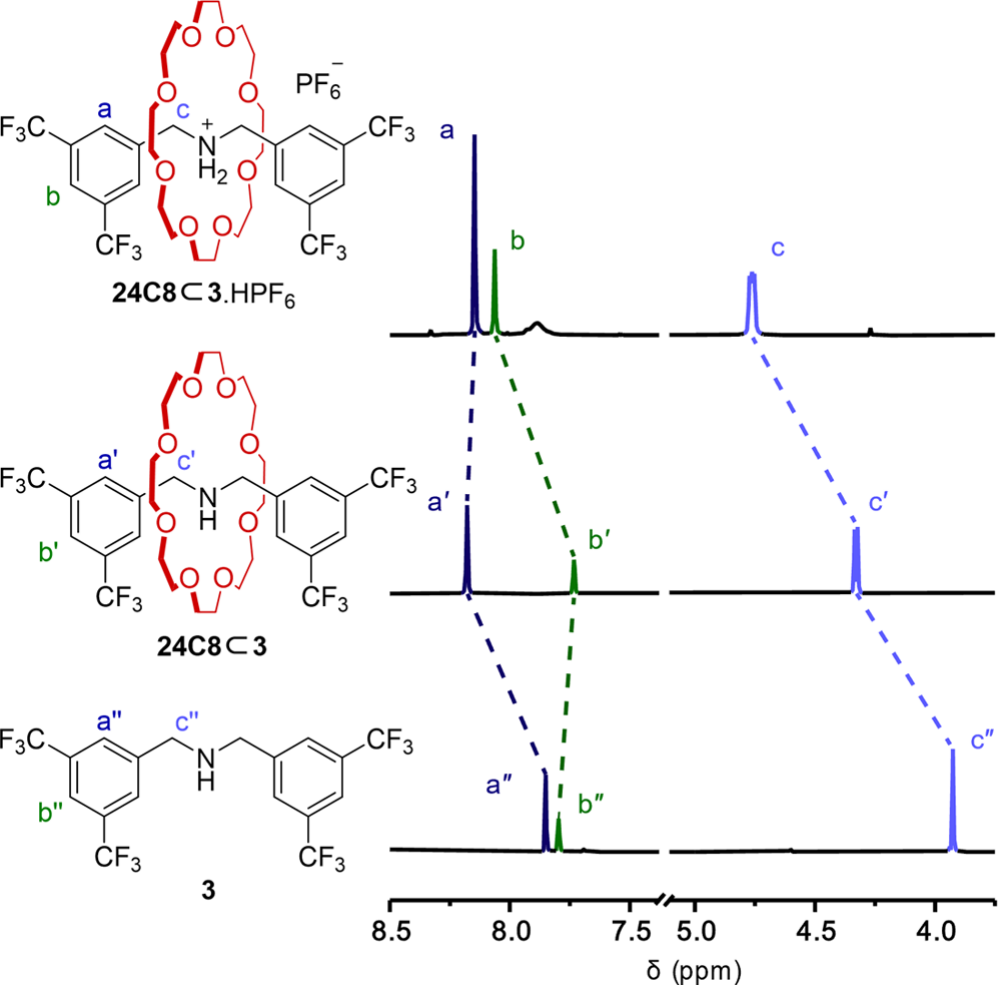
^1^H NMR of rotaxanes **24C8⊂3**·HPF_6_ and **24C8⊂3** and free thread **3** (CD_3_CN, 600 MHz, 298 K).

The ^1^H NMR spectrum of rotaxane **24C8⊂3**·HPF_6_ in CD_3_CN features signals at 4.76 (H_c_), 8.11 (H_b_), and 8.20 (H_a_) ppm (Figure 2). Upon deprotonation to **24C8⊂3**, the benzylic methylene (H_c_) signal moves substantially upfield to 4.31 ppm (Δδ 0.45 ppm; H_c′_). In comparison, in the free thread **1** (Supporting Information, Sections 1.3 and 6.0), the benzylic methylene and aromatic proton signals are at 3.90, 7.84, and 7.90 ppm, the crown ether significantly deshielding the internal region of the short axle (H_a/a′_ and H_c/c′_) in both the protonated and deprotonated forms of the compact amine-crown ether rotaxane.

Using Lambert’s method,^8^ the p*K*_a_H^+^ of **24C8⊂3** was determined as 20.5 ± 0.1 in CD_3_CN, using 1,4-diaminobutane (p*K*_a_H^+^ = 20.1 in CD_3_CN)^1^ as a reference base (see Supporting Information, Section 2.0). In contrast, the secondary amine in the free thread (**3**) has a p*K*_a_H^+^ of 12.0 ± 0.3, showing that the crown ether has a very substantial influence on the p*K*_a_H^+^ of the compact rotaxane. The p*K*_a_H^+^ value of 20.5 ± 0.1 in CD_3_CN of rotaxane **24C8⊂3** is at the lower limit of organic superbases.

**Figure 3:**
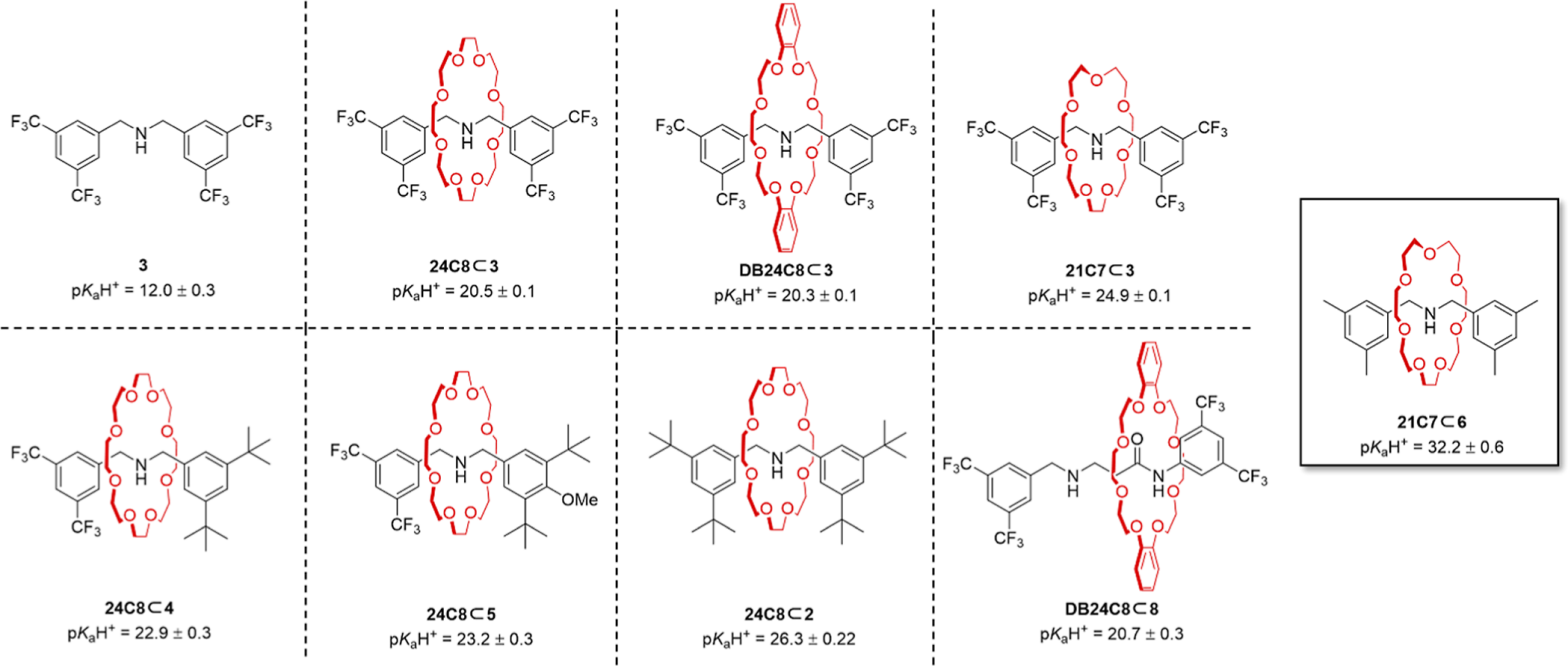
p*K*_a_H^+^ values of amine-crown ether rotaxanes and a representative thread (**3**). The p*K*_a_H^+^ values shown were determined in CD_3_CN using Lambert’s method^8^ with appropriate reference bases (see Supporting Information, Section 2.0). For p*K*_a_H^+^ values in CD_2_Cl_2_, see Supporting Information, Section 2.1.

We next prepared a series of rotaxanes with various tight macrocycles and different stoppers to see if we could further enhance the amine-crown ether rotaxane basicity. Each rotaxane was synthesized through metal-free active template synthesis (Scheme 1). The presence of electron-withdrawing units in the building blocks for the axle (particularly the nucleophile) resulted in higher yields of rotaxane, so 3,5-bis(trifluoromethyl)phenyl stoppers were chosen to probe the effect of changing the macrocycle (Figure 3).

The results of these variations (Figure 3) show that rotaxane p*K*_a_H^+^ decreases marginally to 20.3 ± 0.1 when axle **3** is enclosed by a dibenzo-24-crown-8 macrocycle (rotaxane **DB24C8⊂3**), despite 24-crown-8 binding dibenzylammonium threads substantially stronger than their dibenzo-24-crown-8 homologues.^32^ The enthalpic effect of dibenzylammonium binding appears to be less important for the resulting p*K*_a_H^+^ than the increased preorganization and rigidity of the dibenzo-crown and the consequences that that has on lone pair repulsion between the thread and the macrocycle. The p*K*_a_H^+^ increases dramatically to 24.9 ± 0.1 when a smaller macrocycle 21-crown-7 (rotaxane **21C7⊂3**) is used (Figure 3). Comparing the p*K*_a_H^+^ values of thread **3** with rotaxane **21C7⊂3**, this corresponds to a 10^13^-fold increase in the basicity of the secondary amine upon encapsulation with a 21-crown-7 ring.

We also varied the functional groups on the axle stoppers (Figure 3). Using 24-crown-8 as the standard macrocycle, replacing the trifluoromethyl group with electron-rich *tert*-butyl groups on one stopper (rotaxane **24C8⊂4**) resulted in a p*K*_a_H^+^ increase from 20.5 ± 0.1 to 22.9 ± 0.1 (Figure 3). Adding a *p*-methoxy unit to one of the stoppers (rotaxane **24C8⊂5**) gave a marginal increase in p*K*_a_H^+^ to 23.2 ± 0.2, but at the expense of a lower rotaxane yield (19% cf. 55% for **24C8⊂3**; Scheme 1A). Replacing all of the trifluoromethyl groups on both stoppers with *tert*-butyl groups (rotaxane **24C8⊂2**) resulted in a significant p*K*_a_H^+^ enhancement to 26.3 ± 0.2, although the isolated rotaxane yield was reduced (14%; Scheme 1A) due to competition with a relatively fast noninterlocked thread-forming reaction.

Rotaxane **DB24C8⊂8** (Figure 3) was synthesized with increased space on the axle to allow an amide to be present (the effect of a secondary binding site on rotaxane basicity has previously been noted by Credi^24,33^). The p*K*_a_H^+^ of rotaxane **DB24C8⊂8** was measured to be 20.7 ± 0.3 in CD_3_CN. This is slightly higher than that of **DB24C8⊂3**, with the decrease in p*K*_a_H^+^ due to the second binding site and slightly longer axle apparently compensated for by the replacement of a 3,5-bis(trifluoromethyl)phenyl stopper with a more electron-rich alkyl group. Rotaxane **21C7⊂6**, featuring the tightest macrocycle in the series with electron-rich stoppers, was synthesized in one step in a modest 10% yield. The p*K*_a_H^+^ of **21C7⊂6** was measured to be 32.2 ± 0.6 in CD_3_CN, more basic than many phosphazene superbases^2^ and similar to Verkade superbases.^9^

We also measured the basicity of an amine-crown ether rotaxane in CD_2_Cl_2_, a less competitive solvent than CD_3_CN with regard to hydrogen bonding (Supporting Information, Section 2.2). We found that the p*K*_a_H^+^ values of the compact rotaxane superbases appeared to be increased in the less polar solvent relative to conventional superbases.

**Figure 4:**
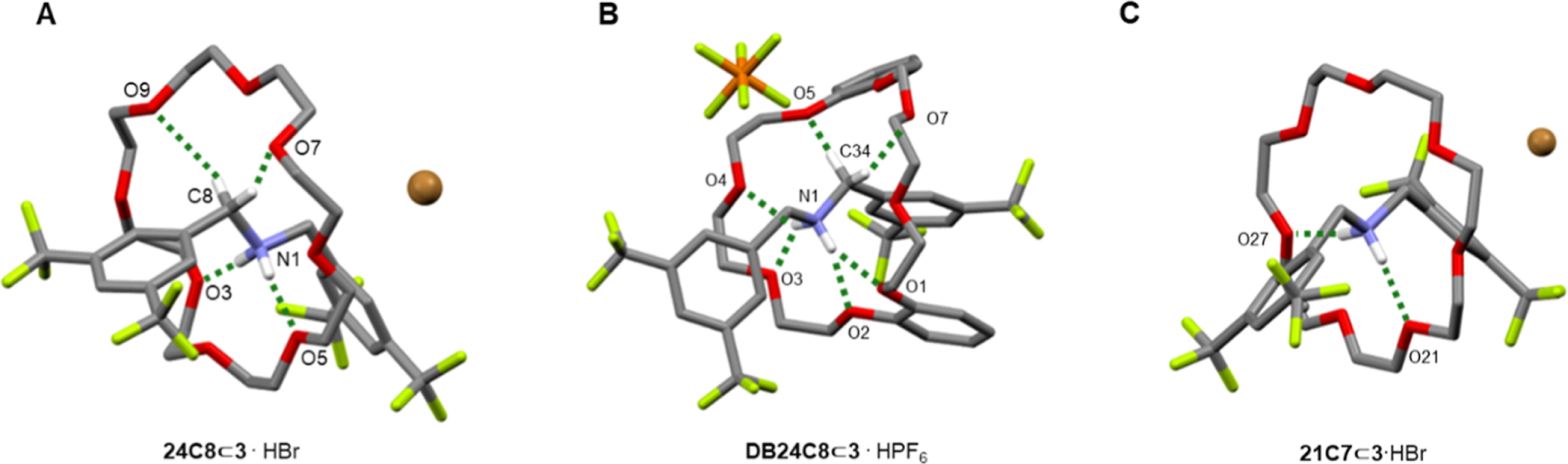
X-ray crystal structures of compact amine-crown ether rotaxanes (A) **24C8⊂3**·HBr, (B) **DB24C8⊂3**·HPF_6_, and (C) **21C7⊂3**·HBr. Hydrogen bond lengths (Å): **24C8⊂3**·HBr, N1–H···O5 = 2.06, N1–H···O3 = 1.96, C8–H···O9 = 2.61, C–H···O7 = 2.46; **DB24C8⊂3**·HPF_6_, N1–H···O1 = 2.23, N1–H···O2 = 2.24, N1–H···O3 = 2.25, N1–H···O4 = 2.21, C34–H···O5 = 2.37, C–H···O7 = 2.33; and **21C7⊂3**·HBr (averaged over 8 rotaxanes in the unit cell), N1–H···O21 = 2.01, N1–H···O27 = 2.11. Hydrogen bond angles (deg): **24C8⊂3**·HBr, N1–H···O5 = 170.8, N1–H···O3 = 172.37, C8–H···O9 = 147.8, C–H···O7 = 159.1; **DB24C8⊂3**·HPF_6_, N1–H···O1 = 148.7, N1–H···O2 = 141.0, N1–H···O3 = 143.7, N1–H···O4 = 139.9, C34–H···O5 = 145.4, C–H···O7 = 140.6; and **21C7⊂3**·HBr (averaged over 8 rotaxanes in the unit cell), N1–H···O21 = 166.3, N1–H···O27 = 156.1. Non-hydrogen bonded hydrogen atoms are omitted for clarity.

Single crystals suitable for investigation by X-ray diffraction were obtained from samples of rotaxanes **24C8⊂3**·HBr, **DB24C8⊂3**·HPF_6_, and **21C7⊂3**·HBr (Figures 4 and S3–S5). The solid-state structure of **24C8⊂1**·HBr (Figure 4A) shows four N–H···O hydrogen bonds between the ammonium N–Hs and crown ether oxygens. Each N–H forms a short (1.96 and 2.06 Å), almost linear (170.8 and 172.4°), hydrogen bond with a glycolic oxygen, with a longer (2.50 and 2.55 Å) interaction to a second oxygen atom. In the solid-state structure of **DB24C8⊂3**·HPF_6_ (Figure 4B), there are four bifurcated^34^ intercomponent N–H···O hydrogen bonds, together with two C–H···O hydrogen bonds (2.37, 2.33 Å; 145.4, 140.6°) from one of the −N^+^CH_2_– groups to the crown ether. The **DB24C8** macrocycle is folded to allow π-stacking between an electron-rich catechol of the crown ether and one of the electron-deficient 3,5-bis(trifluoromethyl)aryl stopper groups of the axle (Figure 4B). These intercomponent interactions likely contribute to the high thermodynamic stability of the protonated structure and, therefore, the large p*K*_a_H^+^ value.

The asymmetric unit of the X-ray crystal structure of **21C7⊂3**·HBr contains 8 rotaxanes (Figure S5). Each features intercomponent hydrogen bonds similar to that of **24C8⊂3**·HBr with hydrogen bonds formed between the N–Hs and several glycolic oxygens (Figure 4C). The N–H···O hydrogen bonds are slightly longer (2.01 and 2.11 cf. 1.96 and 2.06) than in **24C8⊂3**·HBr, which could imply that the hydrogen bonds in **21C7⊂3**·HBr are formally weaker than that of **24C8⊂3**·HBr, despite **21C7⊂3** being over 10^5^ times more basic than **24C8⊂3**. The superior basicity of **21C7⊂3** may be more a consequence of the steric congestion inhibiting co-conformational change to relieve lone pair repulsion in the free-base rotaxane, rather than the magnitude of particularly strong attractive intercomponent interactions stabilizing the conjugate acid. The influence of C–H···O hydrogen bonding is difficult to ascertain in the constricted environment of **21C7⊂3**·HBr. However, it is probably somewhat less stabilizing than in typical diarylammonium rotaxanes as no linear hydrogen bonds C–H···O are observed in the solid-state structure.

Characteristics of organic superbases that can be particularly advantageous for practical applications include (i) fast kinetics of deprotonation of other species, (ii) low nucleophilicity, and (iii) good hydrolytic stability. We investigated these properties in some illustrative experiments that compared the rotaxane efficacy in these respects to that of some common conventional organic superbases, 1,8-diazabicyclo(5.4.0)undec-7-ene (DBU), 7-methyl-1,5,7-triazabicyclo(4.4.0)dec-5-ene, and phosphazene base *tert*-butylimino-tris(dimethylamino)phosphorane (P_1_-^*t*^Bu) (Tables 1 and 2).

**Table 1:**
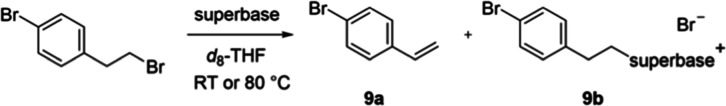
Deprotonation Kinetics and Protonation vs Alkylation of Compact Amine-Crown Ether Rotaxane and Conventional Organic Superbases

superbase	p*K*_a_H^+^ (MeCN)	E2/S_N_2 ratio	half-life (h) (25 °C)	half-life (h) (80 °C)
**24C8⊂3**	20.5	>99:1	176	
**DB24C8⊂3**	20.3	>99:1	33	
**21C7⊂3**	24.9	>99:1	>5000	4073
**24C8⊂2**	26.3	>99:1	>5000	260
**DB24C8⊂8**	20.7	>99:1	10	
**21C7⊂6**	32.2	>99:1	0.15	
DBU	24.3^1^	87:13	635	
mTBD	25.5^1^	>99:1	115	
P_1_-^*t*^Bu	27.0^1^	>99:1	419	

**Table 2:**
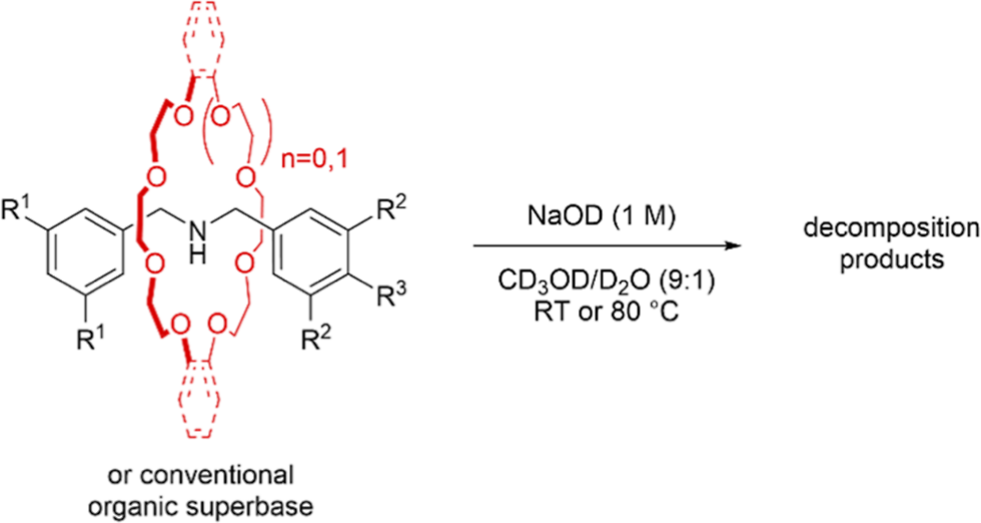
Rate of Hydrolytic Decomposition of Rotaxanes and Conventional Organic Superbases

superbase	p*K*_a_H^+^ (MeCN)	half-life (25 °C)	half-life (80 °C)
**24C8⊂3**	20.5	>7 days	>7 days
**DB24C8⊂3**	20.3	>7 days	>7 days
**21C7⊂3**	24.9	<5 min	
**24C8⊂2**	26.3	6 h	
**DB24C8⊂8**	20.7	>7 days	>7 days
**21C7⊂6**	32.2	>7 days	>7 days
DBU	24.3^1^	11 h	
mTBD	25.5^1^	>7 days	95 h
P_1_-^*t*^Bu	27.0^1^	>7 days	>7 days

The base-mediated elimination of HBr from 4-bromophenethyl bromide to form 4-bromostyrene **9a** was chosen to exemplify relative rates of deprotonation by the superbases and their tendency for promoting elimination over nucleophilic addition as an unwanted side reaction (Tables 1 and S1). A primary alkyl bromide was used as the substrate so that the competing S_N_2 nucleophilic addition to form **9b** provided a reasonable kinetic and thermodynamic alternative to E2 elimination.^35−37^ None of the rotaxane superbases, and only DBU of the conventional superbases, yielded any **9b** in this experiment (Table 1). The resistance of the rotaxanes to alkylation is unsurprising, given the steric hindrance of the basic nitrogen atom encapsulated by the crown ether.

Two of the rotaxanes, **21C7⊂3** and **24C8⊂2**, produced only traces of eliminated product **9a** with reaction times of over a week at 25 °C (Table 1). Even at 80 °C, the formation of **9a** was slow, suggesting that no low-energy routes exist with these two rotaxanes for a proton to be transferred to the buried nitrogen atom on the axle. In contrast, all of the other rotaxane superbases deprotonate 4-bromophenethyl bromide faster than any of the conventional organic superbases, in the case of **21C7⊂6**, several thousand times faster (Table 1). Although **21C7⊂6** is the most basic rotaxane in the series and exhibits the fastest deprotonation kinetics for the alkyl bromide substrate, the deprotonation rates do not otherwise correspond to the relative rotaxane p*K*_a_H^+^ values. The rapid deprotonation kinetics of several of the rotaxane superbases suggests that particular aspects of their structure enable co-conformational dynamics, possibly involving crown ether oxygens transiently receiving the proton, involved in the deprotonation mechanism. Studies to uncover the details of the acting mechanism(s) are ongoing.

The hydrolytic stability of the rotaxane superbases and conventional organic superbases was explored by studying their decomposition in a 1 M NaOD solution in 9:1 v/v CD_3_OD/D_2_O with 2 mol % pivalic acid as an internal standard (Tables 2 and S2). Four of the trialed rotaxanes showed no evidence of any degradation under these conditions, even at 80 °C for more than a week. Rotaxane **24C8⊂2** significantly decomposed over a few hours at room temperature (*t*_1/2_ = 6 h), and **21C7⊂3** was consumed completely within a matter of minutes (Table 2). The poor hydrolytic stability of **21C7⊂3** is particularly curious: **21C7⊂6**, which features the same, extremely tight (and strained on the axle) macrocycle, is stable, as is axle **3** when encapsulated by other macrocycles (e.g., rotaxanes **24C8⊂3** and **DB24C8⊂3**). What makes components **21C7** and **3** particularly reactive in hydrolytic media when threaded is unclear. We note that the two rotaxanes with very slow kinetics for deprotonation of 4-bromophenethyl bromide are also the two rotaxanes that are hydrolytically unstable. Apart from these two examples, the hydrolytic stability of the rotaxane superbases compares favorably to DBU and mTBD and is similar to P_1_-^*t*^Bu (Table 2).

## Conclusions

In summary, we have synthesized and carried out preliminary studies of the properties of a new family of organic superbases, compact amine-crown ether rotaxanes with as little as three atoms between the axle stoppers. The rotaxane superbases are prepared by kinetically controlled metal-free active-template synthesis, generally in one step from commercially available building blocks, in 10–55% yield. Their p*K*_a_H^+^ values vary with crown ether size and rigidity (smaller, more rigid macrocycles tend to result in more basic rotaxanes) and the functionality of the dibenzylamine stoppers (electron-rich substituents tend to result in more basic rotaxanes). Two of the compact rotaxanes studied, **24C8⊂2** and **21C7⊂3**, deprotonate substrates much more slowly than the other rotaxanes in the series. These two rotaxanes also decompose quickly in hydrolytic media, while all the other rotaxane superbases are chemically stable. The reasons for the difference in chemistry between **24C8⊂2** and **21C7⊂3** and the other rotaxane superbases studied remain unclear. However, the most basic rotaxane measured, **21C7⊂6**, shows excellent chemical stability under such conditions and has a p*K*_a_H^+^ of 32.2 ± 0.6 in CD_3_CN, a basicity ∼13 orders of magnitude higher than either of its non-interlocked components. The modest size, ease of synthesis, high basicity, low nucleophilicity, and, in the best cases, rapid deprotonation kinetics and excellent hydrolytic stability of compact amine-crown ether rotaxanes are attractive characteristics for potential applications in organic synthesis and other fields of chemistry. They add to the growing list of remarkable property effects that can be introduced through the nature of the mechanical bond.^15^

## Supporting Information Available

Synthetic schemes, experimental procedures, compound characterization data, details of p*K*_a_H^+^ determination, X-ray crystallography, and ^1^H and ^13^C NMR spectra of novel compounds (PDF)

## Abbreviations

NMR: nuclear magnetic resonance (spectroscopy)
DBU: 1,8-diazabicyclo(5.4.0)undec-7-ene
mTBD: 7-methyl-1,5,7-triazabicyclo(4.4.0)dec-5-ene
P^_1_^-^*t*^Bu: *tert*-butylimino-tris(dimethylamino)phosphorane
THF: tetrahydrofuran
RT: room temperature
