# Supplementary material for: Compact Rotaxane Superbases
Source: J Am Chem Soc. 2023 Apr 11;145(15):8593–9. doi: 10.1021/jacs.3c01202 (PMC10119927; doi:10.1021/jacs.3c01202)
Supplement: Supplementary file 1 — ja3c01202_si_001.pdf [file ja3c01202_si_001.pdf]

Supplementary Information

Compact Rotaxane Superbases

Martin J. Power,‡ David T. J. Morris‡, Iñigo J. Vitorica-Yrezabal and David A. Leigh*

Department of Chemistry, University of Manchester, Oxford Road, Manchester M13 9PL, United Kingdom

Email: david.leigh@manchester.ac.uk

Contents

1.0 Supplementary Methods 2

1.1 General Information 2

1.2 Synthetic Schemes 3

1.3 Experimental Procedures 4

1.3.1 General Procedures 4

1.3.2 Synthetic Procedures and Characterization 5

2.0 pK_aH$^+$ Measurements 16

2.1 pK_aH$^+$ Measurement Using More than one Reference Base 19

2.2 pK_aH$^+$ Measurements in Alternative Solvents 20

3.0 Protonation Versus Alkylation Studies 21

4.0 Hydrolytic Decomposition Experiments 23

5.0 X-Ray Crystallography 25

6.0 NMR Spectra of Novel Compounds 29

7.0 Abbreviations 45

8.0 References 45

‡These authors contributed equally.

1.0 Supplementary Methods

1.1 General Information

Where specified, procedures were performed under an atmosphere of nitrogen. Air and moisture-sensitive liquids/solutions were transferred to reaction vessels by syringe under an atmosphere of nitrogen. Solvents and reagents were purchased from commercial suppliers and were used without further purification unless otherwise specified. Agitation was achieved using Teflon coated stirrer bars by magnetic induction. Deionized water was obtained by a milli-Q water purifier (Millipore). Anhydrous solvents were obtained by passing the solvent through an activated alumina column on a Phoenix SDS (solvent drying system; JC Meyer Solvent Systems, CA, USA). All thin layer chromatography (TLC) experiments were conducted on precoated silica gel plates (0.25 mm thick, 60 F254, Merck, Germany) and visualized using ultraviolet light (254 nm) or staining. Flash column chromatography was carried out using Silica 60 Å (particle size 40–63 µm, Sigma Aldrich, UK) as the stationary phase. Size exclusion chromatography was performed using Bio-Rad Bio-Beads® S-X3 Beads. Preparatory TLC was performed using Merck glass-backed PLC silica gel 60 plates with specified layer thickness and eluent. ^{1}H and ^{13}C NMR spectra were recorded on a Bruker Avance III instrument with an Oxford AS600 magnet equipped with a cryoprobe [5 mm CPDCH ^{13}C-^{1}H/D] (600 MHz) at a constant temperature of 20 °C and 1 atm unless otherwise specified. ^{1}H and ^{13}C chemical shifts are reported in parts per million (ppm) from low to high field and referenced to the literature values for chemical shifts of residual non-deuterated solvent, with respect to tetramethylsilane (0.00 ppm) as an external standard. Multiplicity is reported as follows – s = singlet, d = doublet, t = triplet, q = quartet, m = multiplet, spt = septet. ^{13}C chemical shifts are reported as singlets unless stated otherwise. All spin-spin coupling constants (*J*) are reported in hertz (Hz) to the nearest 0.1 Hz. Low-resolution ESI mass spectrometry was performed with a Thermo Scientific LCQ Fleet Ion Trap Mass Spectrometer or an Agilent Technologies 1200 LC system with an Advion Expression LCMS single quadrupole MS detector. High-resolution mass spectrometry (HRMS) was carried out at the Mass Spectrometry Service, Department of Chemistry, University of Manchester.

1.2 Synthetic Schemes

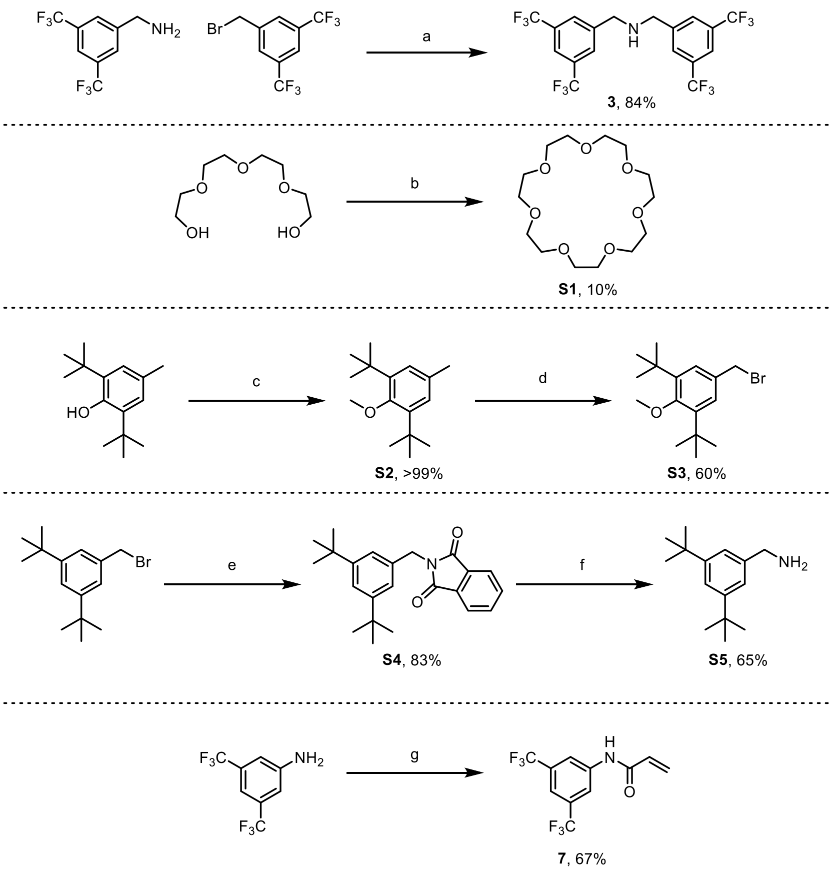

Scheme S1. Synthesis of **3**, **7, S1**-**S5**. Reagents and conditions: (a) MeCN, 80 °C, 24 h; (b) sodium hydride (2.2 equiv.), triethyleneglycol di(*p*-toluenesulfonate) (1.0 equiv.), THF, 0 °C to RT, 72 h; (c) iodomethane (1.5 equiv.), K_2CO_3 (3.0 equiv.), DMF, RT, 16 h; (d) NBS (1.1 equiv.), AIBN (0.3 equiv.), benzene, reflux, 16 h; (e) phthalimide (1.5 equiv.), K_2CO_3 (3.0 equiv.), DMF, RT, 16 h; (f) hydrazine monohydrate, THF, RT, 3 h; (g) Acryloyl chloride (1.1 equiv.), triethylamine (1.1 equiv.), $CHCl_3$, 0 °C to RT, 30 mins.

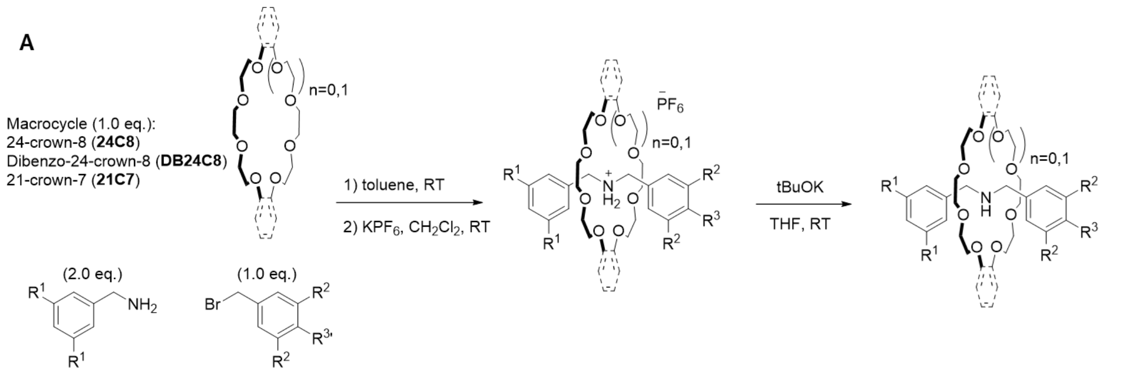

Rotaxane	**24C8⊂3**	**24C8⊂4**	**24C8⊂5**	**24C8⊂2**	**DB24C8⊂3**	**21C7⊂3**	**21C7⊂6**
R^1	CF_3	CF_3	CF_3	^{t}Bu	CF_3	CF_3	Me
R^2	CF_3	^{t}Bu	^{t}Bu	^{t}Bu	CF_3	CF_3	Me
R^3	H	H	OMe	H	H	H	H
Macrocycle	**24C8**	**24C8**	**24C8**	**24C8**	**DB24C8**	**21C7**	**21C7**
Rotaxane Yield	55%	35%	19%	14%	53%	47%	10%

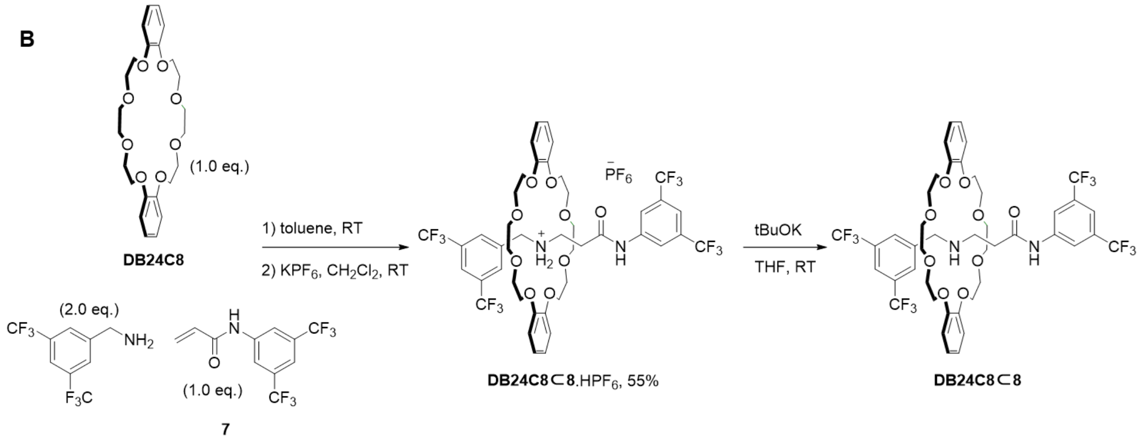

Scheme S2. Synthesis of rotaxane superbases. Reagents and conditions: (a) toluene, RT, 16–72 h; (b) sat. KPF_6 (aq.), CH_2Cl_2 RT, 16 h; (c) ^{t}BuOK (5.0 equiv.), THF, RT, 15 mins.

1.3 Experimental Procedures

1.3.1 General Procedures

General Procedure A – Rotaxane formation from stoppered benzylamines, a crown ether and a stoppered benzyl bromide

To an oven-dried vial was added benzylamine (2.0 equiv., 0.3 M) under an inert atmosphere. Crown ether (82 µM, 1.0 equiv.), anhydrous toluene (0.27 mL) and benzyl bromide (82 µM, 1.0 equiv.) were added and the resultant solution was stirred at the specified temperature for 16–72 hours. The crude rotaxane was then purified by size exclusion chromatography (S-X3 beads, CH_2Cl_2 eluent) followed by preparatory TLC (SiO_2, 500 µm plate, 6:4:1 EtOAc:CH_2Cl_2:MeOH eluent).

General Procedure B – PF_6 Anion exchange procedure

To a solution of rotaxane HBr salt (1.0 equiv., 1 mM) in MeOH (1.20 mL) was added 0.4 M aqueous KPF_6 (0.14 mL, 5.0 equiv.). The resultant solution was stirred for 16 h and concentrated under reduced pressure. The residue was dissolved in CH_2Cl_2 (5.00 mL) and filtered. The filtrate was then concentrated to give the rotaxane HPF_6 salt.

General Procedure C – Deprotonation of rotaxane superbase conjugate acids

Under a dry, inert atmosphere, to a solution of rotaxane HPF_6 salt (1.0 equiv., 10 mM) in anhydrous THF (0.4 mL) was added potassium *tert*-butoxide (4 mg, 10.0 equiv.) and the resultant suspension was stirred for 30 minutes at room temperature. The solution was filtered via cannula into a separate, oven-dried flask under inert atmosphere. The residue was washed and filtered twice with anhydrous THF (2 × 1 mL). The combined filtrates were concentrated under reduced pressure and the residue was dissolved in either anhydrous d_8-THF or anhydrous d_3-MeCN. The resultant solution was transferred via syringe to an oven-dried Young's NMR tube under inert atmosphere.

1.3.2 Synthetic Procedures and Characterization

1.3.2.1 Rotaxane Precursors and Control Compounds

Bis(3,5-bis(trifluoromethyl)benzyl)amine, 3

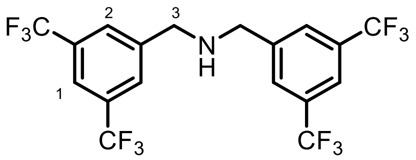

3,5-bis(trifluoromethyl)benzylamine (100 mg, 0.42 mmol, 2.0 equiv.) and 3,5-bis(trifluoromethyl)benzyl bromide (64 mg, 0.21 mmol, 1 equiv.) were dissolved in CH_3CN (20 mL). The resulting mixture was heated to 80 °C and stirred for 16 hours. The solution was concentrated under reduced pressure and purified by flash column chromatography (SiO_2, 100:2:1 CH_2Cl_2:MeOH:NEt_3) to give the desired amine **3** as yellow viscous oil (960 mg, 0.35 mmol, 84%). **^{1}H NMR** (600 MHz, CD_3CN) δ_H 3.92 (s, 4H, 2 × C^3H_2), 7.84 (s, 2H, 2 × C^1H), 7.90 (s, 4H, 4 × C^2H). Spectroscopic data matched that previously reported.[1]

21-Crown-7, S1

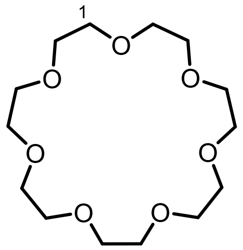

Under a dry, inert atmosphere, anhydrous THF (800 mL) was cooled to 0 °C in a 2.5 L round-bottomed flask for 15 minutes. To the flask was added NaH (60% dispersion in mineral oil, 879 mg, 21.98 mmol, 2.2 equiv.). To the resultant suspension was added simultaneously a solution of tetraethyleneglycol (1.73 mL, 9.99 mmol, 1.0 equiv., 12 mM) in anhydrous THF (20 mL) and triethyleneglycol di(*p*-toluenesulfonate) (4580 mg, 9.99 mmol, 1.0 equiv.) in anhydrous THF (20 mL) dropwise over 4 hours using a syringe pump. The resultant suspension was stirred at room temperature for 48 hours. The suspension was filtered and the filtrate was concentrated under reduced pressure. The residue was dissolved in CH_2Cl_2 (100 mL) and the resultant solution was washed with brine (100 mL) and deionized water (100 mL), dried over $MgSO_4$, filtered and concentrated under reduced pressure. The crude product was then purified by flash column chromatography (SiO_2, 5:95 to 10:90 MeOH:CH_2Cl_2) to give the title crown ether **S1** as a colorless oil (310 mg, 1.00 mmol, 10%). **^{1}H NMR** (600 MHz, $CDCl_3$) δ_H 3.54 (s, 28H, 14 × C^1H_2). Spectroscopic data matched that previously reported.[2]

2,6-Bis(*tert*-butyl)-4-methylanisole, S2

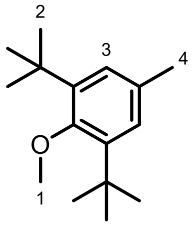

Under a dry, inert atmosphere, 2,6-bis(*tert*-butyl)-4-methylphenol (4.68 g, 20.00 mmol, 1.0 equiv., 0.1 M) was dissolved in DMF (200 mL). To the resultant solution was added potassium carbonate (4831 mg, 60.00 mmol, 3.0 equiv.) and methyl iodide (1.87 mL, 30.00 mmol, 1.5 equiv.). The solution was heated to 80 °C and stirred for 16 h. The solution was cooled to room temperature and concentrated under reduced pressure. The residue was dissolved in Et_2O (300 mL) and washed with aqueous LiCl (5% w/w, 500 mL), deionized water (300 mL) and brine (300 mL). The organic layer was dried over $MgSO_4$, filtered and concentrated under reduced pressure to give the title anisole **S2** as a colorless oil (4.69 g, 20.00 mmol, >99%). **^{1}H NMR** (600 MHz, $CDCl_3$) δ_H 1.48 (s, 18H, 6 × C^2H_3), 2.34 (s, 3H, C^4H_3), 3.73 (s, 3H, C^1H_3), 7.10 (s, 2H, 2 × C^3H). Spectroscopic data matched that previously reported.[3]

3,5-Bis(*tert*-butyl)-4-methoxybenzyl bromide, S3

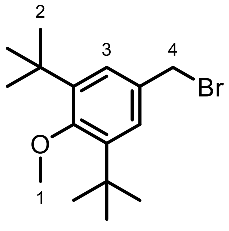

Under a dry, inert atmosphere, **S2** (3600 mg, 15.36 mmol, 1.0 equiv., 0.1 M) was dissolved in anhydrous benzene (154 mL). To the resultant solution was added *N*-bromosuccinimide (5468 mg, 30.72 mmol, 2.0 equiv.) and azoisobutyronitrile (1261 mg, 7.68 mmol, 0.5 equiv.). The solution was heated to reflux and stirred for 16 hours. The solution was cooled to room temperature and quenched with saturated aqueous $Na_2S_2O_3$ (150 mL). The organic layer was washed with deionized water (100 mL)

and brine (100 mL), dried over $MgSO_4$, filtered and concentrated under reduced pressure. The crude product was purified by flash column chromatography (SiO_2, 1:9 to 3:7 Et_2O:PE) to give the desired benzyl bromide **S4** as a colorless oil (2881 mg, 9.20 mmol, 60%). **^{1}H NMR** (600 MHz, $CDCl_3$) δ_H 1.43 (s, 18H, 6 × C^2H_3), 3.69 (s, 3H, C^1H_3), 3.80 (s, 2H, C^4H_2), 7.18 (s, 2H, 2 × C^3H). Spectroscopic data matched that previously reported.[4]

N-(3,5-bis(*tert*-butyl)benzyl)phthalimide, S4

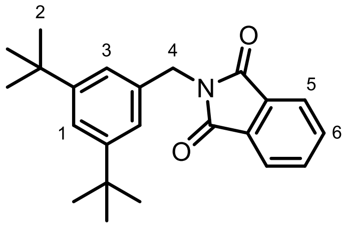

Under an inert atmosphere, 3,5-bis(*tert*-butyl)benzyl bromide (1.00 g, 3.53 mmol, 1.0 equiv., 0.1 M) was dissolved in DMF (35 mL). Potassium phthalimide (850 mg, 4.59 mmol, 1.3 equiv.) and potassium carbonate (732 mg, 5.30 mmol, 1.5 equiv.) were added and the resultant suspension was stirred at room temperature for 16 hours. The residue was dissolved in Et_2O (100 mL) and washed with aqueous LiCl (5% w/w, 100 mL), deionized water (100 mL) and brine (100 mL). The organic layer was dried over $MgSO_4$, filtered and concentrated under reduced pressure to give the title phthalimide **S4** as a colorless powder (1.02 g, 2.92 mmol, 83%). **^{1}H NMR** (600 MHz, CD_3CN) δ_H 1.31 (s, 18H, 6 × C^2H_3), 4.82 (s, 2H, C^4H_2), 7.29 (s, 2H, 2 × C^3H), 7.34 (s, 1H, C^1H), 7.69 (dd, J = 3.0, 5.4, 2H, 2 × C^6H), 7.84 (dd, J = 3.0, 5.4, 2H, 2 × C^5H). Spectroscopic data matched that previously reported.[5]

3,5-Bis(*tert*-butyl)benzylamine, S5

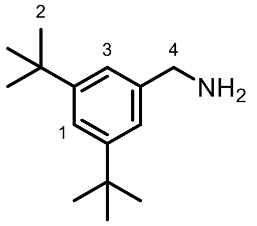

Under an inert atmosphere, **S4** (300 mg, 0.86 mmol, 1.0 equiv., 0.2 M) in was dissolved in THF (4.3 mL). To the resultant solution was added hydrazine monohydrate (0.25 mL, 5.15 mmol, 6.0 equiv.). The solution was heated to 70 °C and stirred for 3 hours, after which a white precipitate formed. The solution was cooled to room temperature and filtered. Deionized water (10 mL) was added to the filtrate, which was extracted with CH_2Cl_2 (3 × 10 mL). The combined organic extracts were dried over $MgSO_4$, filtered and concentrated under reduced pressure to give the title benzylamine **S5** as a colorless powder (123 mg, 0.56 mmol, 65%). **^{1}H NMR** (600 MHz, CD_3CN) δ_H 1.31 (s, 18H, 6 × C^2H_3), 3.74 (s, 2H, C^4H_2), 7.18 (s, 2H, 2 × C^3H), 7.31 (s, 1H, C^1H). Spectroscopic data matched that previously reported.[5]

N-Bis(3,5-bis(trifluoromethyl)phenyl)acrylamide, 7

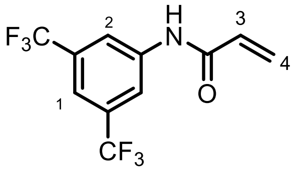

3,5-bis(trifluoromethyl)aniline (1000 mg, 4.3 mmol, 1.0 equiv.) and triethylamine (0.67 mL, 4.8 mmol, 1.1 equiv.) was dissolved in $CHCl_3$ (20 mL) and cooled to 0 °C. Acryloyl chloride (0.39 mL, 4.8 mmol, 1.1 equiv.) was added dropwise and the resulting mixture was allowed to warm to room temperature and stirred for 30 minutes. The solution was concentrated under reduced pressure and purified by flash column chromatography (SiO_2, 1:7 EtOAc:hexane) to give the desired acrylamide **7** as white powder (850 mg, 2.88 mmol, 67%). **^{1}H NMR** (600 MHz, $CDCl_3$) δ_H 5.73 (dd, J = 10.2, 1.4, 1H, C^4H_{cis}), 6.29 (dd, J = 16.8, 10.2 Hz, 1H, C^3H), 6.44 (dd, J = 16.8, 1.3 Hz, 1H, C^4H_{trans}), 7.19 (t, J = 1.8 Hz, 1H, C^1H), 7.44-7.53 (m, 2H, C^2H), 7.61 (s, 1H, NH). Spectroscopic data matched that previously reported.[6]

1.3.2.2 Rotaxanes

Bis(3,5-bis(trifluoromethyl)benzyl)ammonium{24-crown-8} hexafluorophosphate, 24C8⊂3•HPF_6

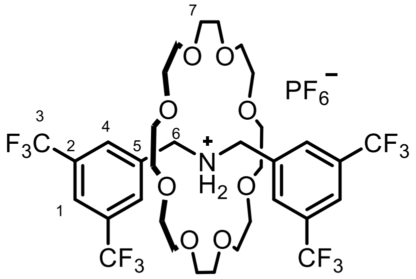

Synthesized according to general procedure A using 3,5-bis(trifluoromethyl)benzylamine (50 mg, 0.21 mmol, 2.0 equiv.), 24-crown-8 (37 mg, 0.11 mmol, 1.0 equiv.) and 3,5-bis(trifluoromethyl)benzyl bromide (32 mg, 0.11 mmol, 1.0 equiv.). The reaction was stirred at room temperature for 16 hours. The pure HBr salt was subjected to general procedure B and the title rotaxane HPF_6 salt **24C8⊂3•**HPF_6 was isolated as a colorless powder (52 mg, 0.06 mmol, 55%). **^{1}H NMR** (600 MHz, CD_3CN) δ_H 3.40 (s, 32H, 16 × C^7H_2), 4.76 (t, J = 6.4, 4H, 2 × C^6H_2), 7.93 (s, 2H, NH_2), 8.11 (s, 2H, 2 × C^1H), 8.20 (s, 4H, 4 × C^4H). **^{13}C NMR** (151 MHz, CD_3CN) δ_C 52.3 (2 × C^6), 71.1 (16 × C^7), 124.3 (q, J = 272.4, 4 × C^3), 124.4 (spt, J = 4.1, 2 × C^1), 132.1 (q, J = 33.6, 4 × C^2), 132.6 (q, J = 2.8, 4 × C^4), 135.6 (2 × C^5). **HR-MS** (ESI, positive ion mode) – m/z calculated for $[C_{34}H_{43}F_{12}NO_8+H]^+$ = 822.2870. Found 822.2839.

Bis(3,5-bis(trifluoromethyl)benzyl)amine{24-crown-8}, 24C8⊂3

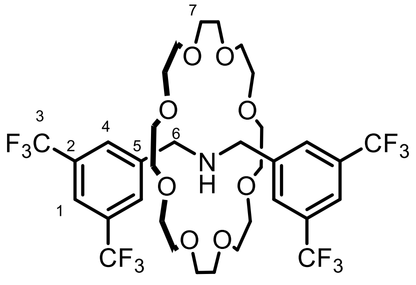

Synthesized according to general procedure C using **24C8⊂3**•HPF_6 (3 mg, 4 µM, 1.0 equiv.). **^{1}H NMR** (600 MHz, CD_3CN) δ_H 3.39 (s, 32H, 16 × C^7H_2), 4.32 (d, *J* = 7.6, 4H, 2 × C^6H_2), 7.77 (s, 2H, 2 × C^1H), 8.23 (s, 4H, 4 × C^4H). **^{13}C NMR** (151 MHz, CD_3CN) δ_C 52.8 (2 × C^6), 71.5 (16 × C^7), 120.1 (spt, *J* = 4.2, 2 × C^1), 125.2 (q, *J* = 272.1, 4 × C^3), 130.4 (q, *J* = 32.4, 4 × C^2), 131.7 (2 × C^4), 147.9 (2 × C^5).

Bis(3,5-bis(trifluoromethyl)benzyl)ammonium{2,3,14,15-dibenzo-24-crown-8} hexafluorophosphate, DB24C8⊂3•HPF$_6$

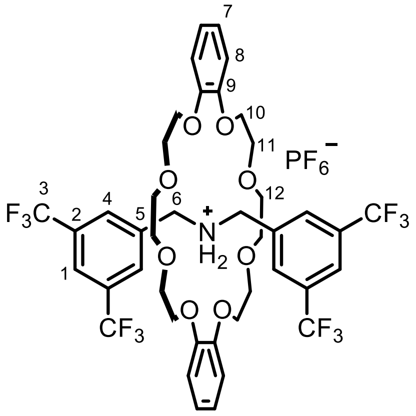

Synthesized according to general procedure A using 3,5-bis(trifluoromethyl)benzylamine (50 mg, 0.21 mmol, 2.0 equiv.), dibenzo-24-crown-8 (47 mg, 0.11 mmol, 1.0 equiv.) and 3,5-bis(trifluoromethyl)benzyl bromide (32 mg, 0.11 mmol, 1.0 equiv.). The reaction was performed at room temperature for 16 hours. The pure HBr salt was subjected to general procedure B and the title rotaxane HPF_6 salt **DB24C3⊂3**•HPF_6 was isolated as a colorless powder (55 mg, 0.06 mmol, 53%). **^{1}H NMR** (600 MHz, CD_3CN) δ_H 3.67 (s, 8H, 4 × $C^{12}H_2$), 3.78 (t, *J* = 3.4, 8H, 4 × $C^{11}H_2$), 3.95 (t, *J* = 3.4, 8H, 4 × $C^{10}H_2$), 4.89 (s, 4H, 2 × C^6H_2), 6.62 (dd, *J* = 3.6, 6.0, 4H, 4 × C^8H), 6.70 (dd, *J* = 3.6, 6.0, 4H, 4 × C^7H), 7.70 (s, 2H, 2 × C^1H), 8.02 (s, 4H, 4 × C^4H). **^{13}C NMR** (151 MHz, CD_3CN) δ_C 52.3 (2 × C^6), 68.5 (4 × C^{10}), 71.0 (4 × C^{11}), 71.7 (4 × C^{12}), 113.8 (4 × C^8), 123.1 (4 × C^7), 124.6 (2 × C^1), 125.1 (q, *J* = 273.2, 4 × C^3), 132.0 (4 × C^4), 133.0 (q, *J* = 33.8, 4 × C^2), 136.9 (2 × C^5), 148.4 (4 × C^9). **HR-MS** (ESI, positive ion mode) – *m/z* calculated for $[C_{42}H_{43}F_{12}NO_8+H]^+$ = 918.2870. Found 918.2840.

Bis(3,5-bis(trifluoromethyl)benzyl)amine{2,3,14,15-dibenzo-24-crown-8}, DB24C8⊂3

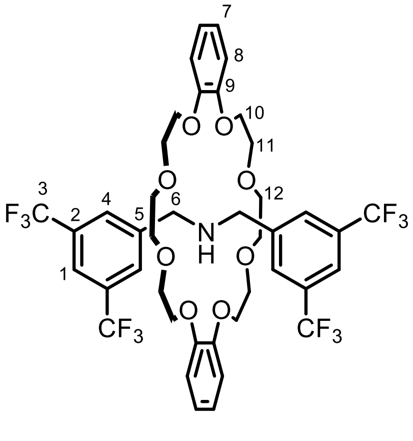

Synthesized according to general procedure C using **DB24C8⊂3**•HPF_6 (4 mg, 4 μM, 1.0 equiv.). **^{1}H NMR** (600 MHz, CD_3CN) δ_H 3.25 (s, 8H, 4 × $C^{12}H_2$), 3.61 (t, J = 4.0, 8H, 4 × $C^{11}H_2$), 4.00 (t, J = 4.0, 8H, 4 × $C^{10}H_2$), 4.10 (d, J = 7.1, 4H, 2 × C^6H_2), 6.75–6.79 (m, 8H, 4 × C^7H, 4 × C^8H), 7.62 (s, 2H, 2 × C^1H), 8.20 (s, 4H, 4 × C^4H). **^{13}C NMR** (151 MHz, CD_3CN) δ_C 53.7 (2 × C^6), 68.9 (4 × C^{10}), 70.3 (4 × C^{11}), 71.3 (4 × C^{12}), 112.7 (4 × C^8), 120.1 (spt, J = 4.0, 2 × C^1), 121.4 (4 × C^7), 125.0 (q, J = 274.3, 4 × C^3), 130.5 (q, J = 32.6, 4 × C^2), 130.8 (4 × C^4), 146.8 (2 × C^5), 149.0 (4 × C^9).

Bis(3,5-bis(trifluoromethyl)benzyl)ammonium{21-crown-7} hexafluorophosphate, 21C7⊂3•HPF$_6$

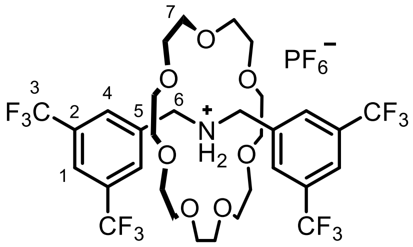

Synthesized according to general procedure A using 3,5-bis(trifluoromethyl)benzylamine (55 mg, 0.22 mmol, 2.0 equiv.), 21-crown-7 **S1** (34 mg, 0.11 mmol, 1.0 equiv.) and 3,5-bis(trifluoromethyl)benzyl bromide (35 mg, 0.11 mmol, 1.0 equiv.). The reaction was performed at room temperature for 16 hours. The pure HBr salt was subjected to general procedure B and the title rotaxane HPF_6 salt **21C7⊂3**•HPF_6 was isolated as a colorless powder (37 mg, 0.05 mmol, 47%). **^{1}H NMR** (600 MHz, CD_3CN) δ_H 3.50 (s, 28H, 14 × C^7H_2), 4.82 (s, 4H, 2 × C^6H_2), 8.00 (s, 2H, NH_2), 8.06 (s, 4H, 4 × C^4H), 8.13 (s, 2H, 2 × C^1H). **^{13}C NMR** (151 MHz, CD_3CN) δ_C 51.3 (2 × C^6), 71.7 (16 × C^7), 124.3 (q, J = 272.1, 4 × C^3), 124.6 (spt, J = 4.0, 2 × C^1), 132.2 (q, J = 2.5, 4 × C^4), 132.4 (q, J = 34.2, 4 × C^2), 136.0 (2 × C^5). **HR-MS** (ESI, positive ion mode) – *m/z* calculated for $[C_{32}H_{39}F_{12}NO_7+H]^+$ = 778.2608. Found 778.2580.

Bis(3,5-bis(trifluoromethyl)benzyl)amine{21-crown-7}, 21C7⊂3

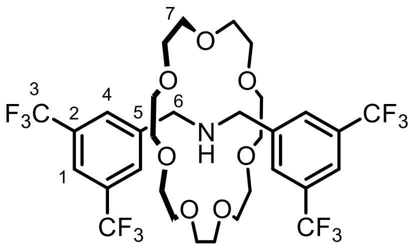

Synthesized according to general procedure C using **21C7⊂3**•HPF_6 (3 mg, 4 μM, 1.0 equiv.). **^{1}H NMR** (600 MHz, CD_3CN) δ_H 3.39 (s, 28H, 14 × C^7H_2), 4.32 (d, J = 7.2, 4H, 2 × C^6H_2), 7.77 (s, 2H, 2 × C^1H), 8.23 (s, 4H, 4 × C^4H). **^{13}C NMR**

(151 MHz, CD_3CN) δ_C 52.8 (2 × C^6), 71.5 (14 × C^7), 120.1 (spt, J = 4.0, 2 × C^1), 125.2 (q, J = 271.8, 4 × C^3), 130.6 (q, J = 32.5, 4 × C^2), 131.7 (q, J = 2.3, 4 × C^4), 147.9 (2 × C^5).

N-(3,5-bis(trifluoromethyl)benzyl)-N-(3,5-bis(*tert*-butyl)benzyl)ammonium{24-crown-8} hexafluorophosphate, 24C8⊂4•HPF$_6$

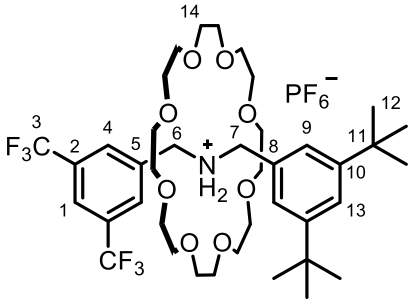

Synthesized according to general procedure A using 3,5-bis(trifluoromethyl)benzylamine (50 mg, 0.21 mmol, 2.0 equiv.), 24-crown-8 (30 mg, 0.11 mmol, 1.0 equiv.) and 3,5-bis(*tert*-butyl)benzyl bromide (30 mg, 0.11 mmol, 1.0 equiv.). The reaction was performed at room temperature for 16 hours. The pure HBr salt was subjected to general procedure B and the title rotaxane HPF_6 salt **24C8⊂4**•HPF_6 was isolated as a colorless powder (30 mg, 0.027 mmol, 35%). **^{1}H NMR** (600 MHz, CD_3CN) δ_H 1.33 (s, 18H, 6 × $C^{12}H_3$), 3.33–3.38 (m, 16H, 16 × $OC^{14}H^A$), 3.40–3.44 (m, 16H, 16 × $OC^{14}H^B$), 4.47 (t, J = 6.6, 2H, C^7H_2), 4.74 (t, J = 6.9, 2H, C^6H_2), 7.37 (d, J = 1.6, 2H, 2 × C^9H), 7.56 (d, J = 1.8, 1H, $C^{13}H$), 7.73 (s, 2H, 2 × NH), 8.07 (s, 1H, C^1H), 8.24 (s, 2H, 2 × C^4H). **^{13}C NMR** (151 MHz, CD_3CN) δ_C 31.7 (6 × C^{12}), 34.7 (2 × C^{11}), 51.9 (C^6), 54.5 (C^7), 71.1 (16 × C^{14}), 123.8 (spt, J = 3.9, C^1), 124.0 (C^{13}), 124.4 (q, J = 272.3, 2 × C^3), 125.3 (2 × C^9), 130.4 (C^8), 130.8 (q, J = 33.3, 2 × C^2), 131.8 (q, J = 3.1, 2 × C^4), 135.3 (C^5), 151.3 (2 × C^{10}). **HR-MS** (ESI, positive ion mode) – *m/z* calculated for $[C_{40}H_{61}NO_8+H]^+$ = 798.4380. Found 798.4348.

N-(3,5-bis(trifluoromethyl)benzyl)-N-(3,5-bis(*tert*-butyl)benzyl)amine{24-crown-8}, 24C8⊂4

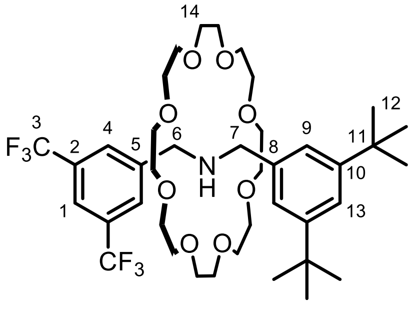

Synthesized according to general procedure C using **24C8⊂4**•HPF_6 (3 mg, 4 µM, 1.0 equiv.). **^{1}H NMR** (600 MHz, CD_3CN) δ_H 1.31 (s, 18H, 6 × $C^{12}H_3$), 3.20–3.26 (m, 16H, 16 × $OC^{14}H^A$), 3.37–3.43 (m, 16H, 16 × $OC^{14}H^B$), 3.89 (s, 2H, C^7H_2), 4.24 (s, 2H, C^6H_2), 7.28 (s, 2H, 2 × C^9H), 7.34 (s, 1H, $C^{13}H$), 7.72 (s, 1H, C^1H), 8.48 (s, 2H, 2 × C^4H). **^{13}C NMR** (151 MHz, CD_3CN) δ_C 31.9 (6 × C^{12}), 34.2 (2 × C^{11}), 53.8 (C^6), 56.9 (C^7), 71.1 (16 × C^{14}), 119.7 (C^1), 121.7 (C^{13}), 124.7 (2 × C^9), 125.3 (q, J = 273.1, 2 × C^3), 130.0 (q, J = 33.0, 2 × C^2), 132.7 (q, J = 2.5, 2 × C^4), 141.6 (C^8), 147.0 (C^5), 151.1 (2 × C^{10}).

***N*-(3,5-bis(trifluoromethyl)benzyl)-*N*-(3,5-bis(*tert*-butyl)-4-methoxybenzyl)ammonium{24-crown-8} hexafluorophosphate, 24C8⊂5•HPF$_6$**

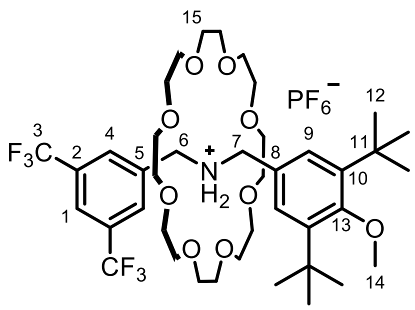

Synthesized according to general procedure A using 3,5-bis(trifluoromethyl)benzylamine (20 mg, 82 µmol, 1.0 equiv.), 24-crown-8 (29 mg, 82 µmol, 1.0 equiv.) and 3,5-bis(*tert*-butyl)-4-methoxybenzyl bromide **S3** (26 mg, 82 µmol, 1.0 equiv.). The reaction was performed at room temperature for 16 hours. The pure HBr salt was subjected to general procedure B and the title rotaxane HPF$_6$ salt **24C8⊂5**•HPF$_6$ was isolated as a colorless powder (13 mg, 16 µmol, 19%). **^{1}H NMR** (600 MHz, CD$_3$CN) δ_H 1.42 (s, 18H, 6 × C^{12}*H*$_3$), 3.35–3.39 (m, 16H, 16 × C^{15}*H*^{A}), 3.40–3.44 (m, 16H, 16 × C^{15}*H*^{B}), 3.66 (s, 3H, C^{14}*H*$_3$), 4.47 (t, *J* = 6.6, 2H, C^7*H*$_2$), 4.70 (t, *J* = 6.6, 2H, C^6*H*$_2$), 7.43 (s, 2H, 2 × C^9*H*), 7.66 (s, 2H, N*H*$_2$), 8.06 (s, 1H, C^1*H*), 8.21 (s, 2H, 2 × C^4*H*). **^{13}C NMR** (151 MHz, CD$_3$CN) δ_C 32.3 (6 × *C*^{12}), 36.5 (2 × *C*^{11}), 51.7 (*C*^{6}), 54.1 (*C*^{7}), 65.5 (*C*^{14}), 71.1 (16 × *C*^{15}), 123.8 (spt, *J* = 3.9, *C*^{1}), 124.4 (q, *J* = 272.3, 2 × *C*^{3}), 126.6 (*C*^{8}), 130.9 (2 × *C*^{9}), 131.8 (q, *J* = 33.5, 2 × *C*^{2}), 132.5 (q, *J* = 3.0, 2 × *C*^{4}), 136.3 (*C*^{5}), 144.8 (2 × *C*^{10}), 161.8 (*C*^{13}). **HR-MS** (ESI, positive ion mode) – *m/z* calculated for $[C_{41}H_{63}F_6NO_9+H]^+$ = 828.4480. Found 828.4489.

***N*-(3,5-bis(trifluoromethyl)benzyl)-*N*-(3,5-bis(*tert*-butyl)-4-methoxybenzyl)amine{24-crown-8}, 24C8⊂5**

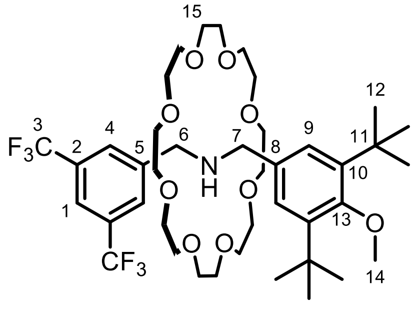

Synthesized according to general procedure C using **24C8⊂5**•HPF$_6$ (3 mg, 4 µM, 1.0 equiv.). **^{1}H NMR** (600 MHz, CD$_3$CN) δ_H 1.41 (s, 18H, 6 × C^{12}*H*$_3$), 3.22–3.26 (m, 16H, 16 × C^{15}*H*^{A}), 3.38–3.42 (m, 16H, 16 × C^{15}*H*^{B}), 3.63 (s, 3H, C^{14}*H*$_3$), 3.82 (d, *J* = 6.8, 2H, C^7*H*$_2$), 4.21 (d, *J* = 7.2, 2H, C^6*H*$_2$), 7.31 (s, 2H, 2 × C^9*H*), 7.71 (s, 1H, C^1*H*), 8.45 (s, 2H, 2 × C^4*H*). **^{13}C NMR** (151 MHz, CD$_3$CN) δ_C 32.5 (6 × *C*^{12}), 36.3 (2 × *C*^{11}), 53.7 (*C*^{6}), 56.4 (*C*^{7}), 65.1 (*C*^{14}), 71.1 (16 × *C*^{15}), 119.7 (spt, *J* = 4.0, *C*^{1}), 125.3 (q, *J* = 271.7, 2 × *C*^{3}), 129.1 (2 × *C*^{9}), 130.0 (q, *J* = 32.5, 2 × *C*^{2}), 132.4 (q, *J* = 2.9, 2 × *C*^{4}), 136.6 (*C*^{8}), 143.5 (2 × *C*^{10}), 147.1 (*C*^{5}), 159.3 (*C*^{13}).

Bis(3,5-bis(*tert*-butyl)benzyl)ammonium{24-crown-8} hexafluorophosphate, 24C8⊂2•HPF$_6$

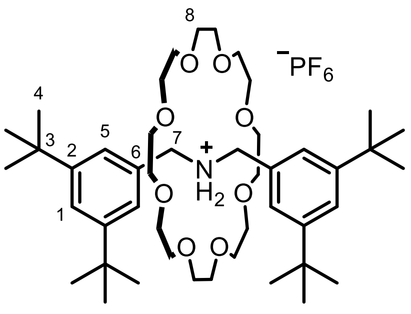

Synthesized according to general procedure A using 3,5-bis(*tert*-butyl)benzylamine **S5** (18 mg, 82 µmol, 1.0 equiv.), 24-crown-8 (29 mg, 82 µmol, 1.0 equiv.) and 3,5-bis(*tert*-butyl)benzyl bromide (23 mg, 82 µmol, 1.0 equiv.). The reaction was performed at room temperature for 72 hours. The pure HBr salt was subject to general procedure B and the title rotaxane HPF$_6$ salt **24C8⊂2**•HPF$_6$ was isolated as a colorless powder (9 mg, 12 µmol, 14%). **^{1}H NMR** (600 MHz, CD$_3$CN) δ_H 1.32 (s, 36H, 12 × C^4*H*$_3$), 3.38 (s, 32H, 16 × C^8*H*$_2$), 4.49 (t, *J* = 6.9, 4H, 2 × C^7*H*$_2$), 7.41 (d, *J* = 1.7, 4H, 4 × C^5*H*), 7.50 (t, *J* = 1.7, 2H, 2 × C^1*H*), 7.60 (s, 2H, N*H*$_2$). **^{13}C NMR** (151 MHz, CD$_3$CN) δ_C 31.7 (12 × *C*^{4}), 35.6 (4 × *C*^{3}), 54.1 (2 × *C*^{7}), 71.1 (16 × *C*^{8}), 124.3 (2 × *C*^{1}), 126.3 (4 × *C*^{5}), 132.2 (2 × *C*^{6}), 151.8 (4 × *C*^{2}). **HR-MS** (ESI, positive ion mode) – *m/z* calculated for $[C_{46}H_{79}NO_8+H]^+$ = 774.5884. Found 774.5853.

Bis(3,5-bis(*tert*-butyl)benzyl)amine{24-crown-8}, 24C8⊂2

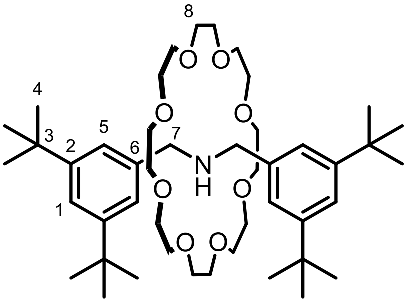

Synthesized according to general procedure C using **24C8⊂2**•HPF$_6$ (3 mg, 4 µM, 1.0 equiv.). **^{1}H NMR** (600 MHz, CD$_3$CN) δ_H 1.30 (s, 36H, 12 × C^4*H*$_3$), 3.38 (s, 32H, 16 × C^8*H*$_2$), 3.98 (s, 4H, 2 × C^7*H*$_2$), 7.28 (s, 2H, 2 × C^1*H*), 7.41 (s, 4H, 4 × C^5*H*). **^{13}C NMR** (151 MHz, CD$_3$CN) δ_C 32.0 (12 × *C*^{4}), 35.5 (4 × *C*^{3}), 56.4 (2 × *C*^{7}), 71.0 (16 × *C*^{8}), 121.0 (2 × *C*^{1}), 125.4 (4 × *C*^{5}), 142.4 (2 × *C*^{6}), 150.5 (4 × *C*^{2}).

***N*-3,5-bis(trifluoromethyl)benzyl-3-oxo-3-(3,5-bis(trifluoromethyl)phenylamino)propan-1-ammonium{2,3,14,15-dibenzo-24-crown-8} hexafluorophosphate, DB24C8⊂5•HPF$_6$**

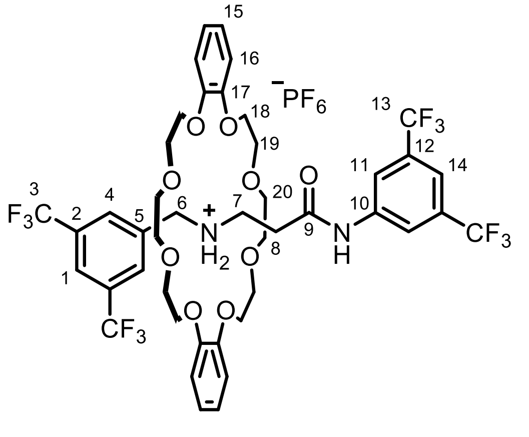

Synthesized according to general procedure A using 3,5-bis(trifluoromethyl)benzylamine (100 mg, 0.41 mmol, 2.0 equiv.), dibenzo-24-crown-8 (184 mg, 0.21 mmol, 1.0 equiv.) and 3,5-bis(trifluoromethyl)phenyl acrylamide **7** (58 mg, 0.21 mmol, 1.0 equiv.). The reaction was performed at room temperature for 16 hours. The pure HBr salt was subjected to general procedure B and the title rotaxane HPF$_6$ salt **DB24C8⊂8**•HPF$_6$ was isolated as a foamy colorless powder (160 mg, 0.11 mmol, 55%). **^{1}H NMR** (600 MHz, CD$_3$CN) δ_H 2.98 (t, *J* = 6.8, 2H, C^8*H*$_2$), 3.72–

3.81 (m, 10H, 4 × $C^{19}H^{A}$, 4 × $C^{20}H^{A}$, $C^{7}H_2$), 3.86–3.90 (m, 4H, 4 × $C^{20}H^{B}$), 3.91–3.96 (m, 4H, 4 × $C^{18}H^{A}$), 4.01–4.07 (m, 8H, 4 × $C^{18}H^{B}$, 4 × $C^{19}H^{B}$), 4.96 (t, J = 6.9, 2H, $C^{6}H_2$), 6.60–6.63 (m, 4H, 4 × $C^{16}H$), 6.65–6.68 (m, 4H, 4 × $C^{15}H$), 7.30 (s, 1H, $C^{1}H$), 7.65 (s, 2H, NH_2), 7.71 (s, 1H, $C^{14}H$), 7.92 (s, 2H, 2 × $C^{4}H$), 8.12 (s, 2H, 2 × $C^{11}H$), 9.28 (s, 1H, C(O)NH). **^{13}C NMR** (151 MHz, CD_3CN) δ_C 33.6 (C^{8}), 45.9 (C^{7}), 52.1 (C^{6}), 68.5 (4 × C^{18}), 71.2 (4 × C^{19}), 71.8 (4 × C^{20}), 112.8 (4 × C^{16}), 118.1 (C^{14}), 120.2 (2 × C^{11}), 122.0 (4 × C^{15}), 122.5 (C^{1}), 124.1 (q, J = 272.8, 2 × C^{3}), 124.4 (q, J = 272.2, 2 × C^{13}), 131.1 (q, J = 33.0, 2 × C^{2}), 131.3 (2 × C^{4}), 132.6 (q, J = 33.3, 2 × C^{2}), 136.1 (C^{5}) 141.1 (C^{10}), 147.6 (4 × C^{17}), 169.6 (C^{9}). **HR-MS** (ESI, positive ion mode) – *m/z* calculated for $[C_{44}H_{46}F_{12}NO_9+H]^+$ = 975.3084. Found 975.3088.

N-3,5-bis(trifluoromethyl)benzyl-3-oxo-3-(3,5-bis(trifluoromethyl)phenylamino)propan-1-amine{2,3,14,15-dibenzo-24-crown-8}, DB24C8⊂8

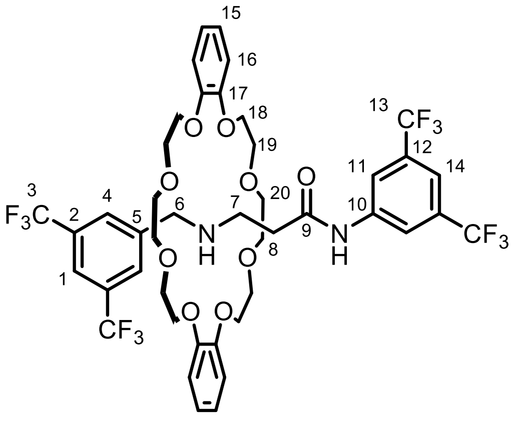

Synthesized according to general procedure C using **DB24C8⊂8**•HPF_6 (4 mg, 4 µM, 1.0 equiv.). **^{1}H NMR** (600 MHz, CD_3CN) δ_H 2.41 (t, J = 6.1, 2H, $C^{8}H_2$), 2.98 (t, J = 6.1, 2H, $C^{7}H_2$), 3.30–3.35 (m, 4H, 4 × $C^{20}H^{A}$), 3.59–3.63 (m, 4H, 4 × $C^{19}H^{A}$), 3.64–3.68 (m, 4H, 4 × $C^{20}H^{B}$), 3.75–3.79 (m, 4H, 4 × $C^{19}H^{B}$), 3.89–3.94 (m, 4H, 4 × $C^{18}H^{A}$), 4.14–4.18 (m, 4H, 4 × $C^{18}H^{B}$), 4.29 (s, 2H, $C^{6}H_2$), 6.76–6.81 (m, 8H, 4 × $C^{15}H$, 4 × $C^{16}H$), 7.08 (s, 1H, $C^{1}H$), 7.35 (s, 1H, $C^{14}H$), 7.96 (s, 2H, 2 × $C^{4}H$), 8.31 (s, 2H, 2 × $C^{11}H$). **^{13}C NMR** (151 MHz, CD_3CN) δ_C 41.4 (C^{8}), 49.6 (C^{7}), 53.9 (C^{6}), 69.0 (4 × C^{18}), 70.5 (4 × C^{19}), 71.7 (4 × C^{20}), 109.9 (2 × C^{1}), 112.9 (4 × C^{16}), 119.3 (C^{14}), 121.6 (4 × C^{15}), 123.4 (C^{5}), 124.0 (2 × C^{4}), 125.1 (q, J = 272.0, 2 × C^{3}), 125.7 (q, J = 271.4, 2 × C^{13}), 129.6 (q, J = 32.3, 2 × C^{2}), 130.9 (q, J = 31.5, 2 × C^{12}), 131.1 (2 × C^{11}), 146.7 (C^{10}), 148.5 (4 × C^{17}), 177.9 (C^{9}).

Bis(3,5-dimethylbenzyl)ammonium{21-crown-7} hexafluorophosphate, 21C7⊂6•HPF_6

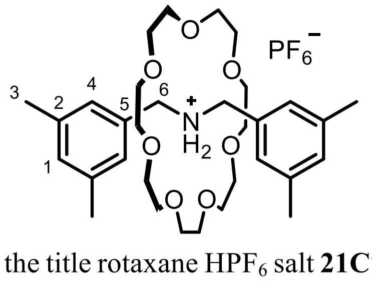

Synthesized according to general procedure A using 3,5-dimethylbenzylamine (50 mg, 0.37 mmol, 2.0 equiv.), 21-crown-7 **S1** (50 mg, 0.17 mmol, 1.0 equiv.) and 3,5-dimethylbenzyl bromide (37 mg, 0.19 mmol, 1.0 equiv.). The reaction was performed at −10 °C for 72 hours. The pure HBr salt was subjected to general procedure B and the title rotaxane HPF_6 salt **21C7⊂6**•HPF_6 was isolated as a colorless powder (12 mg, 0.02 mmol, 10%). **^{1}H NMR** (600 MHz, CD_3CN) δ_H 2.32 (s, 12H, 4 × $C^{3}H_3$), 3.48 (s, 28H, 14 × $C^{7}H_2$), 4.49 (t, J = 6.8, 4H, 2 × $C^{6}H_2$), 7.05 (s, 2H, 2 × $C^{1}H$), 7.11 (s, 4H, 4 × $C^{4}H$), 7.56 (s, 2H, NH_2). **^{13}C NMR** (151 MHz,

CD_3CN) δ_C 21.3 (4 × C^3), 52.0 (2 × C^6), 71.8 (14 × C^7), 129.0 (4 × C^4), 131.2 (2 × C^1), 133.8 (2 × C^5), 139.1 (4 × C^2). **HR-MS** (ESI, positive ion mode) – *m/z* calculated for $[C_{32}H_{51}NO_7+H]^+$ = 562.3738. Found 562.3881.

Bis(3,5-dimethylbenzyl)amine{21-crown-7}, 21C7⊂6

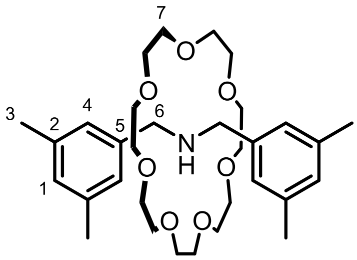

Synthesized according to general procedure C using **21C7⊂6**•HPF_6 (2 mg, 4 μM, 1.0 equiv.). **^{1}H NMR** (600 MHz, CD_3CN) δ_H 2.25 (s, 12H, 4 × C^3H_3), 3.46 (s, 28H, 14 × C^7H_2), 4.06 (s, 4H, 2 × C^6H_2), 6.76 (s, 2H, 2 × C^1H), 7.21 (s, 4H, 4 × C^4H). **^{13}C NMR** (151 MHz, CD_3CN) δ_C 21.4 (4 × C^3), 53.3 (2 × C^6), 71.1 (14 × C^7), 127.4 (2 × C^1), 128.8 (4 × C^4), 137.1 (2 × C^5), 144.9 (4 × C^2).

2.0 pK_aH$^+$ Measurements

The measurement of pK_aH$^+$ values for the rotaxanes was carried out using ^{1}H NMR spectroscopy by Lambert's previously reported method.[7] Appropriate reference bases were chosen for each rotaxane, and the equilibrium constant corresponding to the rotaxane superbase and the reference base was used to extrapolate the rotaxane pK_aH$^+$.

Consider a solution consisting of the HPF_6 salt of a rotaxane (superbase•HA) and a reference base (std). At equilibrium, the rotaxane HPF_6 salt will, to a certain extent, be deprotonated by the reference base to give the neutral rotaxane (superbase) and the reference base HPF_6 salt (std•HA) such that –

$$K_a = \frac{\left(\frac{\delta_{obs}-\delta_{std}}{\delta_{std\bullet HA}-\delta_{obs}}\right)}{r\left(\frac{\delta_{std\bullet HA}-\delta_{std}}{\delta_{obs}-\delta_{std}}\right)-1} \quad (1)$$

K_a – equilibrium constant   δ = chemical shift (ppm)   r = molar ratio of rotaxane / standard

Therefore, the equilibrium constant, K_a can be determined for a solution containing a rotaxane superbase and reference base when the reference base protons are in fast exchange on the NMR timescale. δ_{std} and $\delta_{std\bullet HA}$ were determined by recording ^{1}H NMR spectra of pre-prepared solutions of the reference base and its HPF_6 salt, using signals whose chemical shifts were independent of concentration. The corresponding observed chemical shift of that proton, δ_{obs} in the rotaxane superbase / standard mixture was then used to determine K_a.

It follows that –

$$pK_aH^+{}_{superbase} = pK_aH^+{}_{std} + pK_a = pK_aH^+{}_{std} - \log_{10}(K_a) \quad (2)$$

Therefore, rotaxane pK_aH$^+$ can be determined using known pK_aH$^+$ values of reference bases and K_a values determined using equation (1).

Similarly, K_a can also be determined when the rotaxanes are in fast exchange on the NMR timescale such that –

$$K_a = \frac{\left(\frac{\delta_{superbase\bullet HA}-\delta_{obs}}{\delta_{obs}-\delta_{superbase}}\right)}{\frac{1}{r}\left(\frac{\delta_{superbase\bullet HA}-\delta_{superbase}}{\delta_{superbase\bullet HA}-\delta_{obs}}\right)-1} \quad (3)$$

Therefore, rotaxane pK_aH$^+$ can be determined using known pK_aH$^+$ values of reference bases and K_a values determined using equation (3).

Finally, as –

$$K_a = \frac{\left(\frac{[superbase]}{[superbase\cdot HA]}\right)}{\frac{1}{r}\left(\frac{[superbase]_{Total}}{[superbase]}\right)-1} \qquad (4)$$

Therefore, if the rotaxane superbase and it's conjugate acid are in slow exchange on the NMR timescale, then –

$$K_a = \frac{\left(\frac{Int_{superbase}}{Int_{superbase\cdot HA}}\right)}{\frac{1}{r}\left(\frac{Int_{superbase}+Int_{superbase\cdot HA}}{Int_{superbase}}\right)-1} \qquad (5)$$

Int = relative integration

Equations (1)-(5) were used to determine rotaxane pK_aH$^+$ depending on the proton-exchange timescales of the rotaxanes and/or reference bases. Additionally, equation (1) and equation (3) or (5) were used on the same data to ensure self-consistency.

pK_aH$^+$ measurement data for the measured compounds are detailed below. All ^{1}H NMR spectra were recorded in anhydrous CD_3CN (600 MHz).

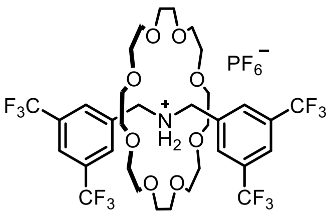

24C8□3 •HPF$_6$

**Equiv.**	**pK_aH$^+$**
0.32	20.55
0.6	20.61
0.93	20.49
0.78	20.57
1.06	20.46
**Mean**	**20.54**
**Error**	0.08

Ref. base: 1,4-diaminobutane (pK_aH$^+$ = 20.12)

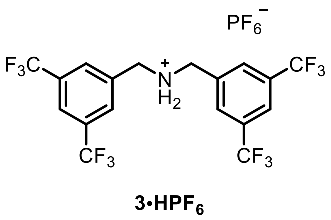

3•HPF$_6$

**Equiv.**	**pK_aH$^+$**
0.24	12.27
0.66	12.11
0.8	12.01
1.5	11.63
1.8	11.9
**Mean**	**11.98**
**Error**	0.32

Ref. base: Pyridine (pK_aH$^+$ = 12.53)

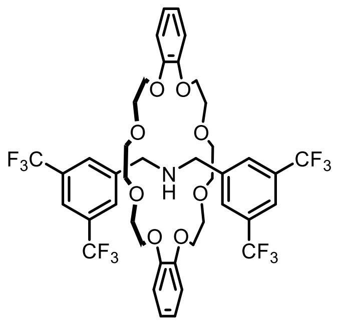

DB24C8□3 •HPF$_6$

Equiv.	pK_aH$^+$
0.15	20.37
0.43	20.33
0.46	20.28
0.65	20.27
1.02	20.24
**Mean**	**20.30**
**Error**	0.07

Ref. base: 1,4-diaminobutane (pK_aH$^+$ = 20.12)

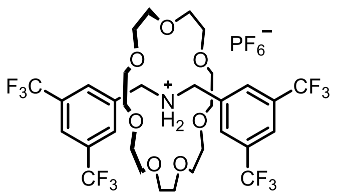

21C7□3 •HPF$_6$

Equiv.	pK_aH$^+$
0.43	25.01
0.97	24.88
1.02	24.76
1.45	24.87
2.07	24.82
**Mean**	**24.87**
**Error**	0.13

Ref. base: mTBD (pK_aH$^+$ = 25.47)

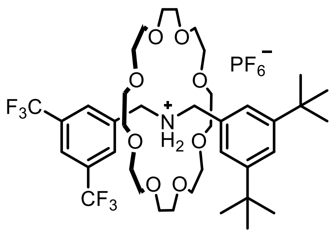

24C8□4 •HPF$_6$

Equiv.	pK_aH$^+$
0.43	23.1
0.76	23.03
1.1	22.91
1.53	22.59
1.66	22.63
**Mean**	**22.85**
**Error**	0.26

Ref. base: TMG (pK_aH$^+$ = 23.35)

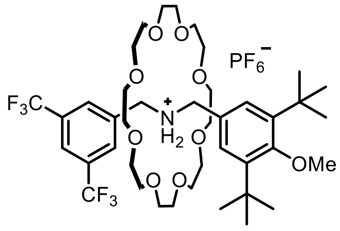

24C8□5 •HPF$_6$

Equiv.	pK_aH$^+$
0.29	23.13
0.47	23.17
0.69	23.18
0.8	23.16
0.92	23.16
**Mean**	**23.16**
**Error**	0.03

Ref. base: TMG (pK_aH$^+$ = 23.35)

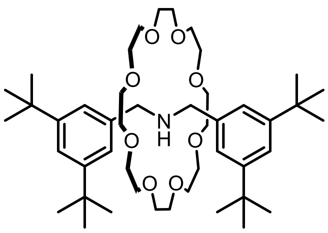

24C8□2 •HPF$_6$

Ref. base: P_1-*t*-Bu (pK_aH$^+$ = 27.01)

**Equiv.**	**pK_aH$^+$**
0.28	26.6
0.59	26.22
0.9	26.21
1.1	26.15
1.27	26.21
**Mean**	**26.28**
**Error**	0.23

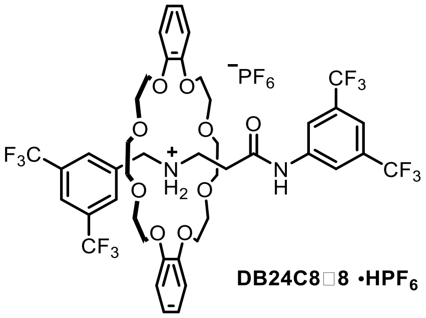

DB24C8□8 •HPF$_6$

Ref. base: 1,4-diaminobutane (pK_aH$^+$ = 20.12)

**Equiv.**	**pK_aH$^+$**
0.25	20.43
0.57	20.61
0.84	20.62
1.17	21.01
**Mean**	**20.67**
**Error**	0.29

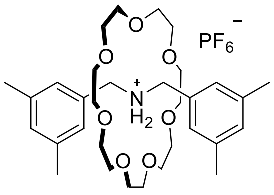

21C7□6•HPF $_6$

Ref. base: P_2-Et (pK_aH$^+$ = 32.9)

**Equiv.**	**pK_aH$^+$**
0.81	32.01
1.23	31.52
0.91	32.67
1.18	32.62
**Mean**	**32.21**
**Error**	0.58

2.1 pK_aH$^+$ Measurement Using More than one Reference Base

Rotaxane **21C7⊂3**•HPF$_6$ was measured with DBU as a reference base in order to verify that pK_aH$^+$ measurements are reliable regardless of reference base used. With mTBD, the pK_aH$^+$ of **21C7⊂3**•HPF$_6$ was measured to be 24.87 ± 0.13 (see previous section). Using DBU:

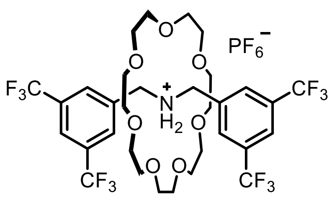

21C7□3•HPF $_6$

Equiv.	**pK_aH$^+$**
1.27	24.83
1.27	25.24
2.22	25.05
2.22	25.06
**Mean**	**25.04**
**Error**	0.22

Ref. base: DBU (pK_aH$^+$ = 24.31)

This result is within error of the value found using mTBD.

2.2 pK_aH$^+$ Measurements in Alternative Solvents

The formation of hydrogen bonds between the crown ether macrocycle and protonated amine plays a key role in rotaxane superbasicity, therefore we hypothesized that a coordinating solvent such as acetonitrile will disrupt this, lowering the pK_aH$^+$ of the rotaxanes relative to other superbases that do not rely on supramolecular interactions. As such, rotaxane superbases may perform better in more apolar solvents such as dichloromethane or toluene. Unfortunately, pK_aH$^+$ data in apolar solvents is scarce, hence pK_aH$^+_{rel}$ will be used, which describes the difference in pK_aH$^+$ between two species in a given solvent without a definitive pK_aH$^+$ value; for example, in acetonitrile DBU has a pK_aH$^+$ of 24.31 and mTBD has a pK_aH$^+$ of 25.47, therefore the pK_aH$^+_{rel}$ of mTBD with respect to DBU is +1.16. Using the same method as used for the other pK_aH$^+$ measurements, the acid-base equilibrium between two superbases can be used to find the pK_aH$^+_{rel}$.

In CD_3CN, the pK_aH$^+$ of rotaxane **24C8⊂3•**HPF_6 was measured to be 20.54 ± 0.08 using TMG (pK_aH$^+$ = 23.35), giving a pK_aH$^+_{rel}$ of – 2.81 ± 0.08 for **24C8⊂3•**HPF_6. In CD_2Cl_2, the pK_aH$^+_{rel}$ was measured as:

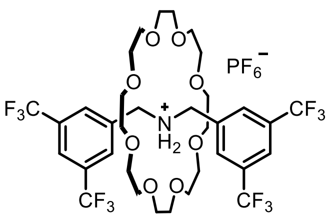

24C8□3•HPF $_6$

Equiv.	**pK_aH$^+_{rel}$**
0.47	−1.38
1.14	−1.26
0.85	−1.12
1.06	−1.17
**Mean**	**−1.2325**
**Error**	0.13

This $pK_aH^+_{rel}$ is significantly higher in CD_2Cl_2, confirming that in apolar solvents rotaxane superbases are more basic relative to other organic superbases. As such, they may find their best applications when using less polar solvents.

3.0 Protonation Versus Alkylation Studies

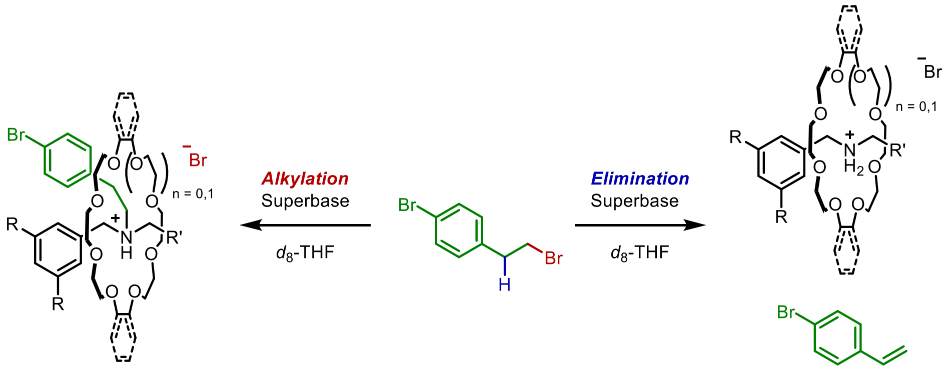

Scheme S3 – Competitive protonation versus alkylation reactions of 4-bromophenethyl bromide and rotaxane superbases **24C8⊂3**, **DB24C8⊂3**, **21C7⊂3**, **24C8⊂2, 24C8⊂8** and **21C7⊂6**. For all structures, R = CF_3 or ^{t}Bu, R' = benzylic stopper.

General Procedure – A solution of rotaxane superbase (5–9 µmol, 1.0 equiv.) in anhydrous d_8-THF (0.5 mL) was prepared in a Young's NMR tube under a dry, inert atmosphere according to general procedure C. To the rotaxane superbase solution was added 4-bromophenethyl bromide (2.0–20.0 equiv.) under a flow of nitrogen and the Young's NMR tube valve was closed. The reaction was left for the specified time at the specified temperature and monitored by ^{1}H NMR spectroscopy.

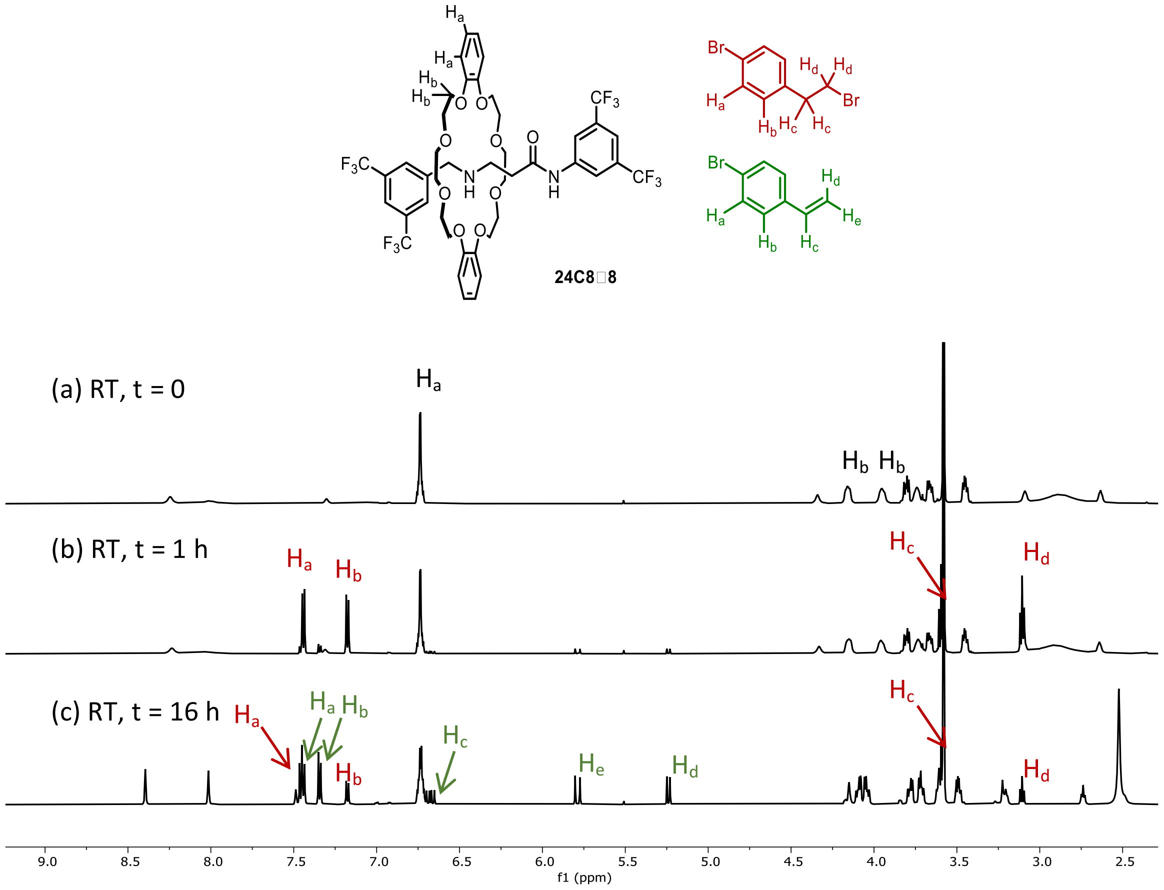

Figure S1 – Representative ^{1}H NMR spectra for protonation versus alkylation reactions of rotaxane superbases with 4-bromophenethyl bromide. (a) **24C8⊂8**; (b) **24C8⊂8** + 4-bromophenethyl bromide (2.4 equiv.) after 1 hour at room temperature; (c) **24C8⊂8** + 4-bromophenethyl bromide (2.4 equiv.) after 16 hours at room temperature. All spectra were taken in d_8-THF (600 MHz).

Table S1 – Competitive protonation versus alkylation reactions of superbases with 4-bromophenethyl bromide. *Protonated and deprotonated superbases are in fast exchange, so conversion based on equivalents of elimination product (4-bromostyrene). Half-lives were calculated based on second-order kinetics.

**Superbase**	**pK_aH$^+$ (MeCN)**	**Benzyl bromide equiv.**	**Protonation:alkylation ratio**	**Final time (temperature)**	**Superbase conversion**	**Half-life, $t_{1/2}$ (hours)**
**24C8⊂3**	20.5	2.0	>99:1	16 hours (25 °C)	7%	175.6
**DB24C8⊂3**	20.3	20.0	>99:1	16 hours (25 °C)	29%	32.6
**21C7⊂3**	24.9	4.4	>99:1	168 hours (80 °C)	3%	4072.5
**24C8⊂2**	26.3	3.2	>99:1	168 hours (80 °C)	37%	259.2
**24C8⊂8**	20.7	2.4	>99:1	16 hours (25 °C)	66%*	9.7
**21C7⊂6**	32.2	10.0	>99:1	1 hour (25 °C)	100%	0.15
DBU	24.3	1.4	87:13	168 hours (25 °C)	17%	635.3
mTBD	25.5	2.6	>99:1	120 hours (25 °C)	45%	115.3
P_1-*t*-Bu	27	0.8	>99:1	120 hours (25 °C)	21%	418.8

4.0 Hydrolytic Decomposition Experiments

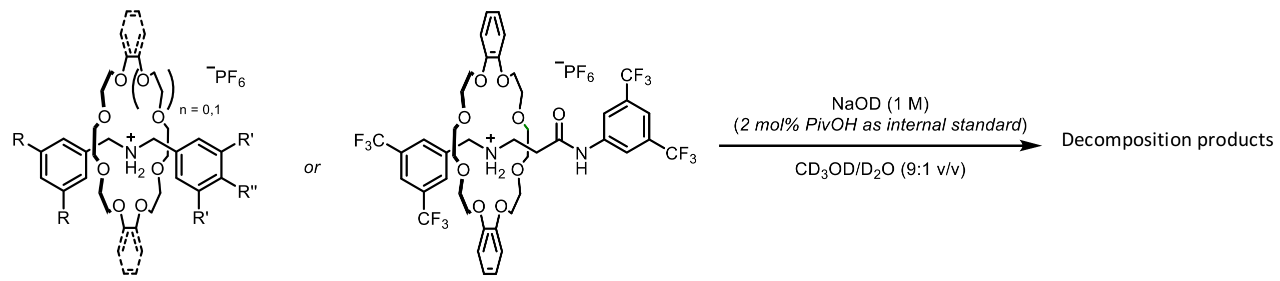

Scheme S4 – Hydrolytic decomposition of rotaxane superbase HPF_6 salts. For all structures, R,R’ = CF_3, Me or ^{t}Bu, R’’ = H or OMe.

General Procedure – A solution of rotaxane HPF_6 salt (5–9 μmol) in d_4-methanol (450 μL) was prepared in an NMR tube. To the solution was added D_2O (38 μL) and pivalic acid (~ 1 mg) and the ^{1}H NMR spectrum was recorded. Sodium deuteroxide (30 wt.% in D_2O, 12 μL) was added to the solution (making it 1 M in sodium deuteroxide). The solution was left at room temperature or heated to 80 °C and left for the specified time, and ^{1}H NMR spectra were recorded periodically.

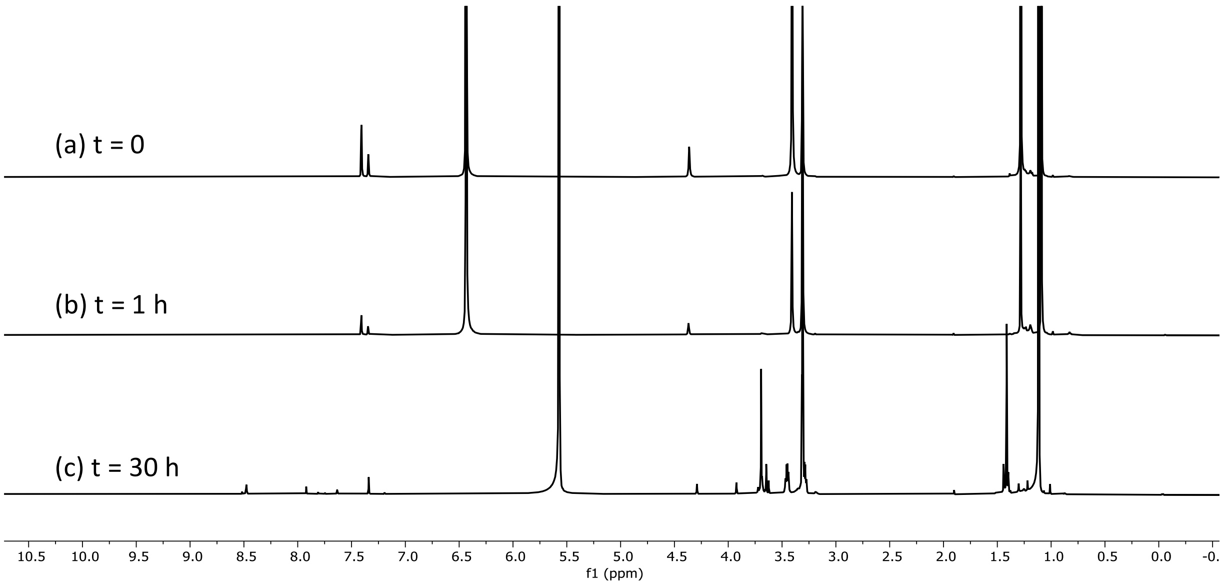

Figure S2 – Representative ^{1}H NMR spectra for hydrolytic decomposition of rotaxane HPF_6 salts. (a) **24C8⊂2**•HPF_6 in 9:1 v/v d_4-methanol:D_2O (600 MHz); (b) **24C8⊂2**•HPF_6 in 1 M NaOD in 9:1 v/v d_4-methanol:D_2O (600 MHz) for 1 hour at room temperature (600 MHz); (c) **24C8⊂2**•HPF_6 in 1 M NaOD in 9:1 v/v d_4-methanol:D_2O (600 MHz) for 30 hours at room temperature (600 MHz).

Table S2 – Hydrolytic decomposition studies of rotaxane HPF_6 salts and reference superbases in 1 M NaOD in 9:1 v/v d_4-methanol:D_2O. Half-lives were calculated based on pseudo-first-order kinetics. Concentrations were ascertained by comparing integrals with the internal standard and correlating with decomposite peaks where possible.

**Compound**	**pK_aH$^+$ (MeCN)**	**Temperature**	**Half-life**
**24C8⊂3**•HPF_6	20.5	80 °C	>7 days
**DB24C8⊂3**•HPF_6	20.3	80 °C	>7 days
**21C7⊂3**•HPF_6	24.9	25 °C	<5 minutes
**24C8⊂2**•HPF_6	26.3	25 °C	6.2 hours
**DB24C8⊂8**•HPF_6	20.7	80 °C	>7 days
**21C7⊂6**•HPF_6	32.2	80 °C	>7 days
DBU	24.3	25 °C	10.9 hours
mTBD	25.5	80 °C	95.2 hours
P_1-^{t}Bu	27.0	80 °C	>7 days

A range of decomposition products are formed from the various rotaxane superbases which are not trivial to identify unambiguously. As shown in Figure S2c, hydrolytic decomposition of 24C8⊂2•HPF6 results in two new sets of aromatic signals (7.3 ppm, 2H; 7.6 ppm, 1H; 7.9 ppm, 1H; 8.5 ppm, 2H), two new benzylic methylene signals (4.3 ppm, 2H; 3.9 ppm, 2H) and a new tert-butyl signal (1.4 ppm, 9H). These integrals are relative to each other but under-integrate relative to a new broad singlet that appears at 5.5 ppm, as well as two triplets (3.6 ppm, 2H, 3.4 ppm, 2H) and a 3H singlet (3.7 ppm).

A tentative explanation of these results is that ammonium group binding may activate the crown ether to an S_N2 reaction with sodium methoxide, giving *O*-methyl octaethyleneglycol, which would be consistent with the two new 2H triplets (the methylenes adjacent to the new hydroxyl and methyl ether groups) and the 3H singlet (the methyl ether). The dibenzylamine thread may hydrolyze further to give the corresponding primary benzylamine and benzyl alcohol, consistent with the new sets of aromatic signals, the benzylic methylene signals and the tert-butyl signal. A colorless precipitate forms in the decomposition reaction, which likely corresponds to the aromatic decomposition products, as their signals under-integrate relative to the putative *O*-methyl octaethyleneglycol. In these processes, two new hydroxyl groups are generated: one in the *O*-methyl octaethyleneglycol and one in the benzyl alcohol. The protons on these groups are in fast exchange with each other and with trace CD_3OH, consistent with the broad singlet at 5.5 ppm.

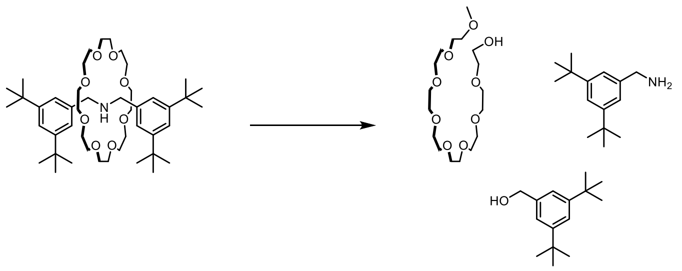

5.0 X-Ray Crystallography

General Procedure – Crystals suitable for X-ray diffraction were grown by dissolving a rotaxane HPF_6 salt (5–20 mgs) in a minimum of anhydrous THF in a thin (1 cm diameter) 2 mL vial. A vial cap was pierced several times and used to cap the vial. To a separate 20 mL vial (large enough to contain the thin vial) was added 5 mL of anhydrous hexane as antisolvent. The thin vial containing the rotaxane HPF_6 salt solution was placed in the larger antisolvent vial and the larger antisolvent vial was capped and sealed with parafilm. The antisolvent crystallization then proceeded over 1-2 weeks affording the desired crystals.

Data Collection. X-Ray data for compounds **24C8⊂3**•HBr, **DB24C8⊂3**•HPF_6 and **21C7⊂3**•HBr were collected at 100 K on a Rigaku FR-X rotating anode equipped with Hypix-6000HE detectors and oxford cryosystem. Data were measured using CrysAlisPro suite of programs.

Crystal structure determinations and refinements. X-ray data were processed and reduced using CrysAlisPro suite of programmes. Absorption correction was performed using empirical methods (SCALE3 ABSPACK) based upon symmetry-equivalent reflections combined with measurements at different azimuthal angles. The crystal structures were solved and refined against all F^2 values using the SHELX and Olex 2 suite of programmes. X-ray data for **24C8⊂3**•HBr diffracted to 1 Å and were found to be modulated with a q vector of (0,0,1/2). The small unit cell setting was chosen due to the large amount of parameters of the large unit cell setting. Crystal structures **24C8⊂3**•HBr, **DB24C8⊂3**•HPF_6 **and 21C7⊂3**•HBr presented a large amount of disorder in the CF_3, macrocycles and anions. The disorder was modelled over two positions, where atomic distances were restrained using distance restrains (SHELX; DFIX and SADI). The atomic displacement parameters (adp) of the CF_3 and the macrocycles were restrained using rigid body restrains (SHELX RIGU and SIMU commands). In order to maximize the data/parameter ratio, large amount of disordered moieties were refined isotropically in **24C8⊂3**•HBr. Hydrogen atoms were placed in the calculated positions.

Table S3 – Crystallographic parameters for **24C8⊂3**•HBr, **DB24C8⊂3**•HPF_6 and **21C7⊂3**•HBr.

Identification code	**24C8⊂3**•HBr	**DB24C8⊂3**•HPF_6	**21C7⊂3**•HBr
Empirical formula	$C_{32}H_{42}BrF_{12}NO_8$	$C_{42}H_{44}F_{18}NO_8P$	$C_{34}H_{44}BrF_{12}NO_8$
Formula weight	876.57	1063.75	902.61
Temperature/K	200.01(10)	200.01(10)	99.99(10)
Crystal system	triclinic	monoclinic	orthorhombic
Space group	P-1	$P2_1/n$	$P2_12_12_1$
a/Å	22.2505(5)	13.3929(5)	11.63400(10)
b/Å	28.5022(6)	13.2689(4)	15.55150(10)
c/Å	29.4662(6)	29.4371(12)	21.6851(2)
α/°	81.586(2)	90	90
β/°	67.825(2)	91.679(4)	90
γ/°	67.063(2)	90	90
Volume/Å^3	15936.7(7)	5229.0(3)	3923.40(6)
Z	16	4	4
ρ_{calc}g/cm^3	1.461	1.351	1.528
μ/mm^{-1}	2.338	1.473	2.392
F(000)	7168.0	2176.0	1848.0
Crystal size/mm^3	0.234 × 0.158 × 0.132	0.28 × 0.16 × 0.061	0.5 × 0.12 × 0.11
Radiation	Cu Kα (λ = 1.54184)	Cu Kα (λ = 1.54184)	Cu Kα (λ = 1.54184)
2Θ range for data collection/°	4.606 to 100.872	6.008 to 152.682	6.994 to 151.568
Index ranges	-21 ≤ h ≤ 21, -28 ≤ k ≤ 28, -29 ≤ l ≤ 29	-16 ≤ h ≤ 16, -15 ≤ k ≤ 16, -36 ≤ l ≤ 30	-14 ≤ h ≤ 13, -19 ≤ k ≤ 17, -23 ≤ l ≤ 27
Reflections collected	31930	31448	26922
Independent reflections	31930 [R_{int} = ?, R_{sigma} = 0.0423]	10611 [R_{int} = 0.0218, R_{sigma} = 0.0291]	7872 [R_{int} = 0.0244, R_{sigma} = 0.0214]
Data/restraints/parameters	31930/5471/3774	10611/1335/798	7872/1078/704
Goodness-of-fit on F^2	1.702	1.084	1.028
Final R indexes [I>=2σ (I)]	R_1 = 0.1362, wR_2 = 0.3584	R_1 = 0.0639, wR_2 = 0.1963	R_1 = 0.0325, wR_2 = 0.0892

Final R indexes [all data]	R_1 = 0.1515, wR_2 = 0.3757	R_1 = 0.0943, wR_2 = 0.2253	R_1 = 0.0335, wR_2 = 0.0899
Largest diff. peak/hole / e Å^{-3}	1.10/-1.13	0.35/-0.24	0.42/-0.64
Flack parameter			-0.027(4)

CCDC identifiers 2238956, 2238957 and 2238958 contain the supplementary crystallographic data for this paper. These data can be obtained free of charge via www.ccdc.cam.ac.uk/conts/retrieving.html (or from the Cambridge Crystallographic Data Centre, 12 Union Road, Cambridge CB21EZ, UK; fax: (+44)1223-336-033; or deposit@ccdc.cam.ac.uk).

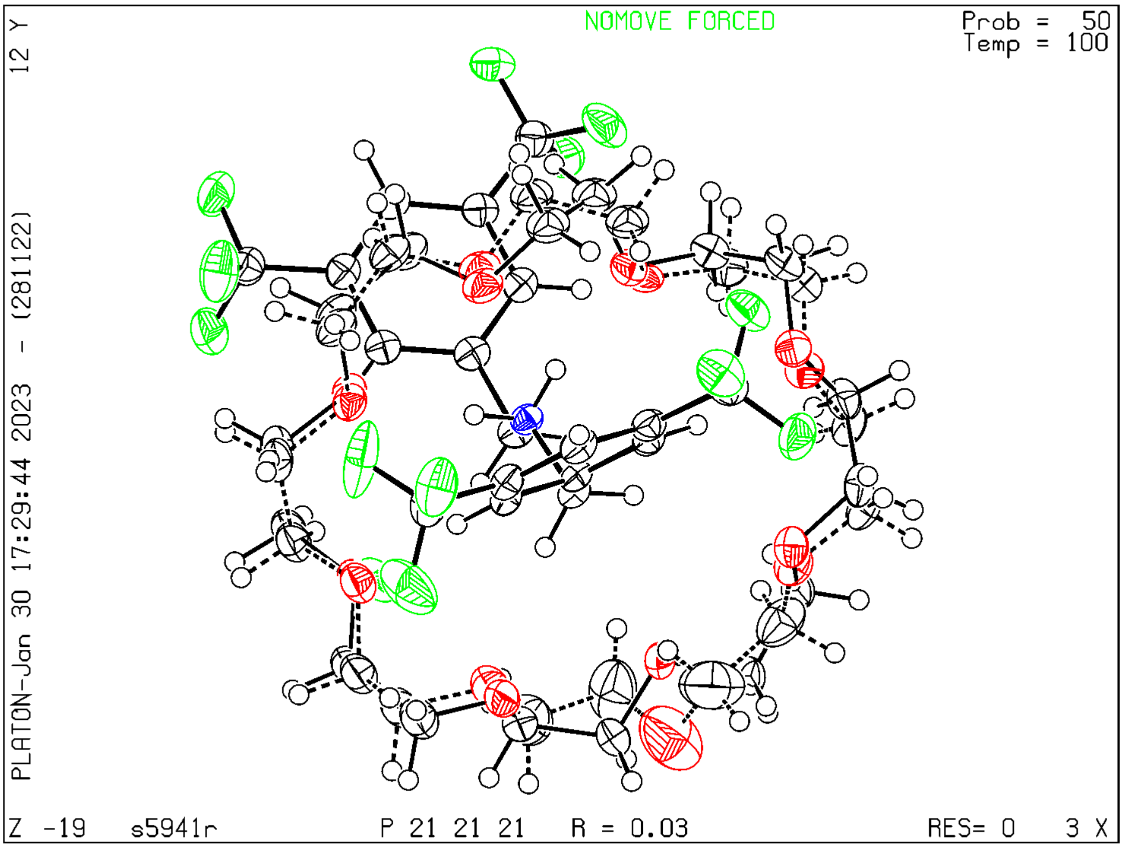

Figure S3 – X-ray crystal structure of **24C8⊂3**•HBr.

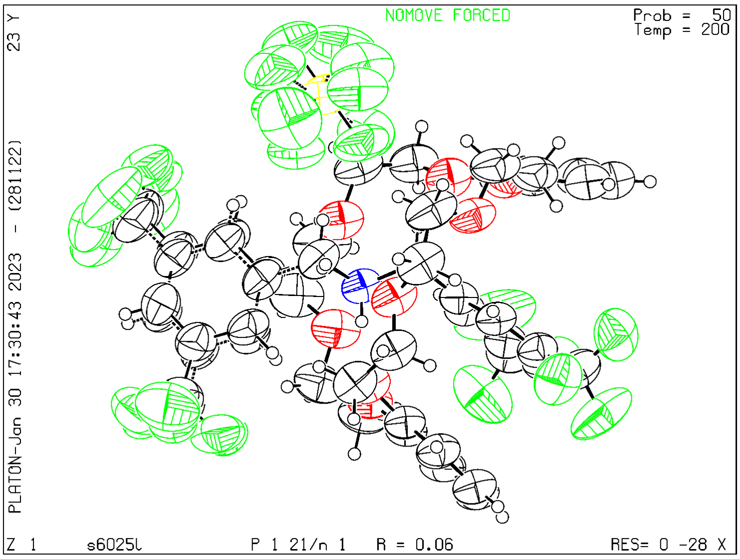

Figure S4 – X-ray crystal structure of **DB24C8⊂3**•HPF_6.

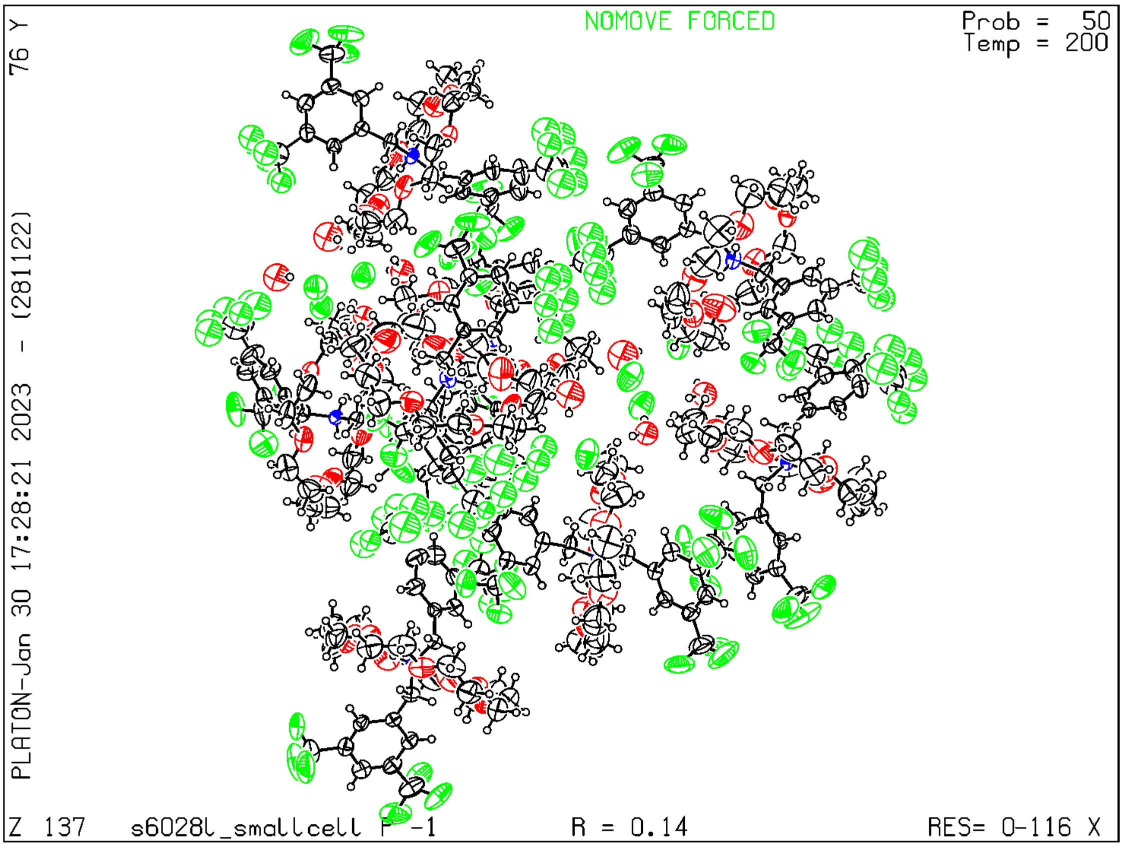

Figure S5 – X-ray crystal structure of **21C7⊂3**•HBr.

6.0 NMR Spectra of Novel Compounds

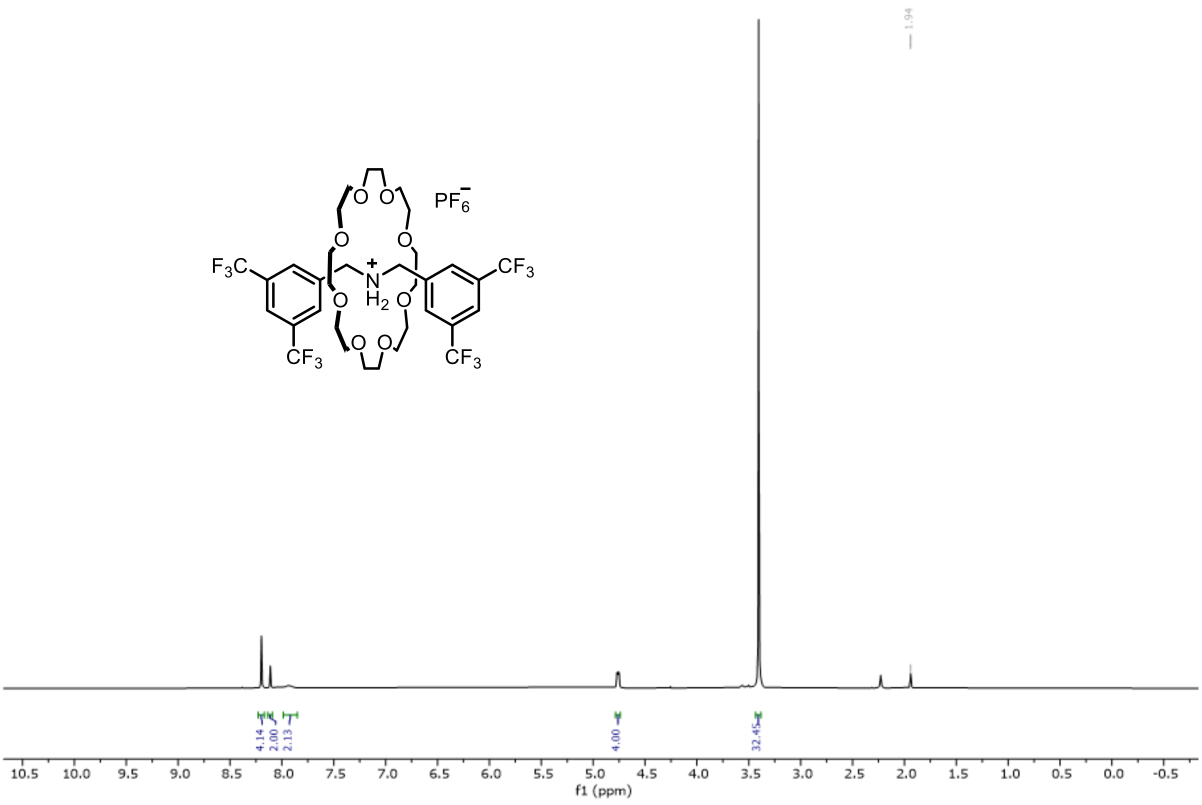

Figure S6 – ^{1}H NMR spectrum of 24C8⊂3•HPF$_6$ (600 MHz, CD$_3$CN).

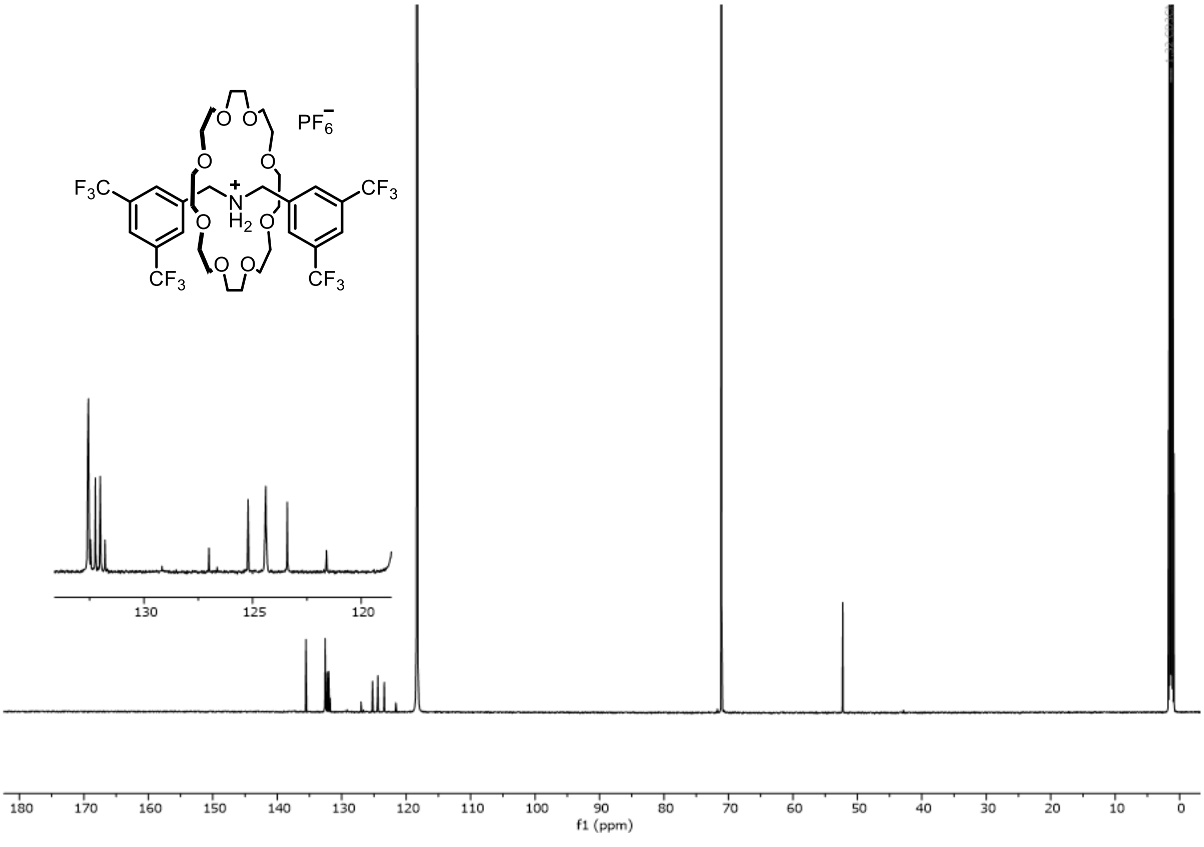

Figure S7 – ^{13}C NMR spectrum of 24C8⊂3•HPF$_6$ (151 MHz, CD$_3$CN).

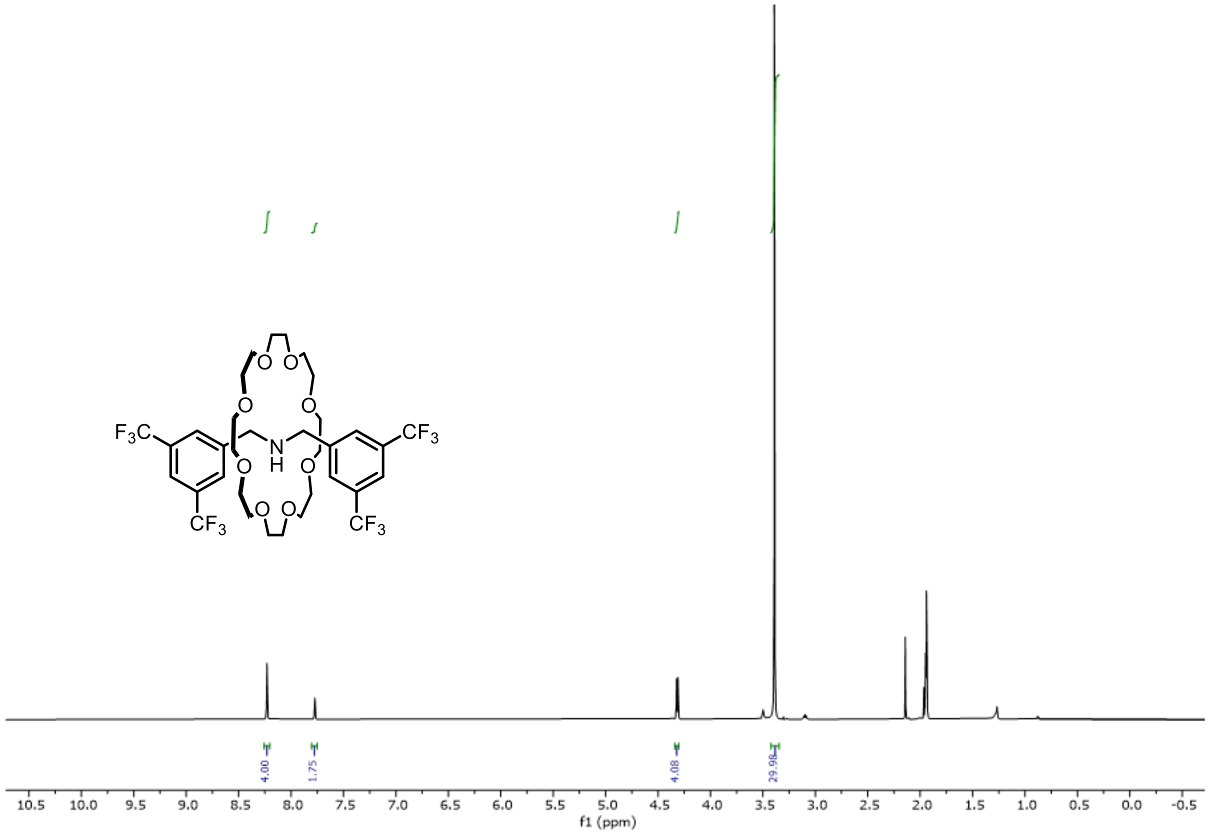

Figure S8 – ^{1}H NMR spectrum of 24C8⊂3 (600 MHz, CD_3CN).

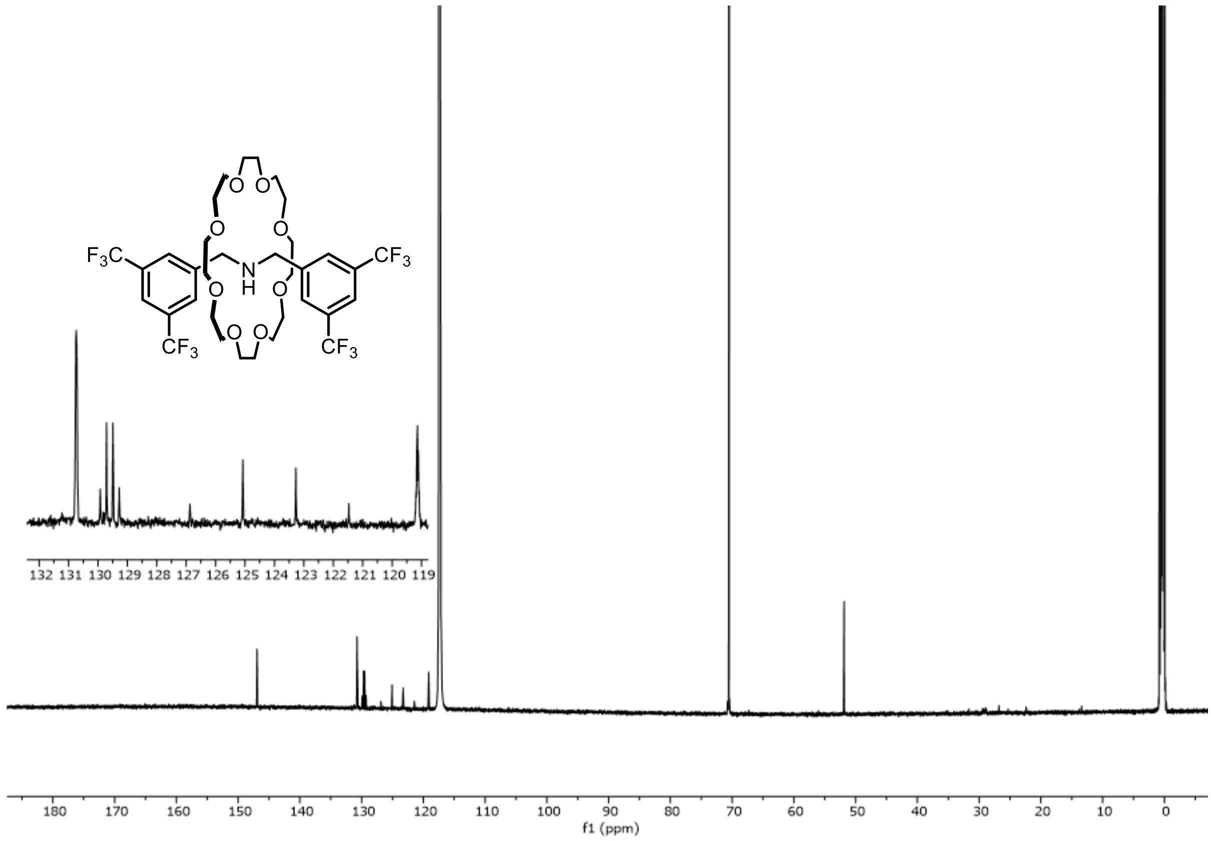

Figure S9 – ^{13}C NMR spectrum of 24C8⊂3 (151 MHz, CD_3CN).

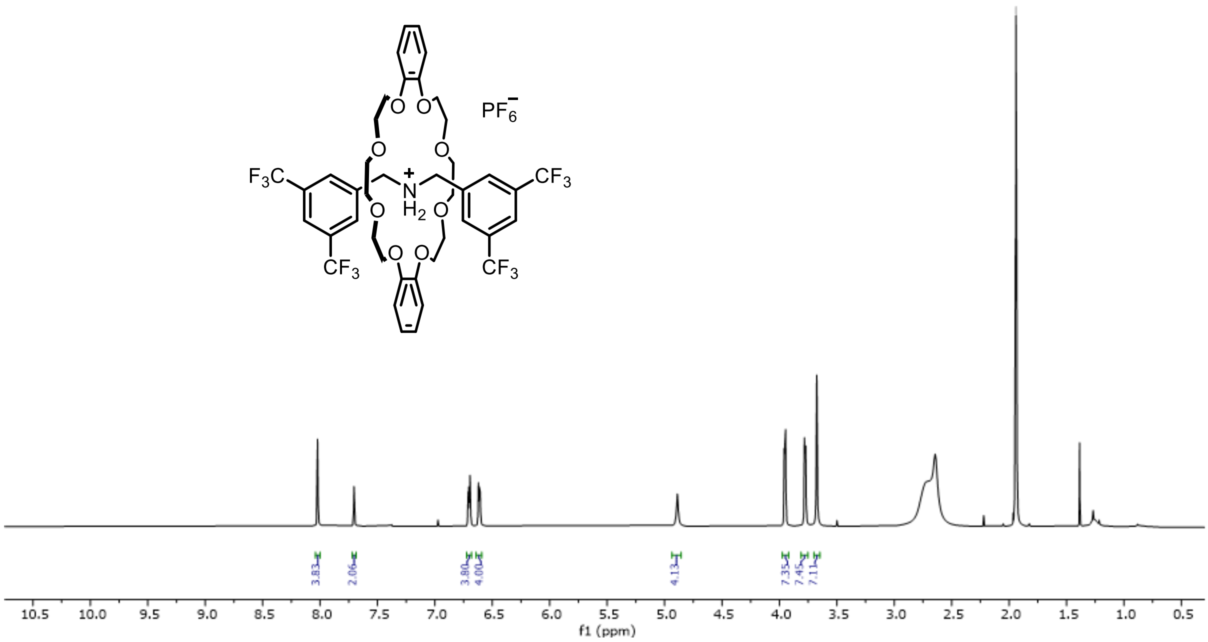

Figure S10 – ^{1}H NMR spectrum of DB24C8⊂3•HPF$_6$ (600 MHz, CD$_3$CN).

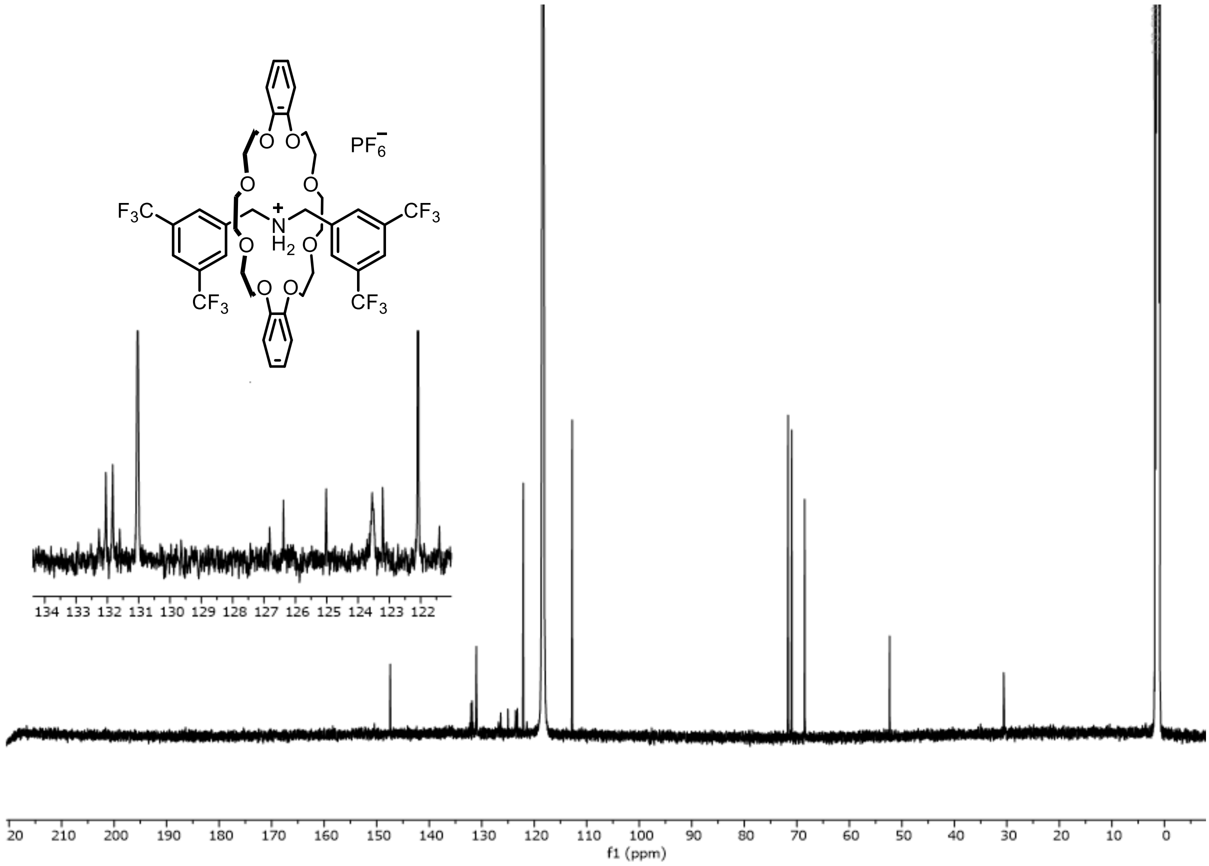

Figure S11 – ^{13}C NMR spectrum of DB24C8⊂3•HPF$_6$ (151 MHz, CD$_3$CN).

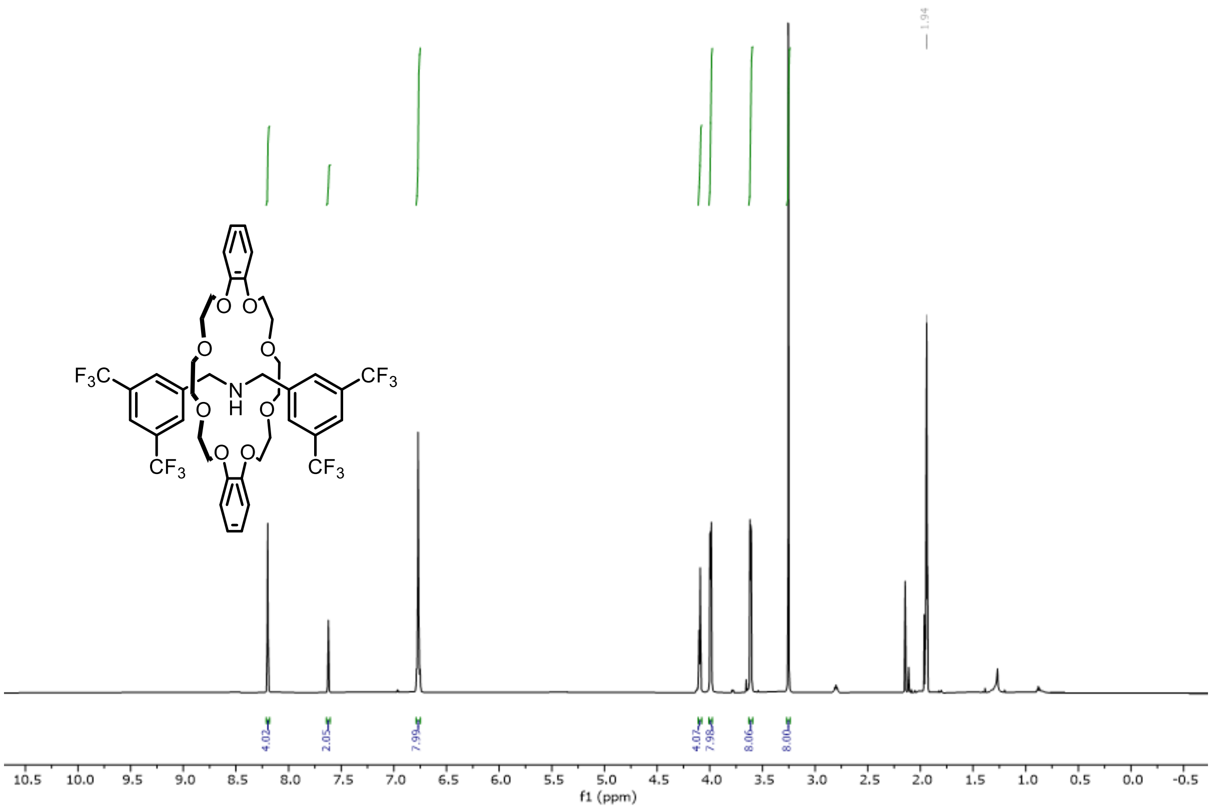

Figure S12 – ^{1}H NMR spectrum of DB24C8⊂3 (600 MHz, CD_3CN).

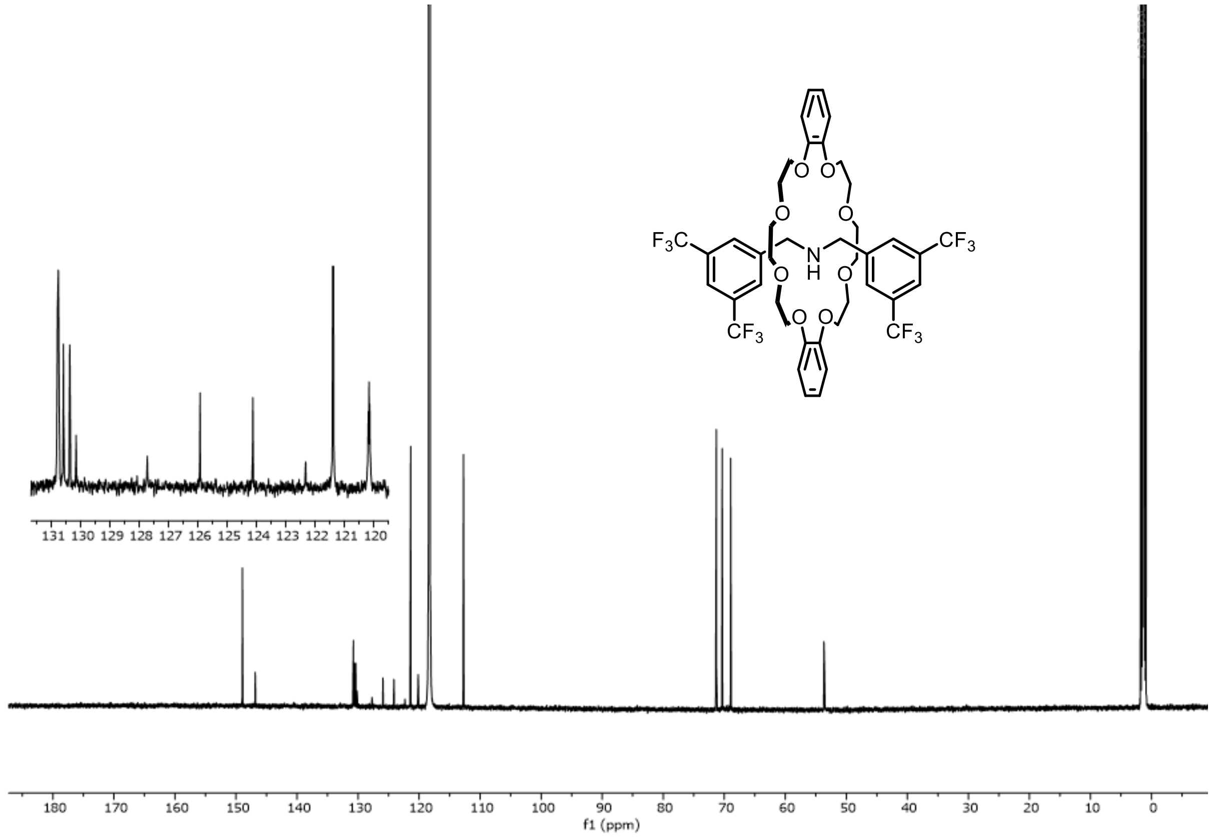

Figure S13 – ^{13}C NMR spectrum of DB24C8⊂3 (151 MHz, CD_3CN).

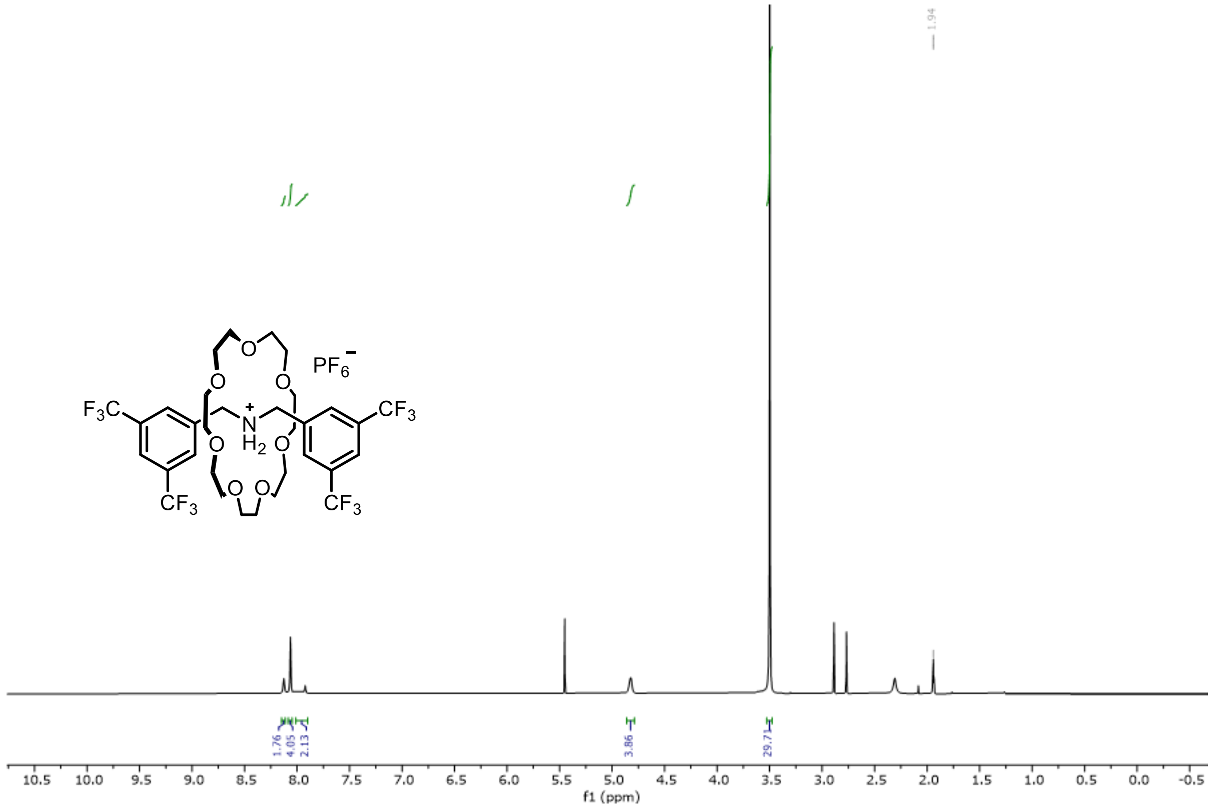

Figure S14 – ^{1}H NMR spectrum of 21C7⊂3•HPF$_6$ (600 MHz, CD$_3$CN).

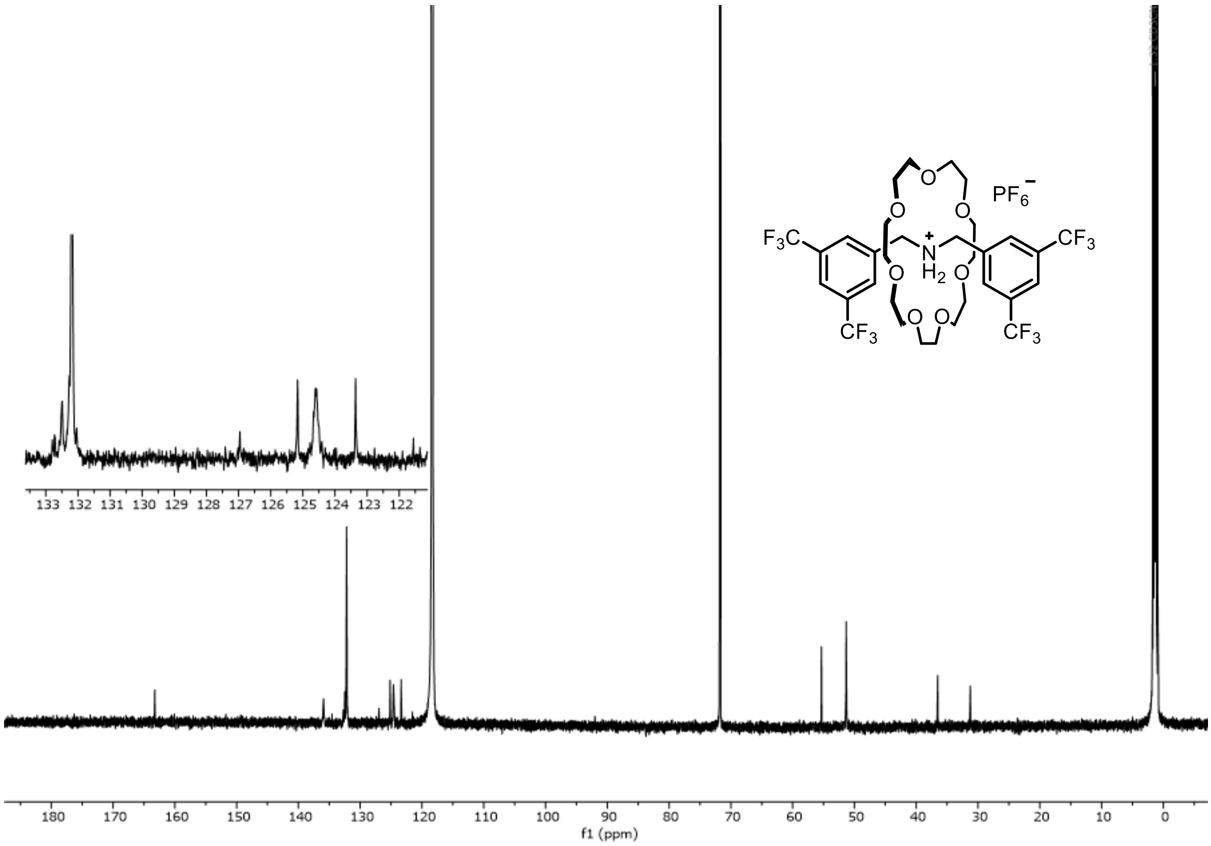

Figure S15 – ^{13}C NMR spectrum of 21C7⊂3•HPF$_6$ (151 MHz, CD$_3$CN).

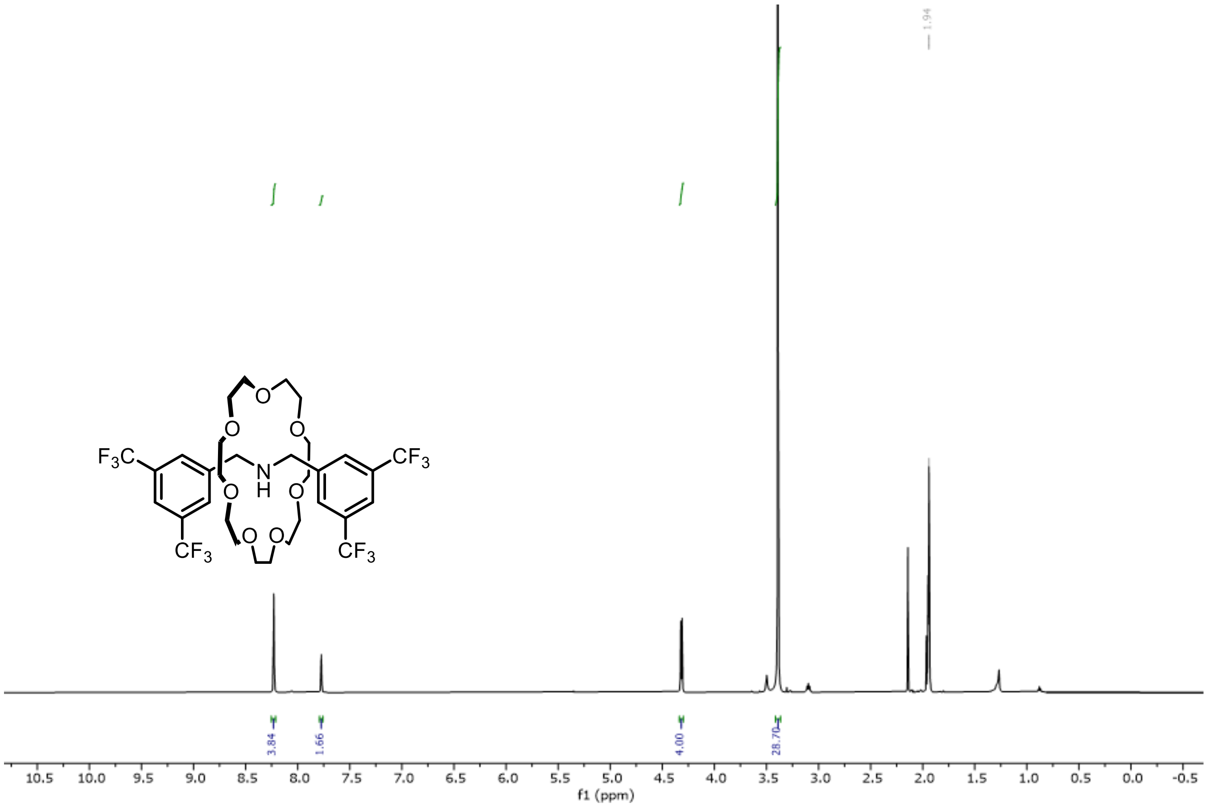

Figure S16 – ^{1}H NMR spectrum of 21C7⊂3 (600 MHz, CD_3CN).

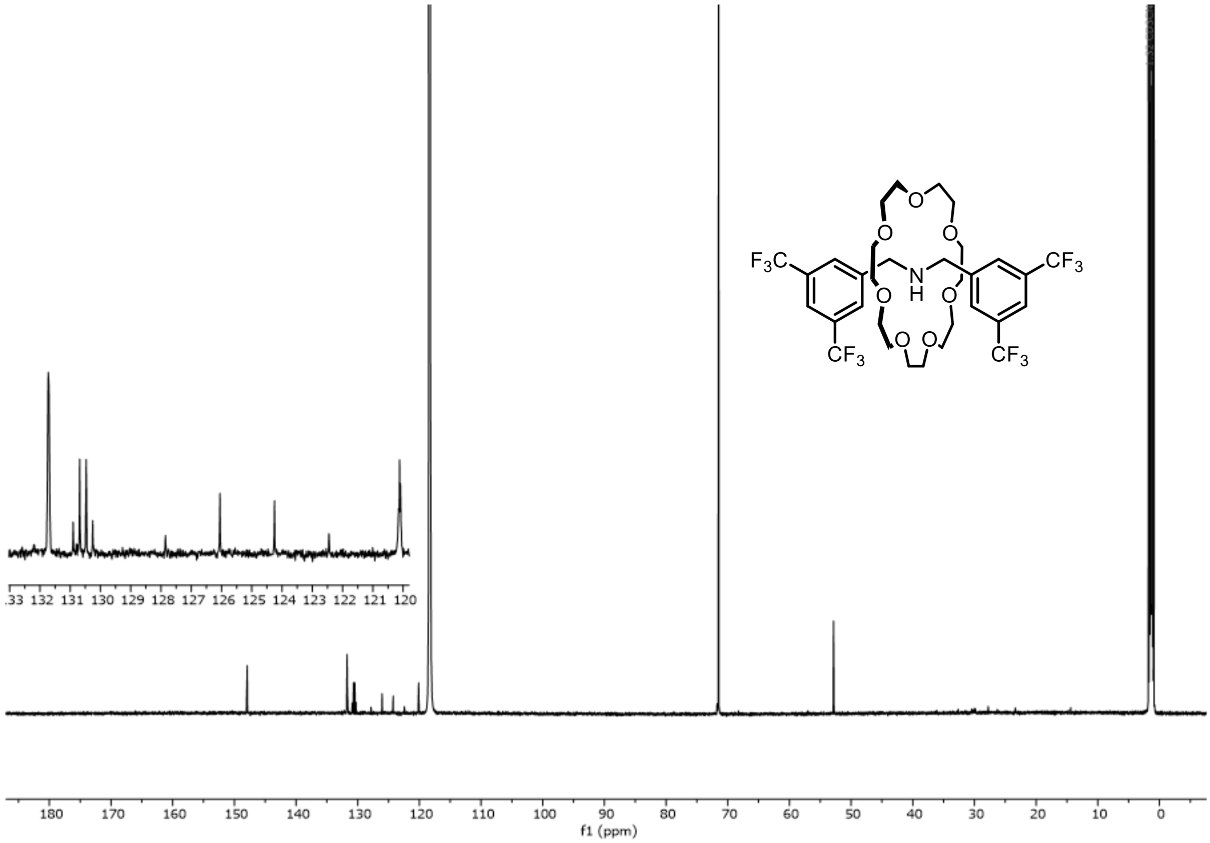

Figure S17 – ^{13}C NMR spectrum of 21C7⊂3 (151 MHz, CD_3CN).

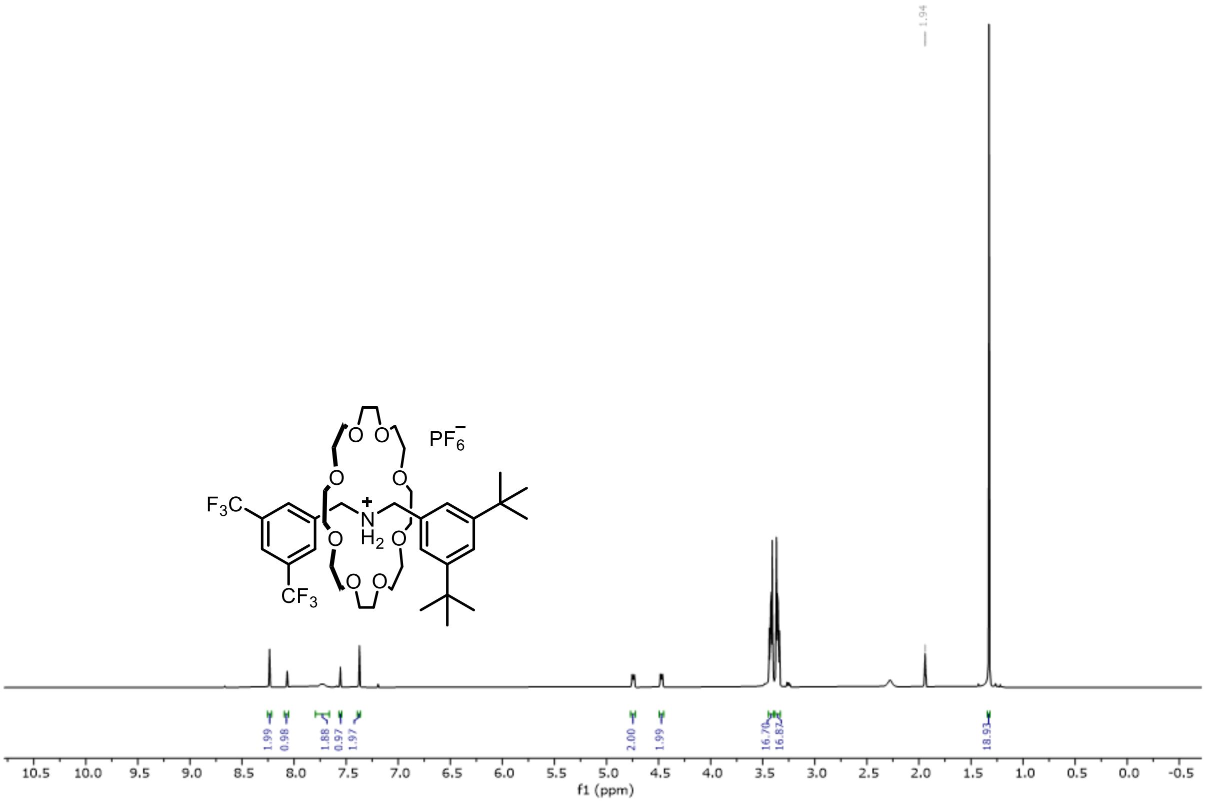

Figure S18 – ^{1}H NMR spectrum of 24C8⊂4•HPF$_6$ (600 MHz, CD$_3$CN).

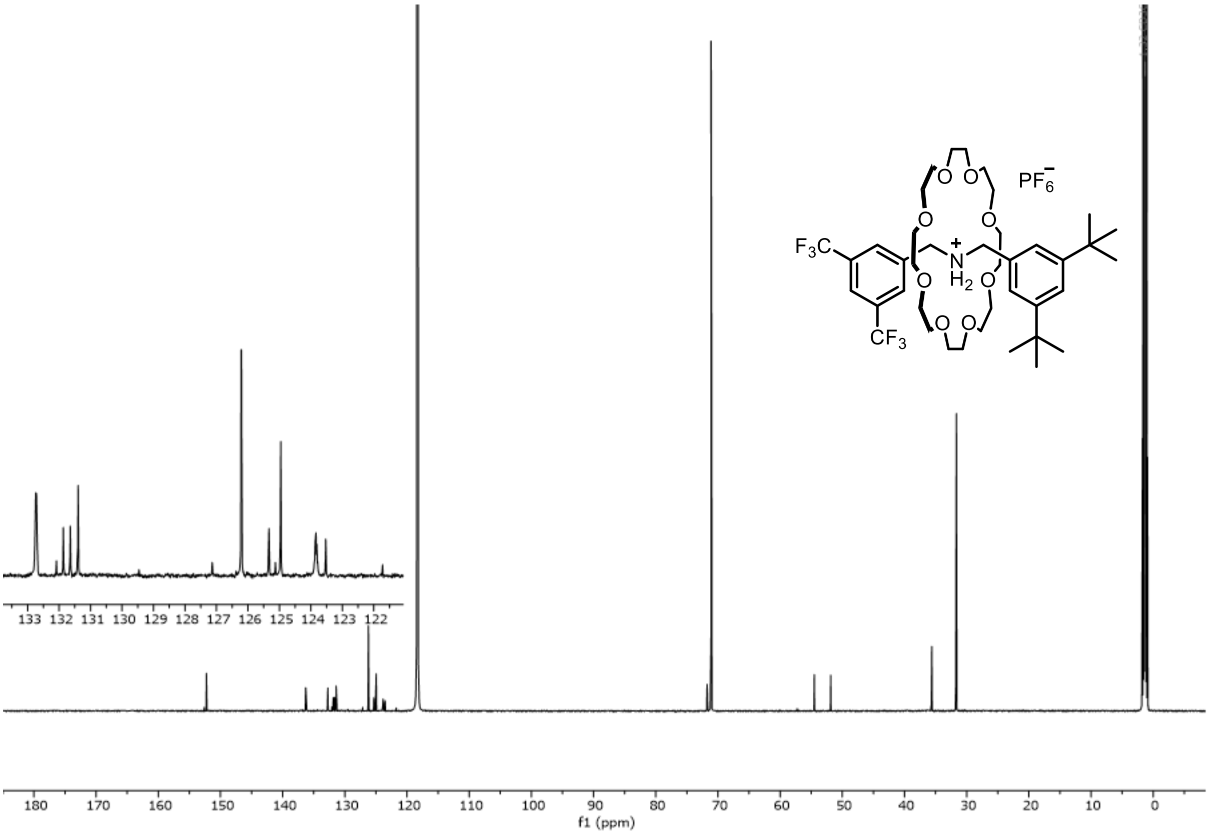

Figure S19 – ^{13}C NMR spectrum of 24C8⊂4•HPF$_6$ (151 MHz, CD$_3$CN).

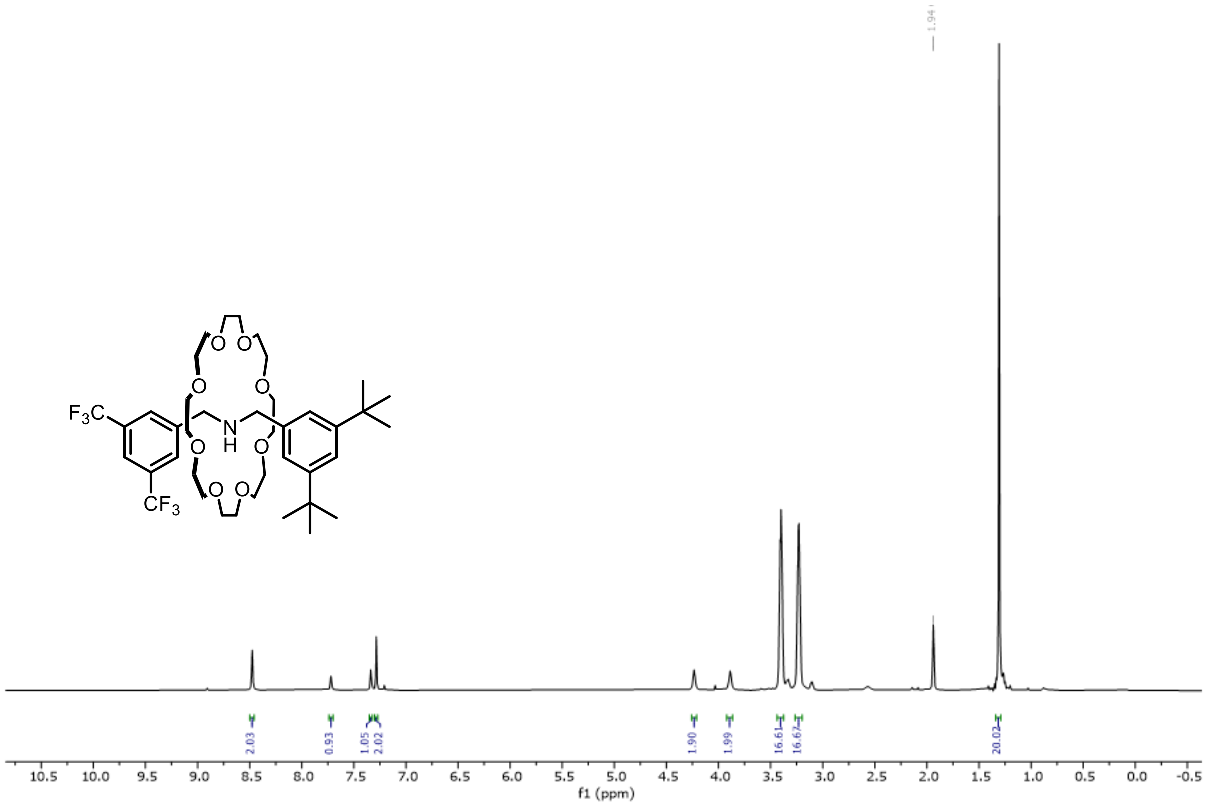

Figure S20 – ^{1}H NMR spectrum of 24C8⊂4 (600 MHz, CD_3CN).

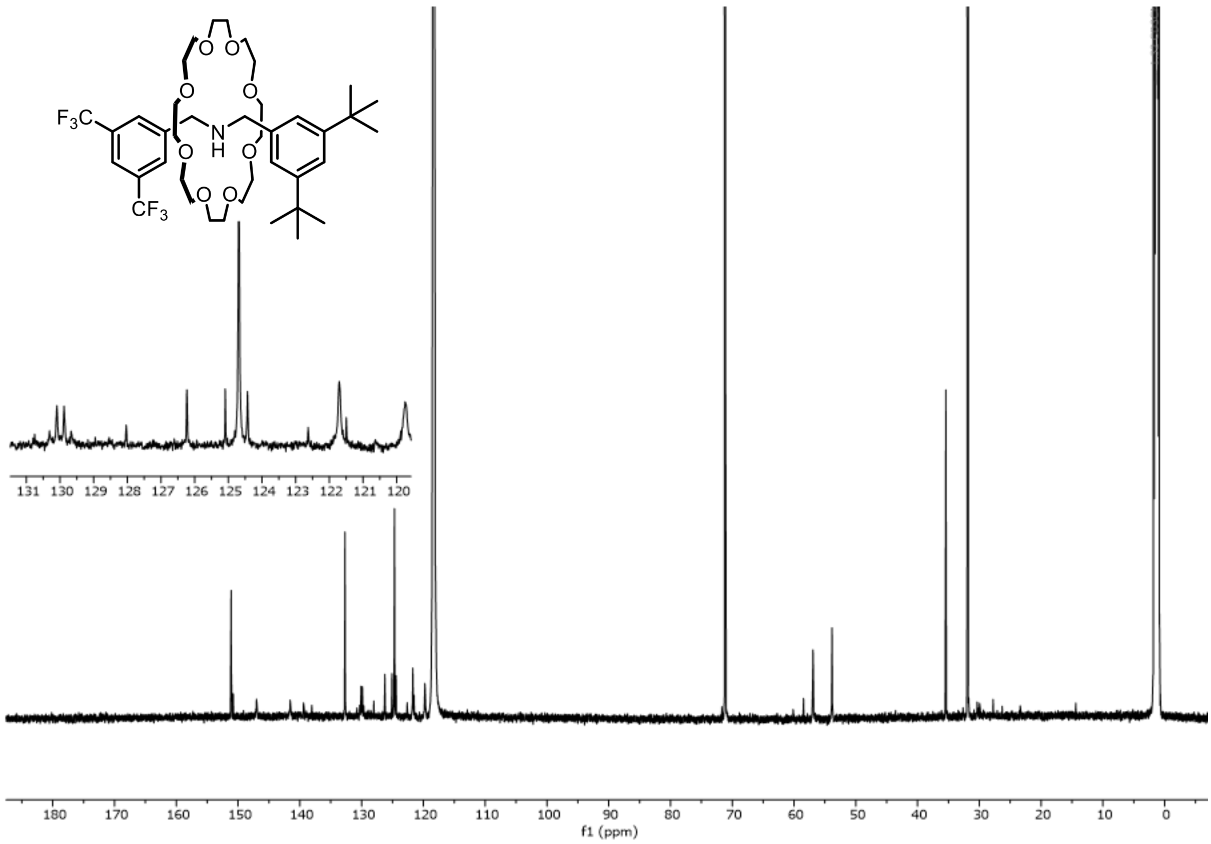

Figure S21 – ^{13}C NMR spectrum of 24C8⊂4 (151 MHz, CD_3CN).

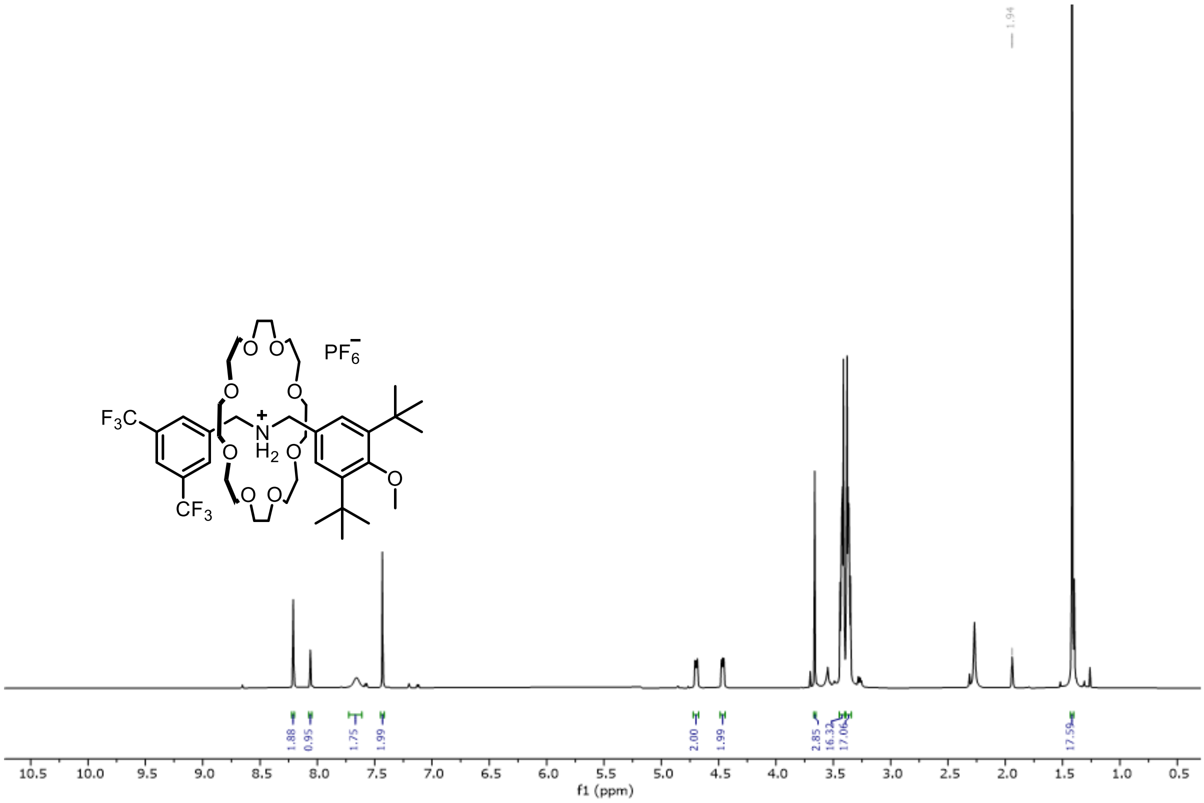

Figure S22 – ^{1}H NMR spectrum of 24C8⊂5•HPF$_6$ (600 MHz, CD$_3$CN).

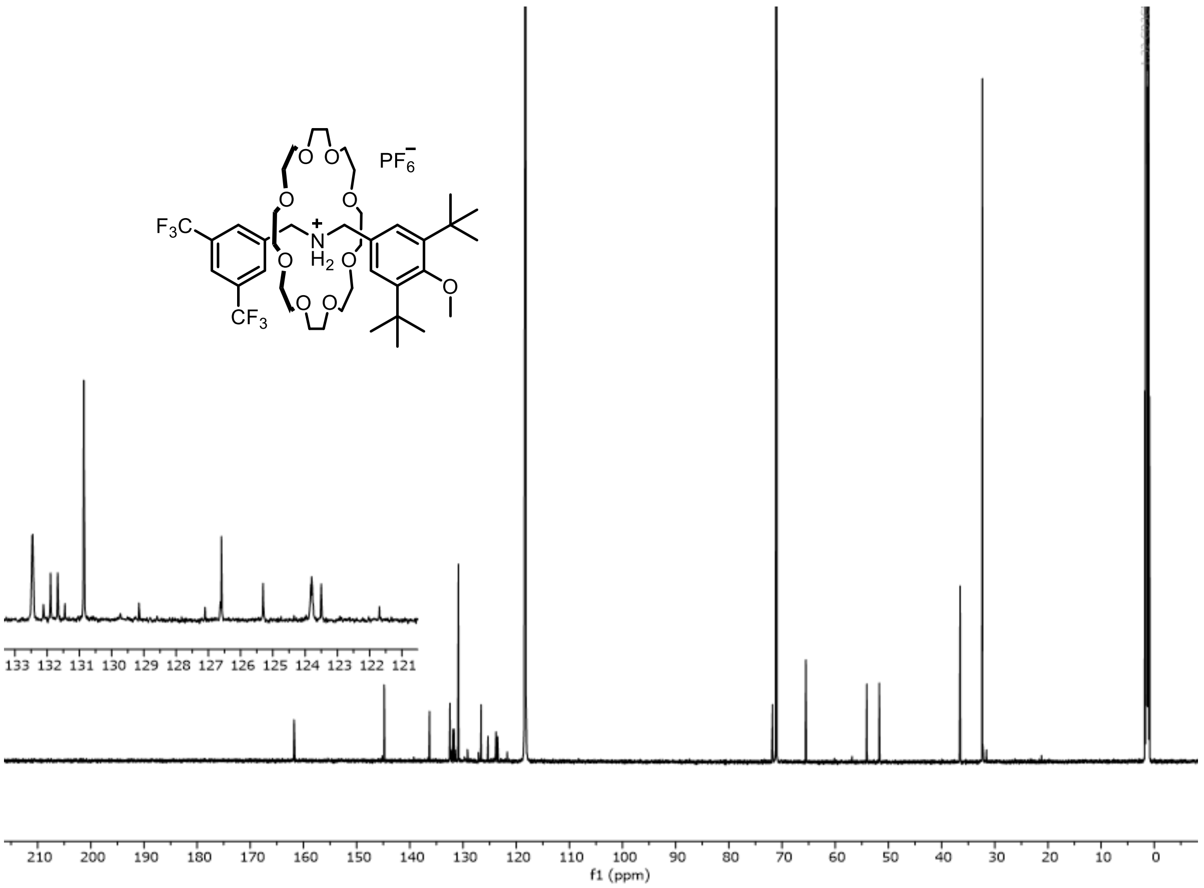

Figure S23 – ^{13}C NMR spectrum of 24C8⊂5•HPF$_6$ (151 MHz, CD$_3$CN).

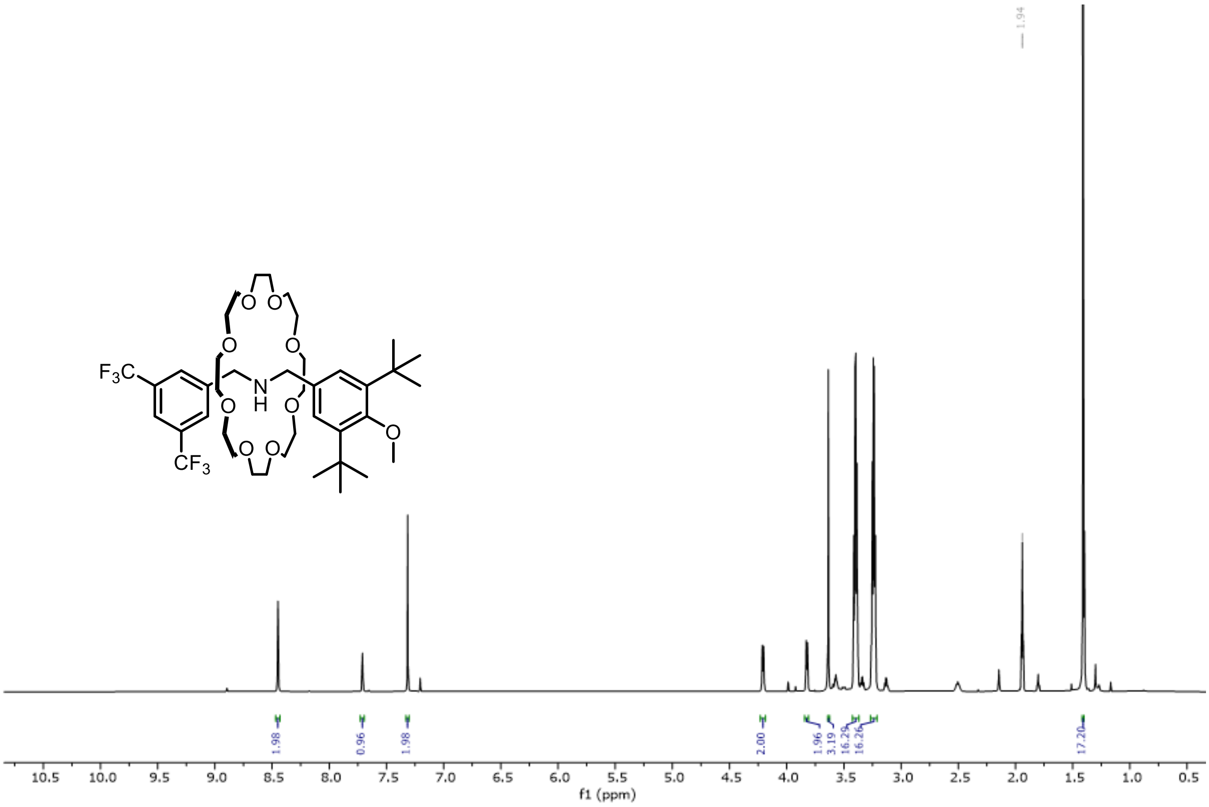

Figure S24 – ^{1}H NMR spectrum of 24C8⊂5 (600 MHz, CD_3CN).

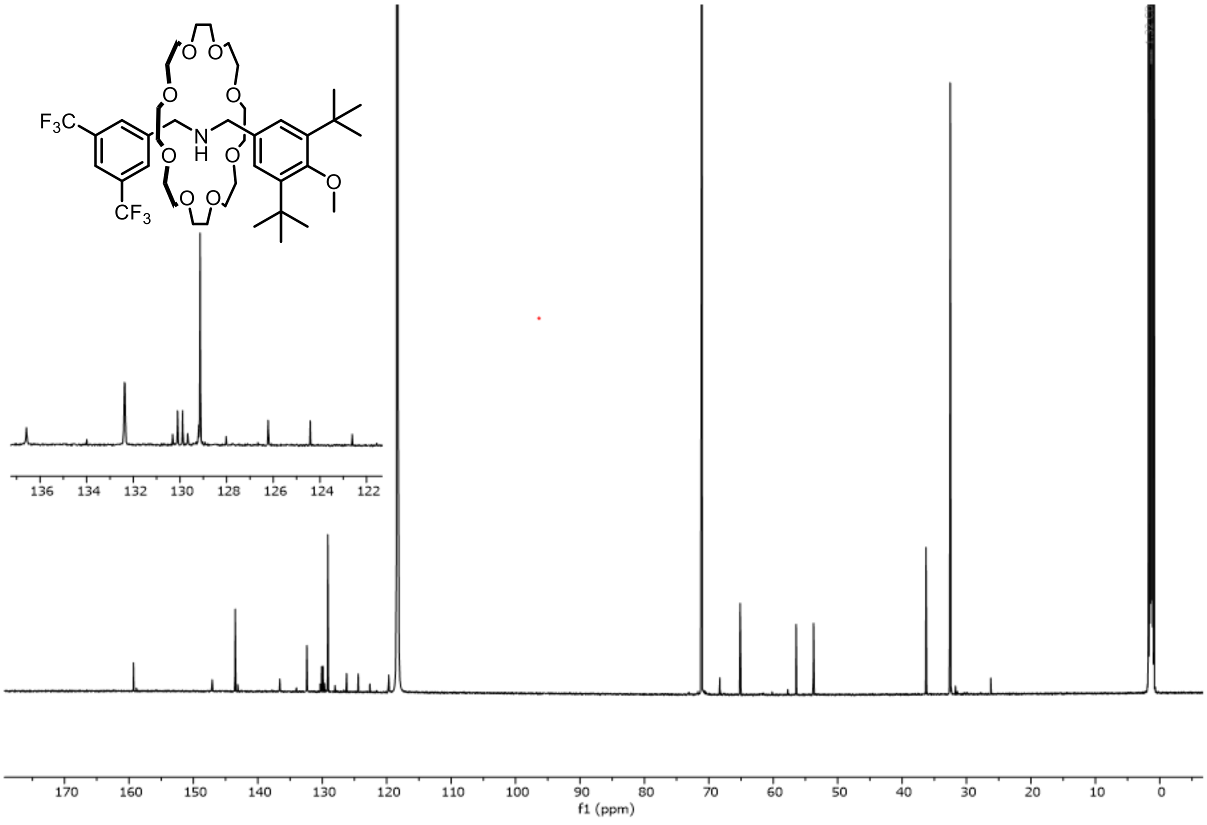

Figure S25 – ^{13}C NMR spectrum of 24C8⊂5 (151 MHz, CD_3CN).

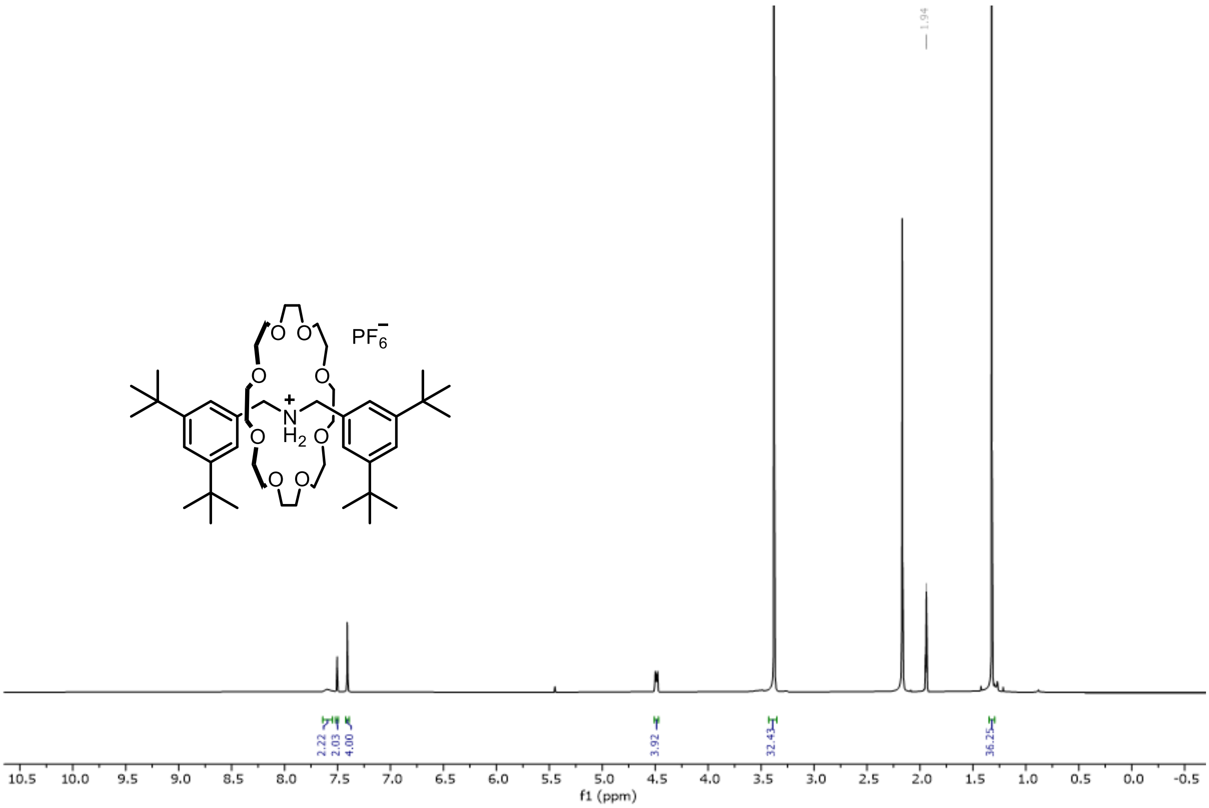

Figure S26 – ^{1}H NMR spectrum of 24C8⊂2•HPF$_6$ (600 MHz, CD$_3$CN).

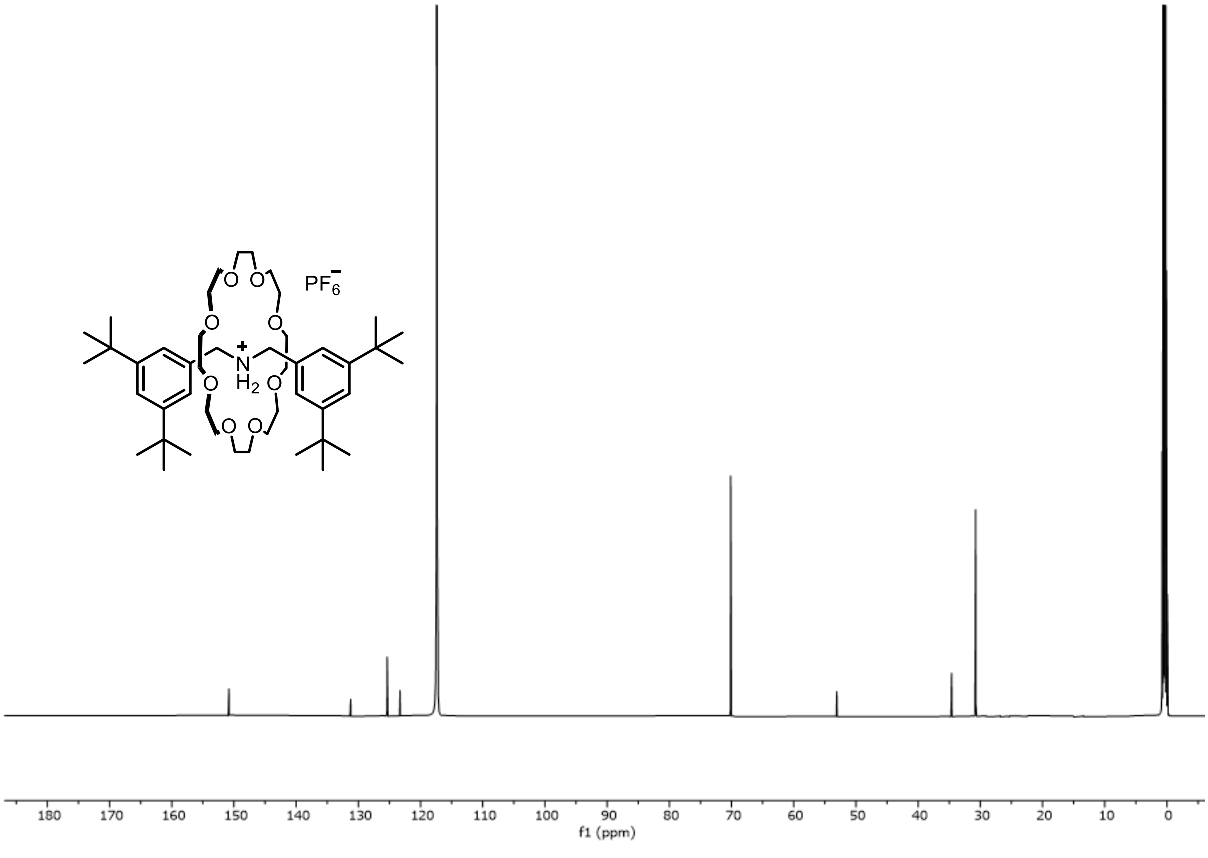

Figure S27 – ^{13}C NMR spectrum of 24C8⊂2•HPF$_6$ (151 MHz, CD$_3$CN).

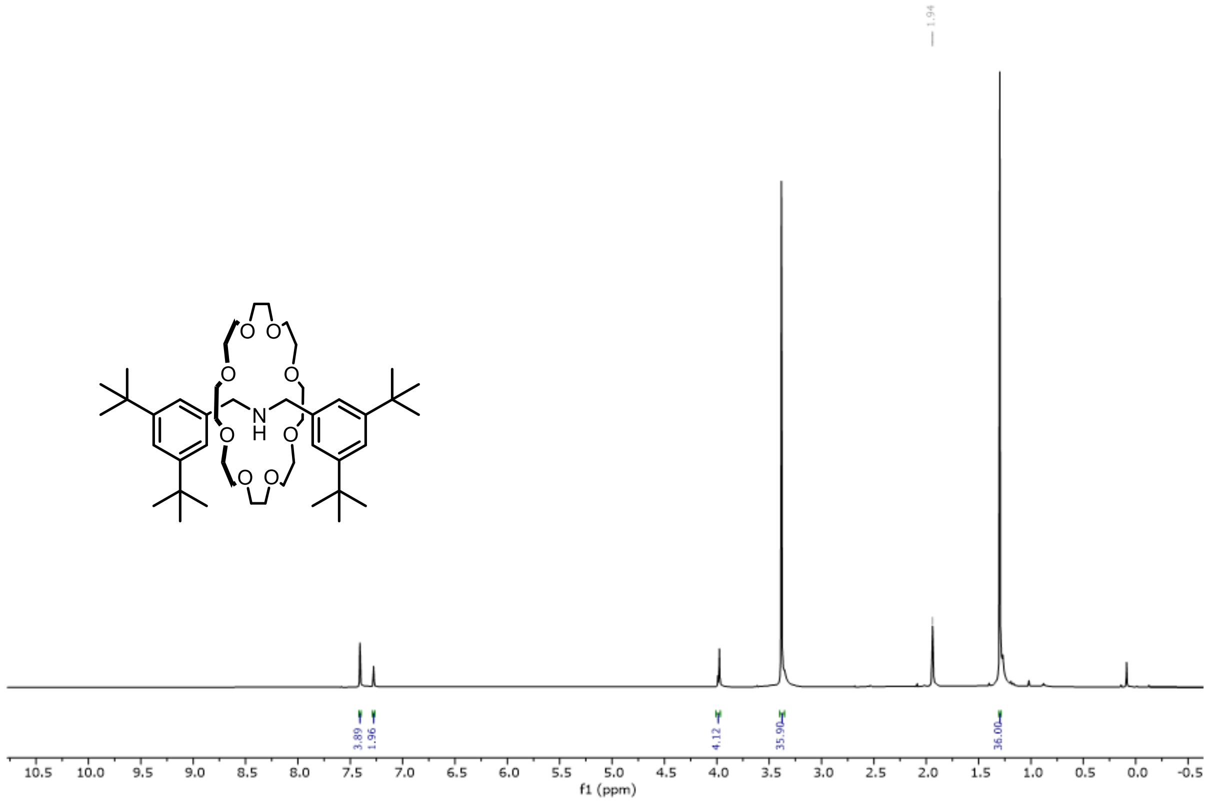

Figure S28 – ^{1}H NMR spectrum of 24C8⊂2 (600 MHz, CD_3CN).

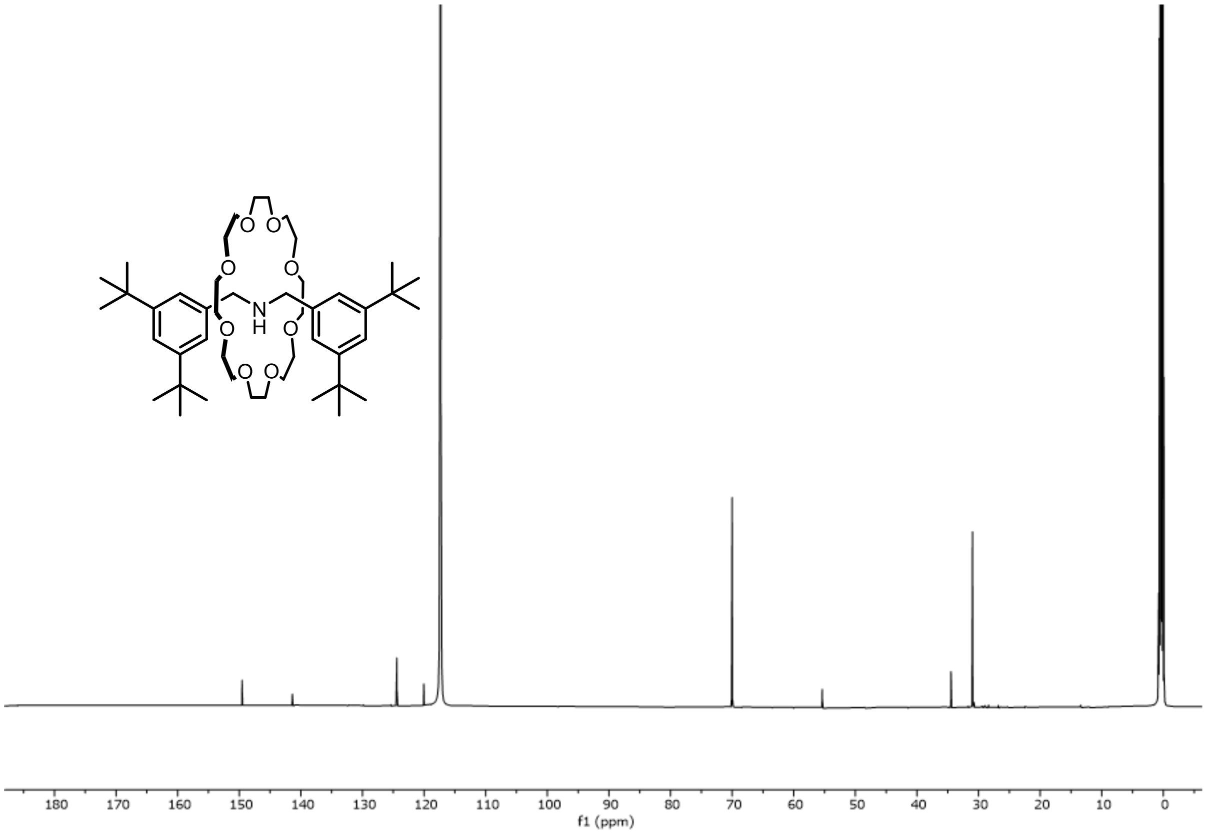

Figure S29 – ^{13}C NMR spectrum of 24C8⊂2 (151 MHz, CD_3CN).

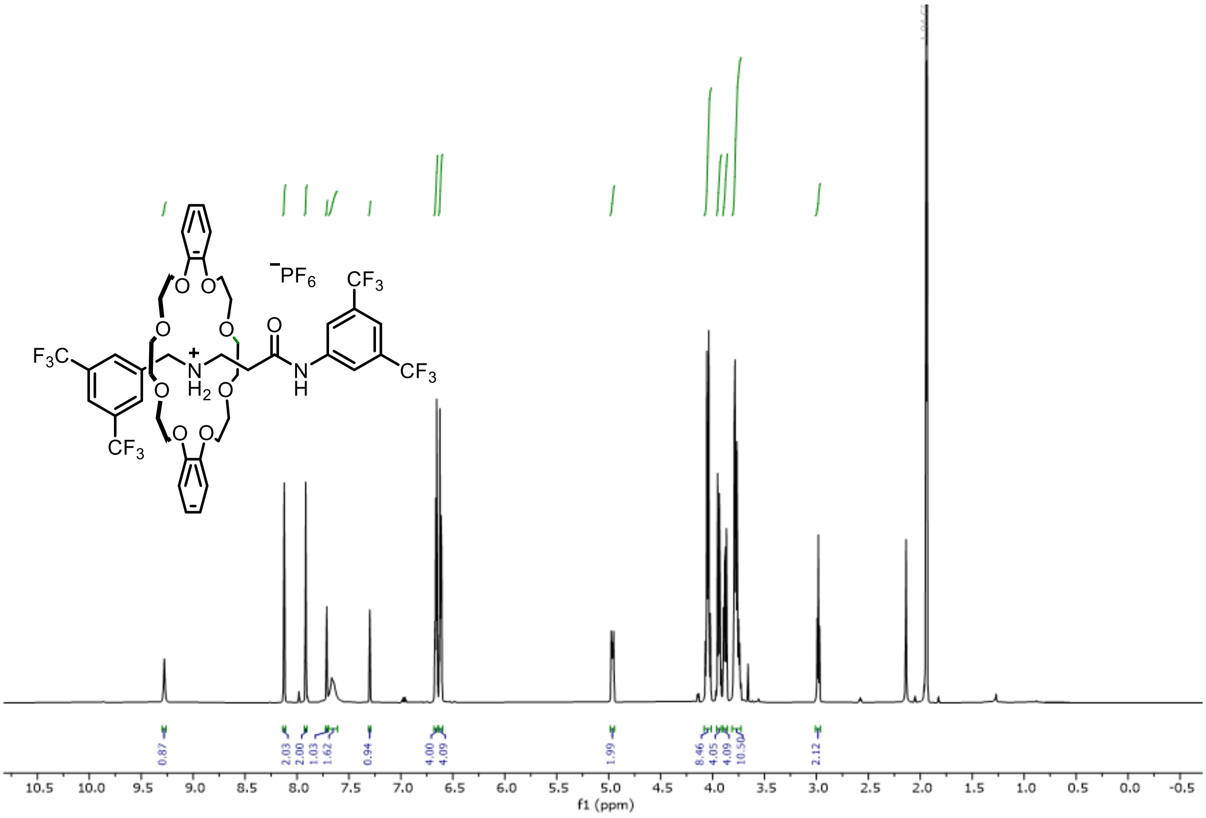

Figure S30 – ^{1}H NMR spectrum of 24C8⊂8•HPF$_6$ (600 MHz, CD$_3$CN).

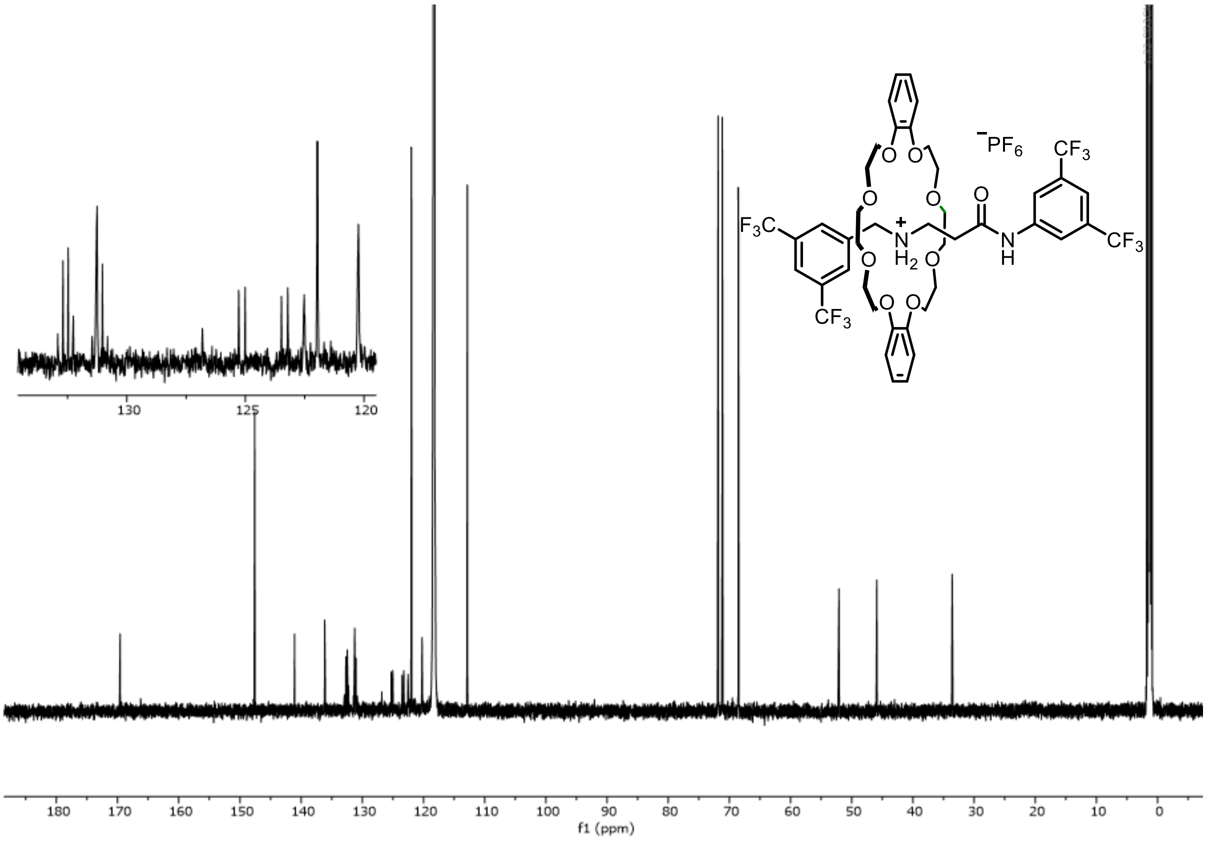

Figure S31 – ^{13}C NMR spectrum of 24C8⊂8•HPF$_6$ (151 MHz, CD$_3$CN).

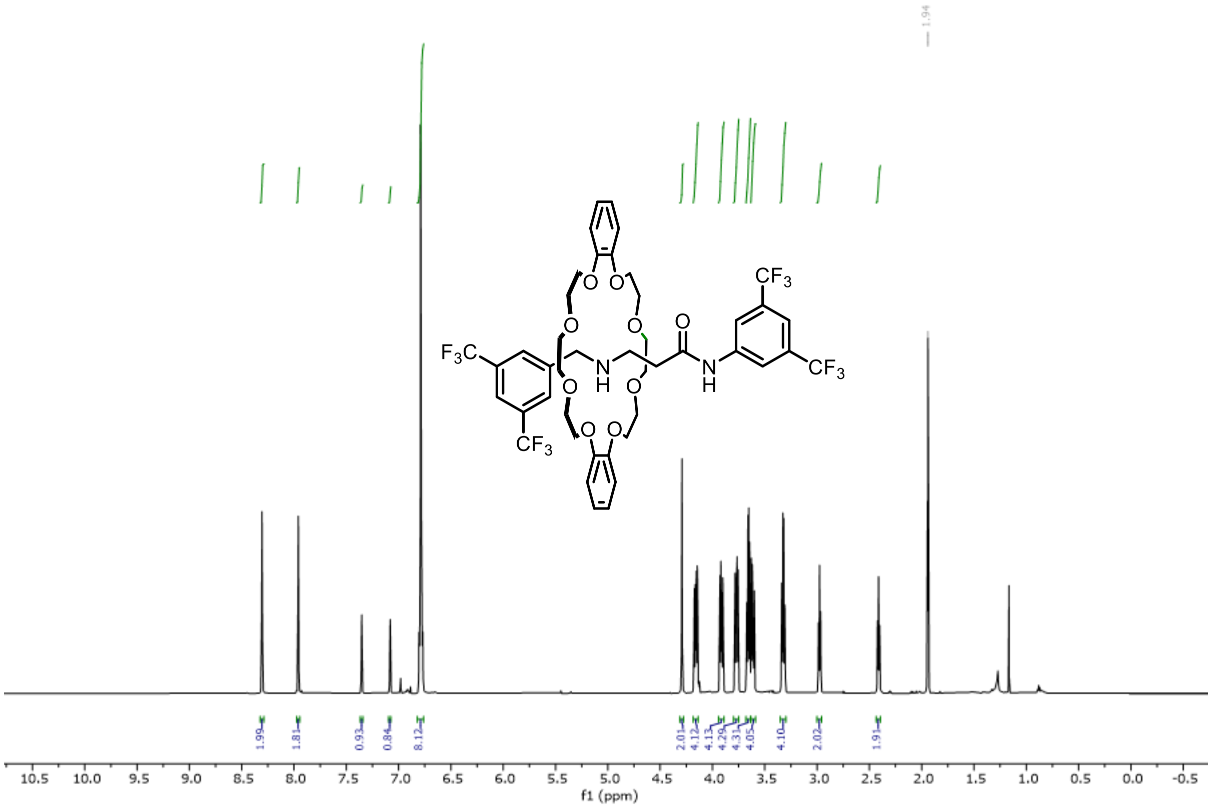

Figure S32 – ^{1}H NMR spectrum of 24C8⊂8 (600 MHz, CD_3CN).

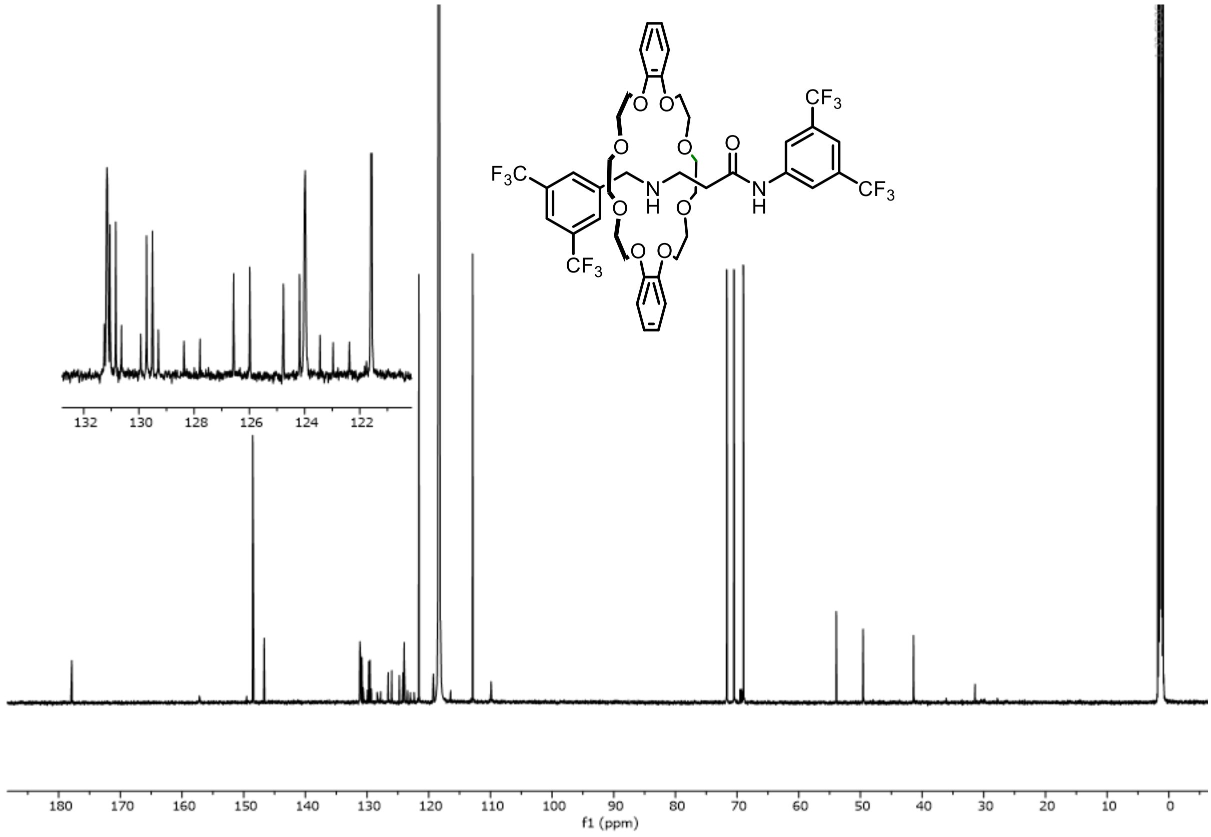

Figure S33 – ^{13}C NMR spectrum of 24C8⊂8 (151 MHz, CD_3CN).

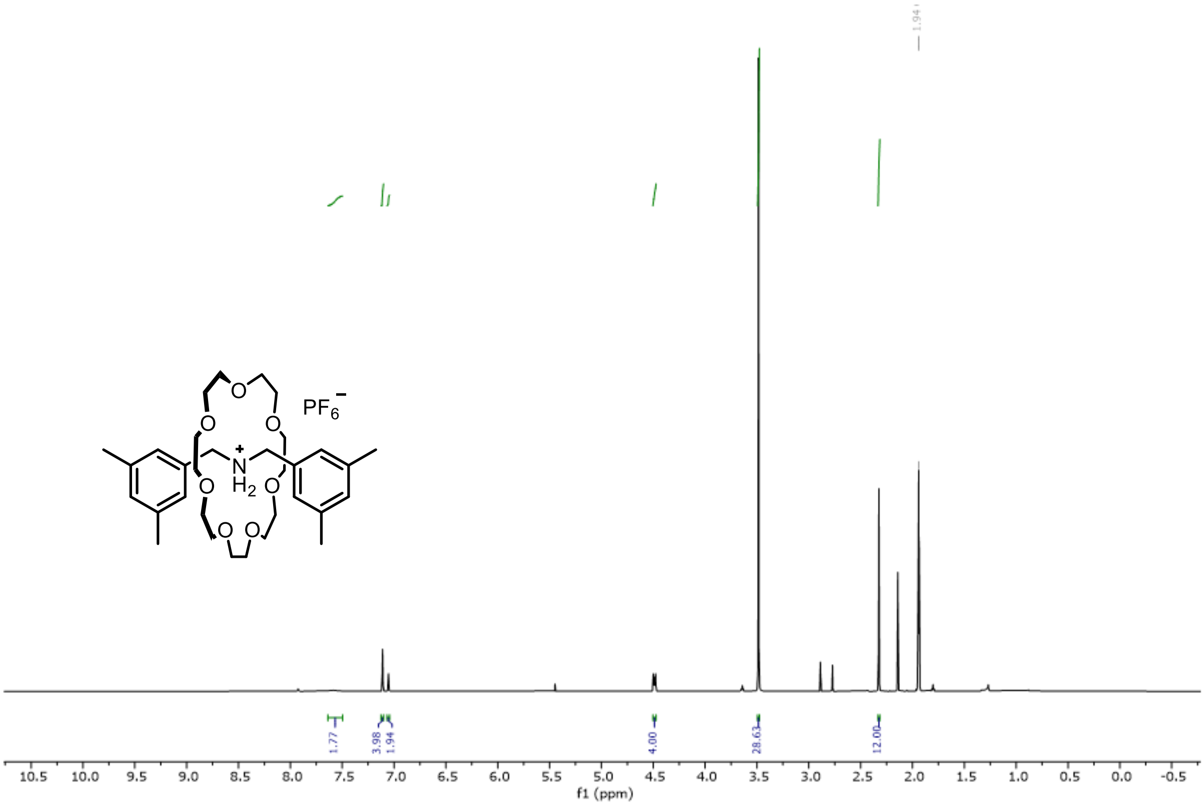

Figure S34 – ^{1}H NMR spectrum of 21C7⊂6•HPF$_6$ (600 MHz, CD$_3$CN).

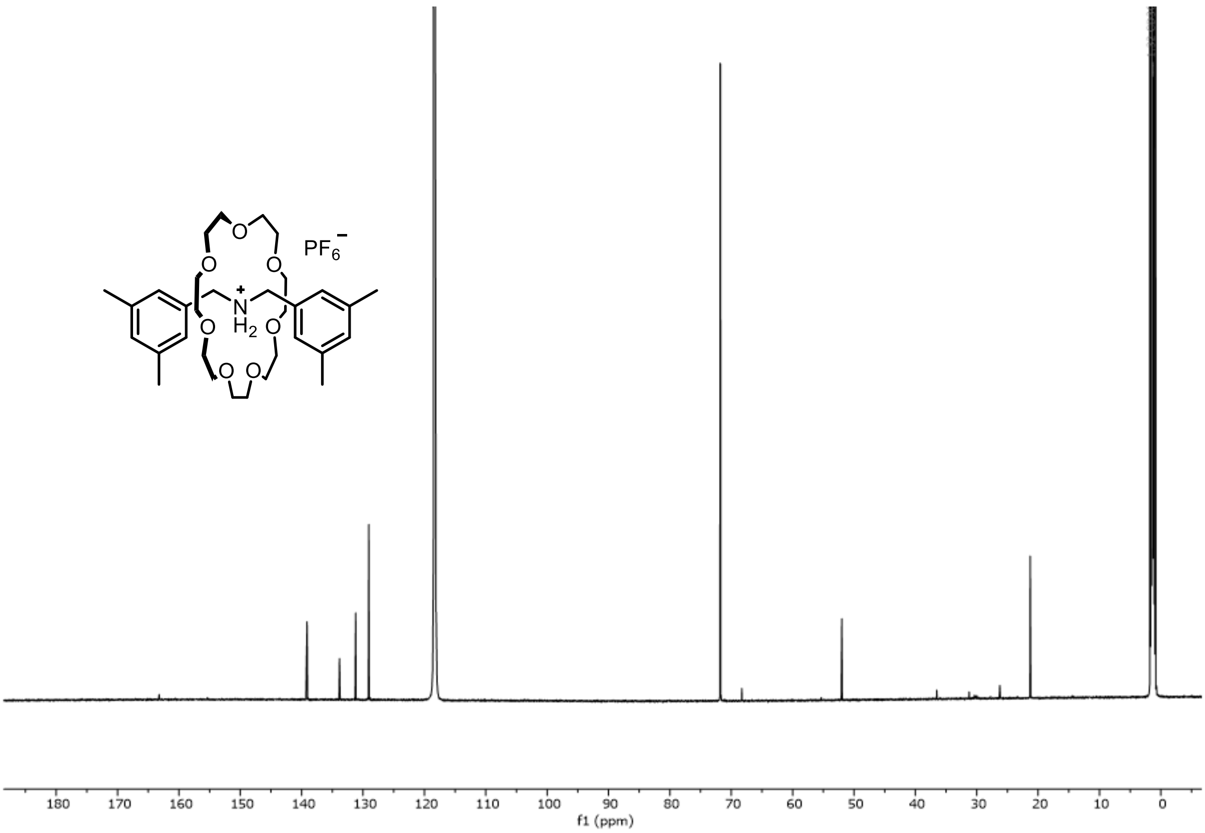

Figure S35 – ^{13}C NMR spectrum of 21C7⊂6•HPF$_6$ (151 MHz, CD$_3$CN).

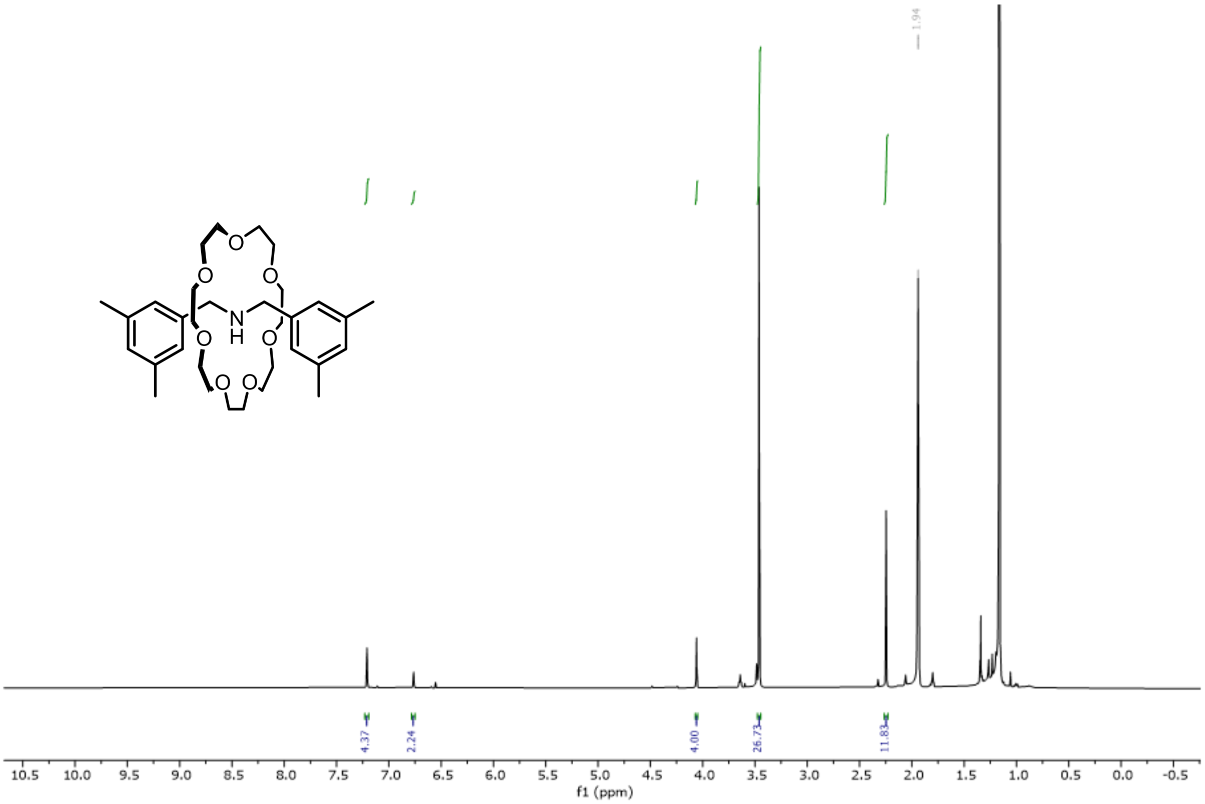

Figure S36 – ^{1}H NMR spectrum of 21C7⊂6 (600 MHz, CD_3CN).

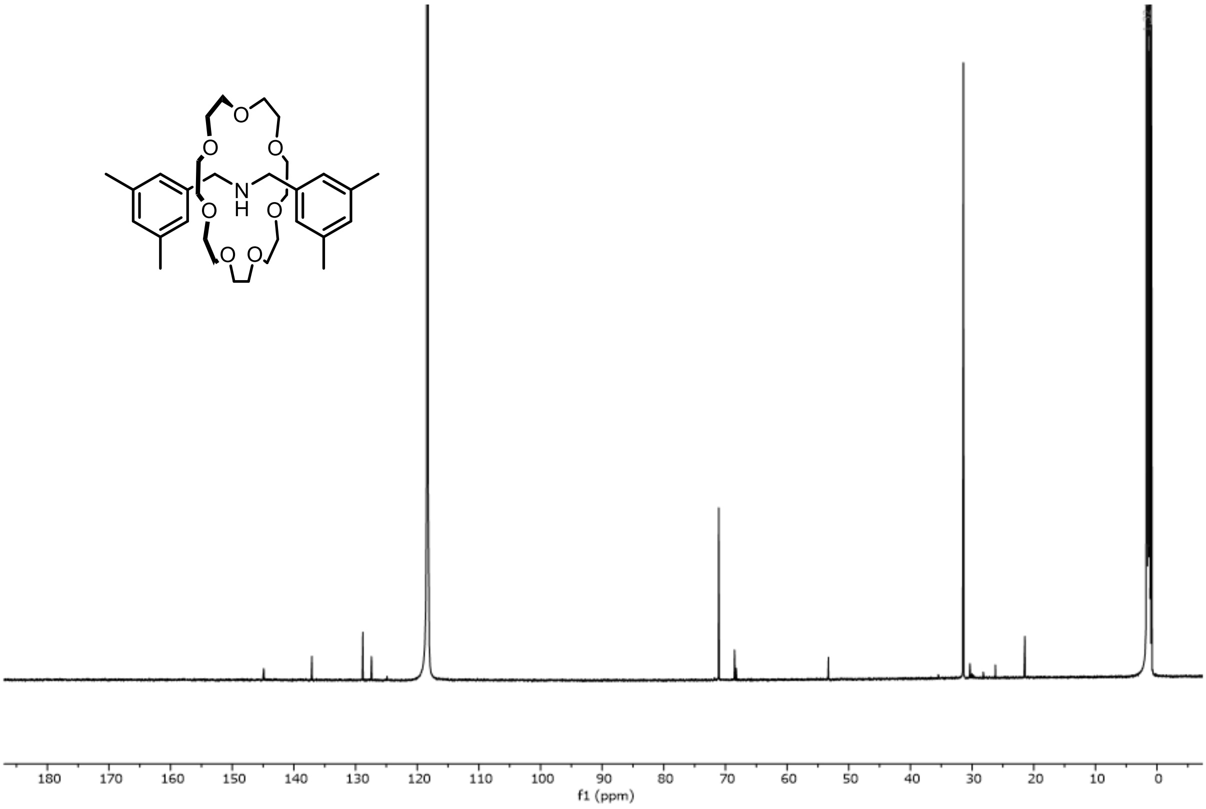

Figure S37 – ^{13}C NMR spectrum of 21C7⊂6 (151 MHz, CD_3CN).

7.0 Abbreviations

DBU – 1,8-Diazabicyclo[5.4.0]undec-7-ene

mTBD – 7-Methyl-1,5,7-triazabicyclo[4.4.0]dec-5-ene

TMG – 1,1,3,3-Tetramethylguanidine

P_1-*t*-Bu – *tert*-Butyliminotri(pyrollidino)phosphorane

P_2-Et – 1-Ethyl-2,2,4,4,4-pentakis(dimethylamino)-2λ^5,4λ^5-catenadi(phosphazene)

THF – tetrahydrofuran

DMF – *N*,*N*-dimethylformamide

NBS – *N*-bromosuccinimide

AIBN – Azoisobutyronitrile

PE – Petroleum ether (40–60 °C)

8.0 References

1. Sharma, D. M.; Punji, B. Selective Synthesis of Secondary Amines from Nitriles by a User-Friendly Cobalt Catalyst. *Adv. Synth. Catal.* **2019***, 361,* 3930–3936.
2. Gibson, H. W.; Bheda, M. C.; Engen, P.; Shen, Y. X.; Sze, J.; Zhang, H.; Gibson, M. D.; Delaviz, Y.; Lee, S.-H.; Liu, S.; Wang, L.; Nagvekar, D.; Rancourt, J.; Taylor, L. T. Synthesis and Characterization of Large (30-60-Membered) Aliphatic Crown Ethers. *J. Org. Chem.* **1994**, *59*, 2186–2196.
3. Villalonga-Barber, C.; Meligova, A. K.; Alexi, X.; Steele, B. R.; Kouzinos, C. E.; Screttas, C. G.; Katsanou, E. S.; Micha-Screttas, M.; Alexis, M. N. New hydroxystilbenoid derivatives endowed with neuroprotective activity and devoid of interference with estrogen and aryl hydrocarbon receptor-mediated transcription. *Bioorg. Med. Chem.* **2011**, *19*, 339–351.
4. Gilbert, S. H.; Fuentes, J. A.; Cordes, D. B.; Slawin, A. M. Z.; Clarke, M. L. *Eur. J. Org. Chem.* **2020**, 3071–3076.
5. Borodin, O.; Shchukin, Y.; Robertson, C. C.; Richter, S.; von Delius, M. Self-Assembly of Stimuli-Responsive [2]Rotaxanes by Amidinium Exchange. *J. Am. Chem. Soc.* **2021**, *143*, 16448–16457.
6. Fielden, S. P. D.; Leigh, D. A.; McTernan, C. T.; Pérez-Saavedra, B.; Vitorica-Yrezabal, I. J. Spontaneous Assembly of Rotaxanes from a Primary Amine, Crown Ether and Electrophile. *J. Am. Chem. Soc.* **2018**, *140*, 6049–6052.
7. Nasca, E. D.; Lambert, T. H. Higher-Order Cyclopropenimine Superbases: Direct Neutral Brønsted Base Catalyzed Michael Reactions with α-Aryl Esters. *J. Am. Chem. Soc.* **2015**, *137*, 10246–10253.
